# Long-term projections of cancer prevalence in Australia to 2050: a population-based statistical modelling study

**DOI:** 10.1016/j.lanwpc.2026.101946

**Published:** 2026-08-17

**Authors:** Qingwei Luo, David P. Smith, Michael David, Eleonora Feletto, Clare Kahn, Julia Steinberg, Michael Jefford, Karen Canfell

**Affiliations:** a The Daffodil Centre, The University of Sydney, and Cancer Council NSW, New South Wales, Australia; b School of Medicine & Dentistry, Griffith University, Queensland, Australia; c Cancer Elimination Collaboration, Sydney School of Public Health, The University of Sydney, New South Wales, Australia; d Centre for Health Services Research in Cancer, Australian Cancer Survivorship Centre and Department of Medical Oncology, Peter MacCallum Cancer Centre, Melbourne, Victoria, Australia; e Sir Peter MacCallum Department of Oncology, University of Melbourne, Parkville, Victoria, Australia

**Keywords:** Cancer prevalence, Statistical projections, Age-period-cohort model, Generalised linear model, Counting method, Cure proportion

## Abstract

**Background:**

Cancer prevalence is the number of people alive with a past cancer diagnosis in a specified number of previous years (e.g., last 5 or 30 years). This study estimates 1- to 30-year cancer prevalence in Australia for each year in 2025–2050, for 24 cancer types, the group of remaining cancers, and for all cancers combined.

**Methods:**

We used validated methods to estimate prevalence as a function of incidence and survival, using tabulated data from the Australian Institute of Health and Welfare and incorporating the effect of influential cancer-specific factors. The number of people living with cancer (cancers not yet cured) is estimated using the time-to-cure method.

**Findings:**

The 30-year prevalence is projected to increase by 57.1% from 1,666,020 (95% uncertainty interval [UI]: 1,553,825–1,784,900) in 2025 to 2,617,016 (95% UI: 2,333,949–2,947,174) in 2050. For people aged 80+ years, 30-year prevalence is projected to increase by 111.0% from 481,079 (95% UI: 448,799–515,286) in 2025 to 1,015,061 (95% UI: 917,670–1,125,036) in 2050. Breast cancer is projected to be the most prevalent cancer in Australia in 2050, followed by prostate, melanoma and colorectal cancer. For 30-year prevalence in 2050, 43.9% of people (1,149,364; 95% UI: 1,103,165–1,238,377) are estimated to be living with cancer (cancers not yet cured) and requiring initial treatment or ongoing care.

**Interpretation:**

A substantial increase in the number of people with a past cancer diagnosis in Australia is expected in 2025–2050. Expansion of appropriate services to meet the demand will be a key challenge for the health system in Australia.

**Funding:**

This work was not supported by a dedicated research grant. This work and open access publishing were supported by the 10.13039/501100001102Cancer Council NSW, through funding to the Daffodil Centre. KC is a recipient of a Fellowship from the 10.13039/501100000925National Health and Medical Research Council of Australia (APP1194679). JS is a recipient of a Cancer Institute NSW Career Development Fellowship (2022/CDF1154).

**Research in context**

**Evidence before this study**

Long-term projections of national cancer prevalence provide essential information for policymakers and health service planning. We searched MEDLINE, Embase, and PreMEDLINE on Oct 22, 2025, for articles published in English from Jan 1, 2015, that used statistical methods to project national 30-year or complete cancer prevalence, defined as the number of people alive at a specific time point with a past cancer diagnosis. Searches were conducted for all cancers combined and for individual cancer types with projections extending at least 15 years beyond the observed period, using the terms “cancer$ or neoplasm$ or tumo$” and “prevalence” and “projection$ or projected or forecast$ or predict$ or extrapolate$”. We identified one study reporting long-term projections of cancer prevalence for all cancers in the United States.

**Added value of this study**

We report long-term projections of cancer prevalence in Australia to 2050 for 24 specific cancer types, the group of remaining cancers, and for all cancers combined. Cancer prevalence was modelled as a function of cancer incidence and survival, with cancer incidence projections explicitly incorporating key cancer-specific factors, including cigarette smoking exposure for lung cancer, prostate-specific antigen testing rates for prostate cancer, and mammography screening participation rates for breast cancer. The 30-year prevalence of people with a past cancer diagnosis living in Australia is estimated to be 1.67 million in 2025, increasing by 57.1% to 2.62 million in 2050. By 2050, the prevalent population will be heterogeneous with respect to time since diagnosis, although more than 30% of people are projected to have been diagnosed within the past 5 years (0.79 million). Overall, our projections suggest that in 2050, while more than half of people with a past cancer diagnosis will be potentially cured (1.47 million), about 1.15 million people will be living with cancer (cancers not yet cured) and requiring active treatment or ongoing care.

**Implications of all the available evidence**

Our cancer prevalence projections can inform the prioritisation of cancer control programs, and support health service planning for future cancer care, treatment, and survivorship care in Australia. This study provides timely long-term projections of cancer prevalence for the period 2025–2050. Our findings highlight the challenges associated with meeting the needs of a large and growing population of people living with and beyond cancer. In particular, we project a substantial increase in the number of people aged 60 years and over with a past cancer diagnosis, many of whom may also be affected by multimorbidity. This underscores the need for a well-integrated health system to effectively manage cancer alongside co-morbidities in the coming years. Substantial expansion and adaptation of health services will be required to meet this growing demand.

## Introduction

Australia is one of the countries with the highest age-standardised cancer incidence rates in the world,^1^ and previous predictions suggest that over 4.56 million new cancer diagnoses are expected over the 25-year period from 2020 to 2044.^2^ It is also projected that cancer will remain the leading cause of premature death in Australians through to 2044.^3^ These estimates emphasise the need for an ongoing focus on cancer prevention, early detection and effective treatments, but further understanding of cancer prevalence is also essential for the planning of health services to meet the needs of all those living with or beyond cancer. Cancer prevalence is commonly defined as the number or proportion of people in a population who have ever been diagnosed with a cancer and who are alive at a specific time point.^4^^,^^5^ The measure reflects the true magnitude of the cancer burden, and as a sub-measure, those living with cancers not yet cured at the time reflects the number of people who may require access to health services or cancer care. Therefore, estimating detailed future cancer prevalence by likely survivorship status (i.e., in the initial treatment phase or requiring ongoing care, or post-cure and not requiring treatment) is particularly critical for informing policy and cancer control strategies.

Long-term estimates of cancer prevalence capture a diverse group in terms of including individuals affected by cancer who are at different phases of their disease journey, from initial diagnosis through to long-term survivorship. Estimating cancer prevalence requires data on how many people in the population were diagnosed with a cancer at a specific time point and how many of these people remain alive after a certain number of years since diagnosis. Cancer prevalence is often reported as limited duration prevalence (e.g., numbers of people with a past cancer diagnosis in the last 5, 10 or 30 years prior to a specific year). As most population-based cancer registries in the world were established in the past few decades, prevalence estimates generally cannot capture all prior cancer diagnoses in the population.^4^^,^^5^ Nevertheless, a long period such as 30-years for prevalence may serve as a proxy for all people with a past cancer diagnosis, given that the majority of cancers are diagnosed in middle or older adulthood. We acknowledge that 30-year prevalence may still miss long-term survivors diagnosed more than 30 years earlier, especially for cancers with excellent survival outcomes. We previously developed and validated a modified counting method for estimating 1-year to 30-year cancer prevalence as a function of the number of new diagnoses and survival rates for multiple myeloma.^6^ This method was also applied to another study projecting 1-year to 5-year cancer prevalence for 22 solid cancers and all solid cancers combined.^7^ However, there have not yet been any published long-term projections of 1-year to 5-year cancer prevalence for other blood cancers and all cancers combined, and 30-year prevalence projections are not available for most cancer types or all cancers combined in Australia.

The aim of this study is to estimate the future prevalence of people in Australia with a past cancer diagnosis in the last 1, 5 and 30 years and alive in calendar years 2025–2050, for 24 individual cancer types, the group of remaining cancers, and all cancers combined. These 24 cancer types/groups accounted for 92.3% of all cancer diagnoses in 2019.^5^ These cancer types were selected based on the following criteria: (1) long-term observed incidence data are available and robust incidence projection models could be validated, (2) observed survival data (needed to estimate prevalence) are available, and (3) prevalence estimates could be validated.

## Methods

### Ethics approval

This population-based statistical modelling study used tabulated data for cancer incidence, survival and prevalence, released by the Australian Institute of Health and Welfare (AIHW).^5^ Ethics approval and patient consent were not required to use these publicly available aggregated data.

### Data sources

Supplementary Fig. S1 provides a summary of the data sources used in this study. Data on new diagnoses (1982–2019), survival (1986–2020), and prevalence (2000–2020) for 24 individual cancer types, the group of remaining cancers referred to here as ‘other cancers’ and for all cancers combined by sex, age group and calendar year were obtained from the AIHW (International Classification of Diseases 10th Revision codes are provided in Supplementary Table S1).^5^ In this study, prevalence estimates for all cancers combined represent counts of unique individuals with a past cancer diagnosis, while prevalence estimates for selected cancer types include individuals who may have multiple primary cancers. Other tabulated data used in this study include cigarette smoking exposure,^8^ participation rates in the breast cancer screening program, and prostate-specific antigen (PSA) testing rates.^9^ Australian population data from 1982 to 2050 were obtained from the Australian Historical Population Statistics and Population Projections (Series B, assuming medium population growth) produced by the Australian Bureau of Statistics^10^^,^^11^ (Supplementary material 1).

### Outcomes of interest

In this study, annual prevalence was defined as the number of individuals alive in a calendar year with a past cancer diagnosis within a specified duration of follow-up after initial diagnosis (Supplementary Fig. S2). For example, 30-year prevalence estimates for colorectal cancer in 2025 include the number of people alive in 2025 who had been diagnosed with colorectal cancer within the previous 30 years (1995–2025). In this study, we used the 30-year prevalence as a proxy for total prevalence, 5-year prevalence as a proxy for people requiring monitoring and potentially ongoing care, and 1-year prevalence as a proxy for the number of people receiving initial cancer treatment.

In addition, we defined two distinct outcome measures to capture different broad phases of survival: (1) the number of individuals ‘living with cancer’ (cancers not yet cured at the time), including people requiring initial cancer treatment or ongoing care; (2) the number of individuals ‘living beyond cancer’ (potentially cured), defined as people who were alive and whose time since initial diagnosis reached or exceeded the estimated “time-to-cure”, so they are likely to be cured and die from other causes in line with the general population.^12^^,^^13^

### Statistical analysis

This study adhered to the reporting guideline for epidemic forecasting and prediction research as outlined in the EPIFORGE 2020 checklist.^14^ Statistical analyses were performed using Stata, version 18, Stata Corporation, College Station, TX, using the *apcspline, glm, ipolate* commands.

#### Prevalence projections

A more detailed description of our previously developed and validated modified counting method to estimate prevalence is available elsewhere^6^^,^^7^ and is also provided in Supplementary material 3. Briefly, there were three steps of analyses involved in this study (Supplementary Fig. S1):

(1) Cancer incidence projections: We previously developed and validated generalised linear or age-period-cohort (APC) models incorporating smoking intensity, breast cancer screening participation rates, and PSA testing rates.^2^^,^^7^ Other screening participation factors for colorectal, cervical and lung cancers were not incorporated in the final projection models as the contemporaneous participation data were unavailable for the time period required for the statistical modelling (see Supplementary material 1.3 for additional details). In the current study, we re-fitted the incidence projection models for 21 cancer types/groups by sex, 5-year age group, and calendar year to 2050, and included two additional cancer types (cervix and unknown primary site) and additionally developed a model for ovarian/tubal serous cancer to account for the impact of the classification change on recent trends in ovarian cancer^5^ (Supplementary material 3.1).
(2) Survival projections: we extrapolated the observed sex-age-specific survival rates forward to 2029 (Supplementary material 3.2), to account for the potential ongoing impact of improved treatment and the benefits of earlier detection through screening programs (e.g., stage shift), then assumed that survival will remain constant to 2050 (Supplementary Fig. S5).
(3) Cancer prevalence projections: we then estimated 1-year, 5-year and 30-year prevalence as a function of incidence and survival (Supplementary material 3.3).^6^^,^^7^

The uncertainty intervals (UIs) for the estimated prevalence were estimated based on the UIs of the number of new cases and survival rates. For example, the lower bound of the UI for prevalence was estimated using the lower bound of the number of new cases and the lower bound of the survival estimates (see Supplementary material 3.4 for additional details). This assumes a perfect correlation between incidence and survival (i.e., the lowest/highest incidence bound is associated with the lowest/highest survival bound), which produces wider UIs for prevalence than if a smaller correlation is assumed. Thus, the UI estimates for prevalence in this study are considered to be conservative. Due to the use of the log-transformations of survival estimates and the non-linear period and cohort effects in the APC models, the reported UIs show numerical asymmetry relative to the point estimate.

#### Validation of prevalence estimates

The method for estimating prevalence at different durations was validated by comparing the estimated prevalence for each of the 24 cancer groups and all cancers combined in this study with the cancer prevalence in Australia in 2000–2020 reported by the AIHW.^5^ The estimated prevalence in 2000–2020 was in good agreement with the corresponding reported prevalence,^5^ with low relative differences (median [interquartile range] of differences 3.6% [1.8–5.0%] for 5-year and 4.4% [1.9–6.6%] for 10-year prevalence; Supplementary Figs. S6 and S7).

#### Estimating the number of people living beyond cancer (potentially cured)

For each cancer type and sex, we estimated the “number of people living beyond cancer (potentially cured)” by summing the number of people who were alive and whose time since initial diagnosis reached or exceeded the estimated “time-to-cure” (Supplementary material 4).^13^ For each cancer type and sex, we defined the “time-to-cure” *t* as the number of years after initial cancer diagnosis at which the 5-year conditional survival estimate exceeds 95%,^15^ or exceeds 90% and reaches a plateau. In other words, we defined time-to-cure based on the time at which survival in those with a past cancer diagnosis becomes close-to-equivalent to the background general population rates. This approach is based on available population-level data and represents a statistical rather than clinical definition of cure; notably, it does not imply a total absence of clinical risk of recurrence (especially for cancers where late recurrences may occur). The cure proportion was then estimated by dividing the number of people living beyond cancer (potentially cured), by the total 30-year prevalence.

#### Sensitivity analyses

We conducted four sensitivity analyses by estimating the prevalence under different assumptions: (1) survival remains constant from 2019, which is the last observed year (a highly conservative scenario); (2) the increase in future extrapolated survival levels off from 2024 (5 years after the last observed data, as an intermediate plateau point); (3–4) the increase in the future extrapolated survival levels off from 2034 or from 2039 (two scenarios with continued survival improvements over a further 15 or 20 years after the last observed data in 2019, respectively). We also conducted eight sensitivity analyses for estimating the number of people living beyond cancer (potentially cured) by applying alternative thresholds for 5-year conditional relative survival (exceeding 90–98% or exceeding 86–94% and reaching a plateau) (Supplementary material 5).

### Role of the funders

This work was not supported by a dedicated research grant. The providers of funding received by individual authors had no role in study design, data collection, data analysis, data interpretation, or writing of the report.

## Results

**Fig. 1:**
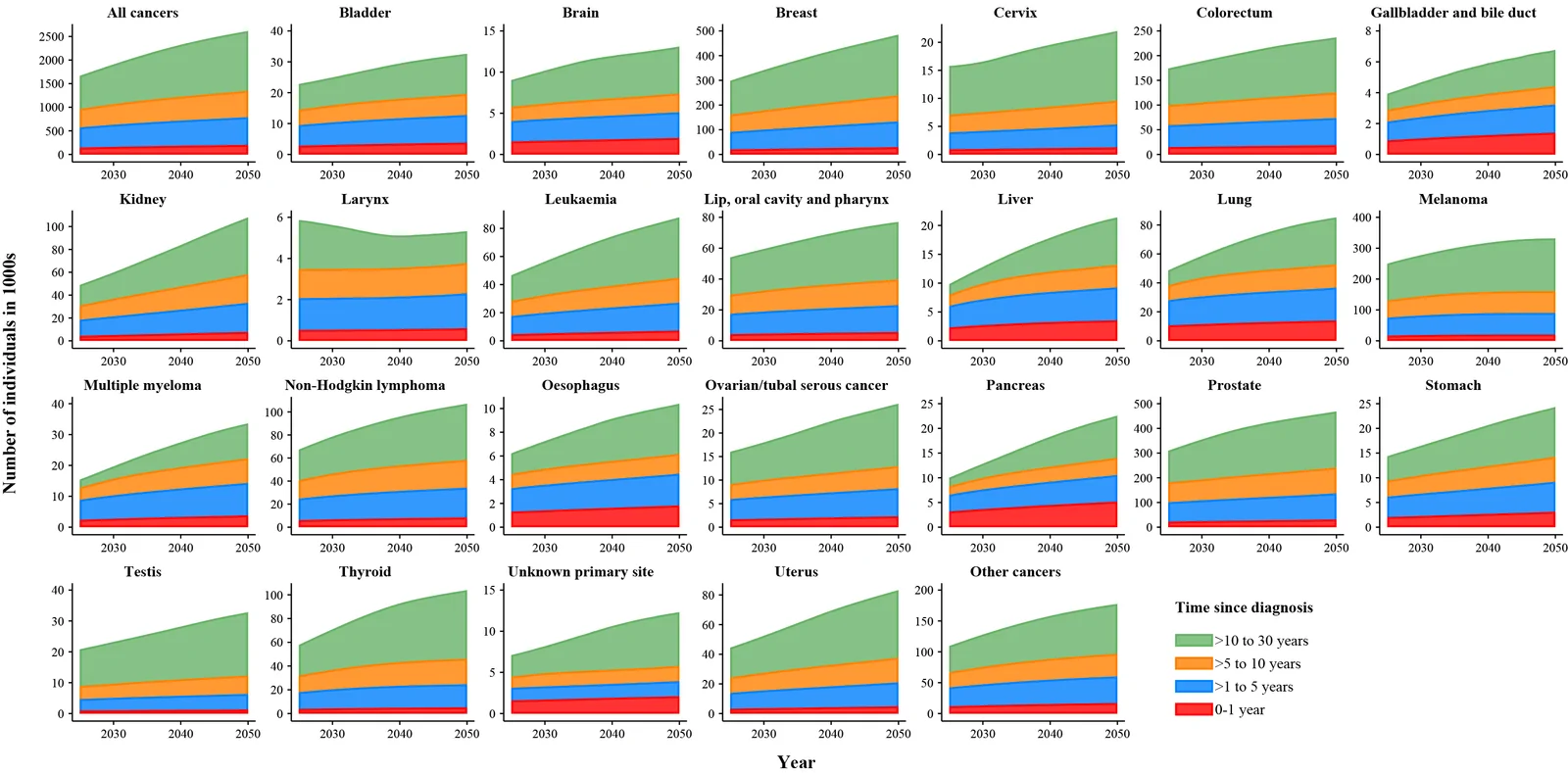
**Estimated annual cancer prevalence for all cancers combined and selected cancer types, by time since diagnosis in Australia, 2025–2050.** Annual prevalence was defined as the number of individuals alive in a calendar year with a past cancer diagnosis within a specified duration of follow-up after initial diagnosis.

**Fig. 2:**
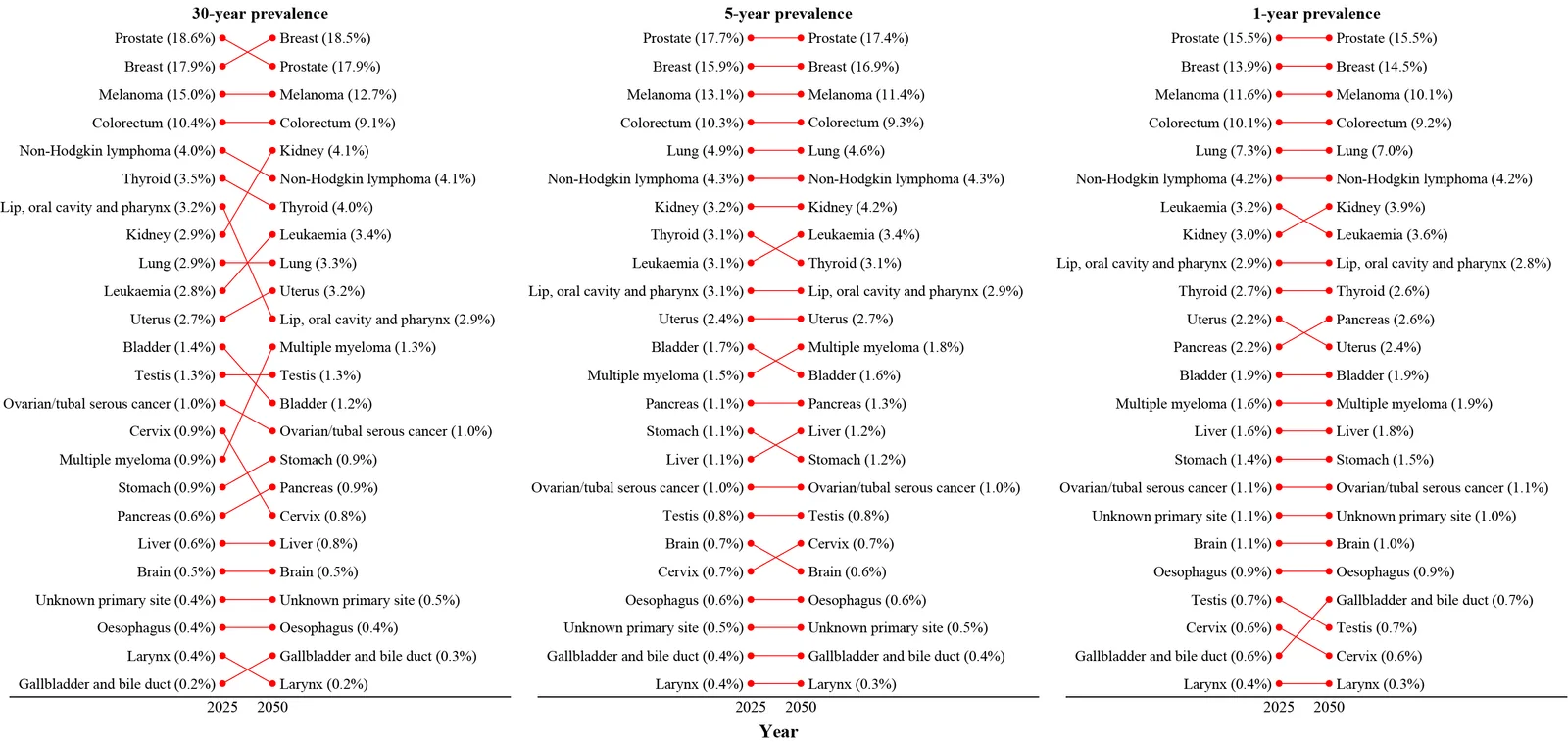
**Change in rank of cancer types sorted by decreasing cancer prevalence over the period 2025–2050, Australia.** Percentages are of the total cancer prevalence.

The prevalence of people with a past cancer diagnosis in the last 30 years of an initial diagnosis is projected to increase by 57.1% from 1,666,020 (95% UI: 1,553,825–1,784,900) in 2025 to 2,617,016 (95% UI: 2,333,949–2,947,174) in 2050 (Table 1). Among cancer survivors in 2050, 69.8% (1,826,666, 95% UI 1,630,636–2,054,837) are expected to be diagnosed more than 5 years previously (Fig. 1 and Supplementary Table S4). For 30-year prevalence in 2050, breast cancer is estimated to be the most prevalent cancer in Australia, followed by prostate cancer, melanoma and colorectal cancer. For both 1-year and 5-year prevalence, prostate cancer is estimated to be the most prevalent cancer in 2050, followed by breast cancer, melanoma and colorectal cancer (Table 1 and Fig. 2). For 30-year prevalence in 2050, prostate, melanoma, colorectal and kidney cancers are the most prevalent cancers for males, while breast, melanoma, colorectum and uterus are the most prevalent cancers for females (Supplementary Fig. S9). The 30-year prevalence estimates for all cancers combined and for most cancer types are consistently higher for males than those for females, except for pancreatic, thyroid and lung cancers (Supplementary Fig. S10 and Table S5).

**Table 1:** Estimated cancer prevalence for all cancers combined and selected cancer types, by prevalence duration in Australia, 2025–2050.

Cancer type	30-year prevalence			5-year prevalence			1-year prevalence		
	Counts (95% UI) in 2025	Counts (95% UI) in 2050	Change %^a^	Counts (95% UI) in 2025	Counts (95% UI) in 2050	Change %^a^	Counts (95% UI) in 2025	Counts (95% UI) in 2050	Change %^a^
**All cancers**	**1,666,020 (1,553,825–1,784,900)**	**2,617,016 (2,333,949–2,947,174)**	**57.1**	**571,936 (529,203–619,363)**	**790,350 (703,313–892,337)**	**38.2**	**145,887 (135,133–157,824)**	**203,036 (181,570–227,989)**	**39.2**
Bladder	22,823 (20,094–25,681)	32,623 (27,019–39,408)	42.9	9472 (8455–10,625)	12,797 (10,661–15,362)	35.1	2804 (2532–3110)	3851 (3266–4542)	37.3
Brain	9034 (7177–11,128)	13,111 (9897–17,318)	45.1	4025 (3372–4815)	5126 (4054–6507)	27.4	1562 (1361–1797)	1997 (1658–2419)	27.8
Breast	298,109 (288,918–307,472)	485,135 (460,470–511,175)	62.7	90,991 (87,896–94,197)	133,590 (126,172–141,468)	46.8	20,225 (19,525–20,950)	29,475 (27,884–31,160)	45.7
Cervix	15,776 (13,935–17,789)	22,023 (17,050–28,509)	39.6	3927 (3326–4637)	5352 (4002–7152)	36.3	917 (771–1088)	1266 (954–1676)	38.1
Colorectum	173,993 (163,351–185,160)	237,053 (209,185–269,057)	36.2	58,879 (54,876–63,207)	73,754 (64,792–84,096)	25.3	14,777 (13,760–15,881)	18,607 (16,460–21,069)	25.9
Gallbladder and bile duct	3930 (2895–5005)	6778 (4813–9345)	72.5	2118 (1720–2605)	3235 (2459–4234)	52.7	911 (775–1073)	1417 (1141–1764)	55.5
Kidney	48,908 (43,934–54,266)	108,101 (91,074–128,515)	121.0	18,394 (16,497–20,553)	33,236 (27,831–39,746)	80.7	4404 (3949–4919)	7830 (6611–9292)	77.8
Larynx	5871 (4818–6928)	5330 (4002–7200)	−9.2	2070 (1724–2506)	2308 (1760–3062)	11.5	530 (451–635)	614 (475–789)	15.8
Leukaemia	46,594 (41,695–51,950)	87,858 (76,746–101,225)	88.6	17,543 (15,931–19,397)	27,130 (24,015–30,861)	54.6	4676 (4305–5104)	7292 (6573–8144)	55.9
Lip, oral cavity and pharynx	54,051 (49,099–59,056)	77,046 (65,052–91,344)	42.5	17,473 (15,730–19,407)	23,079 (19,226–27,733)	32.1	4284 (3852–4763)	5696 (4788–6784)	33.0
Liver	9822 (7942–12,146)	21,411 (15,643–29,657)	118.0	6038 (5123–7193)	9265 (7016–12,402)	53.4	2321 (2013–2693)	3560 (2797–4581)	53.4
Lung	48,620 (44,101–53,411)	85,284 (73,647–98,657)	75.4	27,903 (25,746–30,226)	36,751 (32,058–42,132)	31.7	10,655 (9915–11,452)	14,122 (12,511–15,953)	32.5
Melanoma	249,892 (238,560–261,519)	331,909 (306,494–360,106)	32.8	74,724 (70,935–78,777)	90,295 (83,094–98,296)	20.8	16,987 (16,119–17,918)	20,419 (18,867–22,134)	20.2
Multiple myeloma	15,420 (13,252–17,825)	33,636 (27,745–40,816)	118.1	8834 (7896–9873)	14,343 (12,185–16,959)	62.4	2381 (2157–2626)	3844 (3332–4458)	61.4
Non-Hodgkin lymphoma	67,310 (61,242–73,826)	107,133 (91,522–125,644)	59.2	24,699 (22,424–27,263)	34,274 (29,199–40,302)	38.8	6098 (5539–6728)	8437 (7254–9828)	38.4
Oesophagus	6219 (5031–7526)	10,405 (8253–12,974)	67.3	3274 (2828–3783)	4517 (3756–5422)	38.0	1306 (1167–1465)	1839 (1583–2129)	40.8
Ovarian/tubal serous cancer	16,005 (13,902–18,347)	26,219 (20,775–33,306)	63.8	5957 (5196–6842)	8272 (6539–10,567)	38.9	1660 (1464–1888)	2312 (1874–2874)	39.3
Pancreas	10,003 (8431–11,632)	22,600 (18,573–27,287)	125.9	6535 (5798–7342)	10,569 (9011–12,369)	61.7	3177 (2902–3479)	5191 (4570–5896)	63.4
Prostate	309,663 (295,376–324,818)	468,991 (434,135–507,283)	51.5	101,035 (94,030–108,743)	137,140 (128,217–146,837)	35.7	22,591 (21,018–24,319)	31,494 (29,583–33,565)	39.4
Stomach	14,351 (12,355–16,481)	24,328 (20,435–28,844)	69.5	6147 (5504–6870)	9195 (7911–10,675)	49.6	2053 (1886–2242)	3133 (2777–3534)	52.6
Testis	20,830 (19,251–22,497)	32,837 (28,974–37,236)	57.6	4684 (4233–5182)	6364 (5540–7313)	35.9	986 (888–1094)	1322 (1150–1515)	34.1
Thyroid	57,828 (52,817–63,049)	103,965 (88,027–123,107)	79.8	17,923 (16,064–20,032)	24,651 (20,388–29,889)	37.5	3916 (3490–4407)	5210 (4318–6304)	33.0
Unknown primary site	7105 (5709–8680)	12,314 (9508–15,867)	73.3	3123 (2623–3707)	3932 (3041–5074)	25.9	1602 (1399–1834)	2107 (1704–2612)	31.5
Uterus	44,447 (41,573–47,425)	83,281 (74,862–92,631)	87.4	13,917 (12,919–14,995)	21,036 (18,778–23,564)	51.2	3268 (3035–3521)	4909 (4407–5465)	50.2
Other cancers	109,416 (98,367–121,283)	177,645 (150,048–210,663)	62.4	42,251 (38,357–46,586)	60,139 (51,608–70,315)	42.3	11,796 (10,860–12,838)	17,092 (15,033–19,502)	44.9

UI: uncertainty interval.

a Overall percentage change in the prevalence for 2050 compared to the prevalence for 2025.

**Fig. 3:**
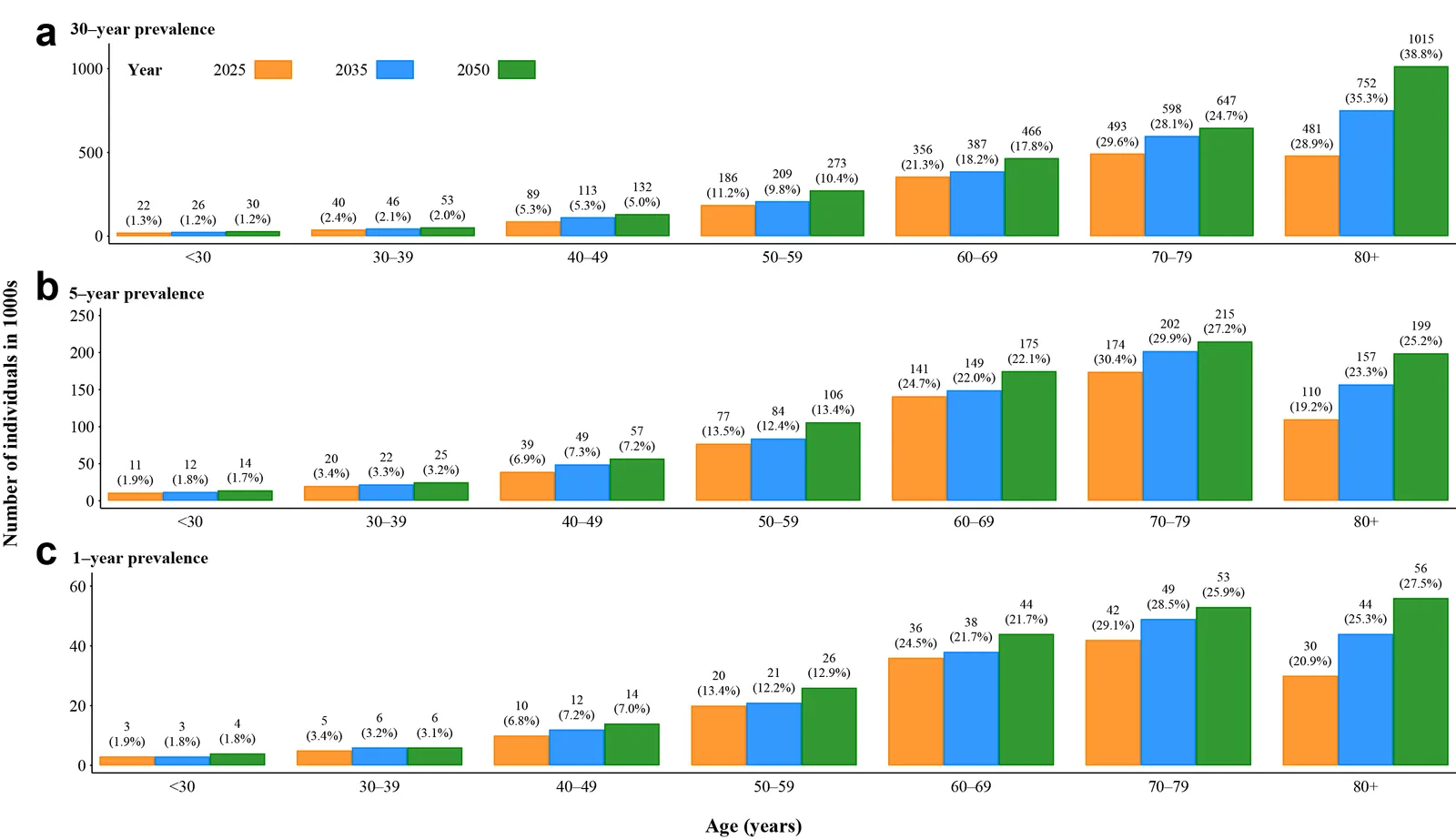
**Change in age distribution for cancer prevalence in Australia, 2025–2050**. (a). 30-year prevalence; (b). 5-year prevalence; (c). 1-year prevalence.

The age distribution of prevalent individuals is estimated to vary by duration of prevalence and is projected to change over time (Table 2, Fig. 3 and Supplementary Table S6). In 2025, people aged 70–79 years are estimated to contribute the largest proportion across all durations of prevalence, while the age structure is projected to shift towards a higher proportion of people aged 80+ in 2050 (Fig. 3). From 2025 to 2050, the 30-year prevalence is projected to increase by 111.0% from 481,079 (95% UI 448,799–515,286) to 1,015,061 (95% UI 917,670–1,125,036) for those aged 80+ years, respectively. Similar patterns are projected for 1-year and 5-year prevalence (Supplementary Fig. S11 and Table S7).

**Table 2:** Estimated cancer prevalence by age group and prevalence duration in Australia, 2025 and 2050.

	30-year prevalence			5-year prevalence			1-year prevalence		
	Number of people with a past cancer diagnosis (95% UI)	% of total people with a past cancer diagnosis	% of all Australians of that age group	Number of people with a past cancer diagnosis (95% UI)	% of total people with a past cancer diagnosis	% of all Australians of that age group	Number of people with a past cancer diagnosis (95% UI)	% of total people with a past cancer diagnosis	% of all Australians of that age group
Year 2025									
<15 years	5096 (3815–6716)	0.3	0.1	2849 (2090–3911)	0.5	0.1	711 (523–977)	0.5	0.0
15–29 years	17,122 (13,663–20,738)	1.0	0.3	7850 (6427–9686)	1.4	0.1	2132 (1785–2575)	1.5	0.0
30–39 years	39,760 (34,836–44,884)	2.4	1.0	19,689 (17,657–22,030)	3.4	0.5	4950 (4453–5514)	3.4	0.1
40–49 years	88,711 (80,644–97,202)	5.3	2.5	39,320 (35,928–43,113)	6.9	1.1	9927 (9078–10,882)	6.8	0.3
50–59 years	185,874 (172,733–199,804)	11.2	5.8	77,437 (71,583–83,914)	13.5	2.4	19,553 (18,041–21,229)	13.4	0.6
60–69 years	355,599 (333,190–379,443)	21.3	12.3	141,271 (130,885–152,696)	24.7	4.9	35,782 (33,108–38,725)	24.5	1.2
70–79 years	492,779 (466,145–520,827)	29.6	22.7	173,645 (163,020–185,100)	30.4	8.0	42,412 (39,873–45,156)	29.1	2.0
80+ years	481,079 (448,799–515,286)	28.9	38.9	109,875 (101,613–118,913)	19.2	8.9	30,420 (28,272–32,766)	20.9	2.5
Total	**1,666,020 (1,553,825–1,784,900)**	**100.0**	**6.0**	**571,936 (529,203–619,363)**	**100.0**	**2.0**	**145,887 (135,133–157,824)**	**100.0**	**0.5**
Year 2050									
<15 years	6565 (4355–10,052)	0.3	0.1	3601 (2396–5508)	0.5	0.1	889 (598–1340)	0.4	0.0
15–29 years	23,710 (17,023–33,705)	0.9	0.3	10,150 (7678–13,759)	1.3	0.1	2741 (2164–3549)	1.4	0.0
30–39 years	53,253 (44,014–65,389)	2.0	1.1	25,327 (21,821–29,591)	3.2	0.5	6288 (5458–7299)	3.1	0.1
40–49 years	131,684 (113,043–154,481)	5.0	2.7	56,759 (49,300–65,747)	7.2	1.2	14,305 (12,467–16,506)	7.0	0.3
50–59 years	273,136 (239,547–312,788)	10.4	6.1	105,740 (93,238–120,359)	13.4	2.3	26,209 (23,217–29,670)	12.9	0.6
60–69 years	466,358 (414,479–526,197)	17.8	12.2	174,601 (155,130–197,042)	22.1	4.6	44,060 (39,233–49,603)	21.7	1.2
70–79 years	647,249 (583,818–719,526)	24.7	22.7	215,208 (194,934–238,358)	27.2	7.5	52,658 (47,818–58,161)	25.9	1.8
80+ years	1,015,061 (917,670–1,125,036)	38.8	41.8	198,964 (178,816–221,973)	25.2	8.2	55,886 (50,615–61,861)	27.5	2.3
Total	**2,617,016 (2,333,949–2,947,174)**	**100.0**	**7.1**	**790,350 (703,313–892,337)**	**100.0**	**2.1**	**203,036 (181,570–227,989)**	**100.0**	**0.5**

Percentages may not sum to 100% due to rounding.

**Fig. 4:**
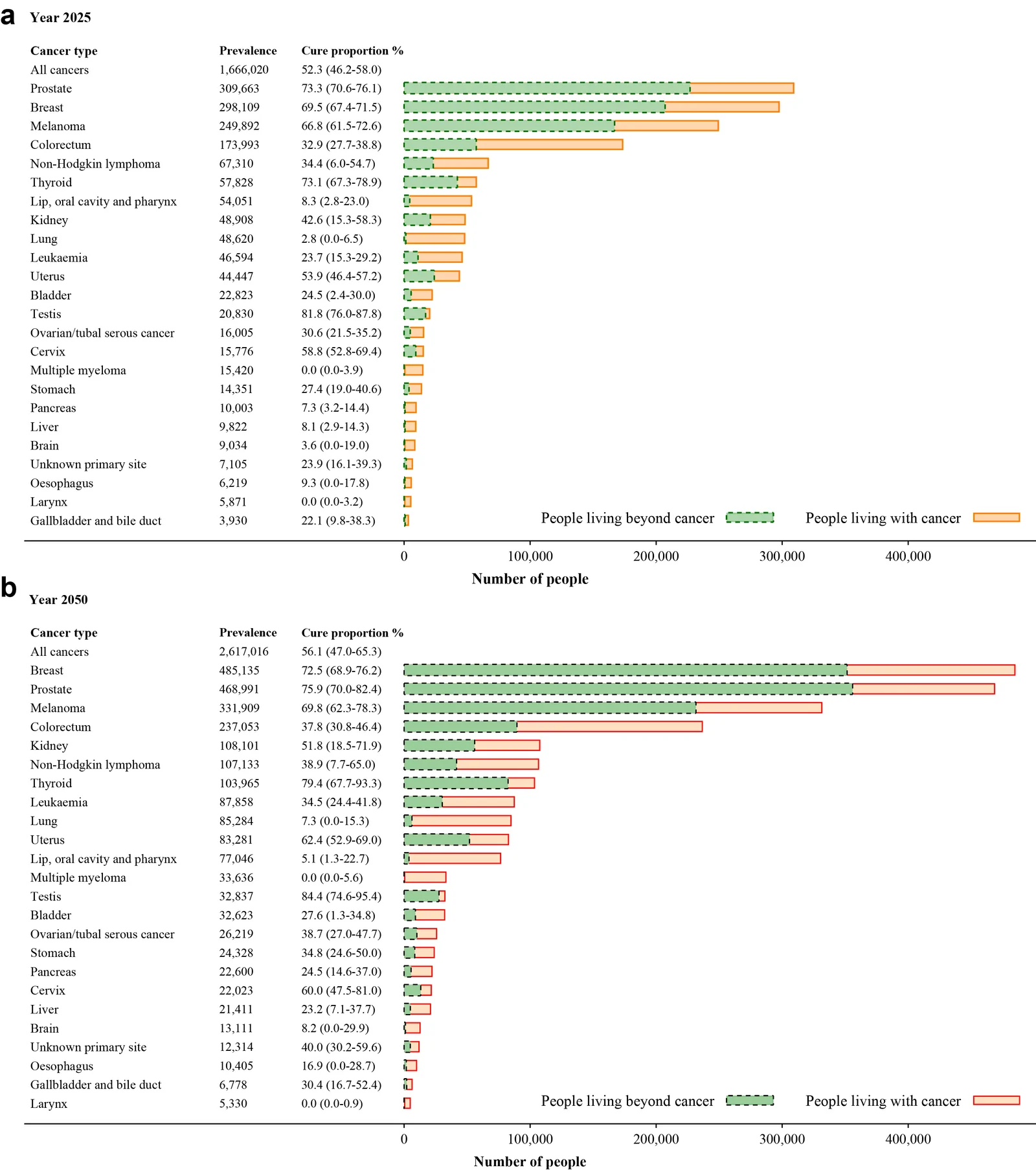
**People living with cancer (cancers not yet cured) and living beyond cancer (potentially cured) ranked by estimated 30-year cancer prevalence, 2025(a)and 2050(b).** People living with cancer (cancers not yet cured) refers to people with a past cancer diagnosis, requiring initial treatment or ongoing care for cancer. People living beyond cancer (potentially cured) refers to people with a past cancer diagnosis whose time since initial diagnosis reached or exceeded the estimated “time-to-cure” (at which 5-year conditional relative survival reaches 95%, or exceeds 90% and plateaus). The cure proportion was estimated by dividing the number of people living beyond cancer (potentially cured), by the total 30-year prevalence. The 95% uncertainty interval is shown in parentheses. The bar for “All cancers” is intentionally omitted from the figure for reasons of scale, as its substantially larger values (1.67 million in 2025 and 2.62 million in 2050) would significantly exceed the chart's axis given the scale required to show the patterns for individual cancer types.

For 30-year prevalence, the proportion of people living beyond cancer (potentially cured) is estimated to increase from 52.3% (95% UI 46.2–58.0%) in 2025 to 56.1% (95% UI 47.0–65.3%) in 2050 (Fig. 4). The proportions of people living beyond cancer in 2050 are estimated to be the highest for testicular (84.4%), thyroid (79.4%), prostate (75.9%), breast (72.5%) cancers and melanoma (69.8%). The proportion of people living beyond cancer is estimated to be slightly higher for females (59.2%, 95% UI 51.2–67.0%) than for males (53.1%, 95% UI 43.0–63.7%) (Supplementary Fig. S12). By contrast, 1,149,364 (95% UI 1,103,165–1,238,377) individuals are estimated to be ‘living with cancer’ (cancers not yet cured) in 2050 and requiring initial treatment or ongoing care (Supplementary Table S8).

In sensitivity analyses with more conservative or optimistic assumptions for future survival (remaining unchanged at the level observed in 2019, continuing to increase to 2024 only, or continuing to increase to 2034 and 2039, versus to 2029 in the main analysis), the differences in prevalence estimates compared to the main analyses are small for most cancer types, as survival has been relatively stable in the two most recent observed 5-year periods. However, these sensitivity analyses yield notable differences in prevalence estimates for multiple myeloma and liver, lung and pancreatic cancers, due to the substantial increases in survival rates for these cancer types over the last observed period (Supplementary Fig. S13).

In sensitivity analyses using alternative lower or higher survival thresholds to determine the cure proportion, we found only small relative changes to the point estimates of people living beyond cancer (potentially cured) for most cancer types except cancers of the bladder, brain, colorectum, kidney, lip, oral cavity and pharynx, non-Hodgkin lymphoma and leukaemia (Supplementary Fig. S14).

## Discussion

To our knowledge, this is the first study to provide comprehensive long-term projections of the number of people living with or beyond cancer in Australia for all cancers combined. We have estimated the prevalence of people living in Australia with a past cancer diagnosis in the last 30 years. The 30-year cancer prevalence in Australia is estimated to increase by three-fifths from 1.67 million in 2025 to 2.62 million people in 2050. This projected large increase is likely a result of multiple factors, including an increase in the number of new cancer diagnoses over time due to population growth and ageing, as well as increases in incidence rates for many cancer types.^2^ In addition, improved survival outcomes over time that were projected into the future may have also contributed to this expected increase in prevalence, presumably due to advances in treatment and early detection through cancer screening programs.^16^ For all cancers combined, our projections suggest that in 2050, although over half of people with a past cancer diagnosis will be potentially cured, the numbers of people living with cancer (cancers not yet cured) and potentially requiring active treatment or ongoing care are expected to be substantial.

This study has some limitations. As is common for all modelling studies, the incidence projections depend on several assumptions, which have been extensively discussed in our previous publication,^2^ and are also described in detail in Supplementary material section 6. Briefly, the age-risk effects were assumed to remain unchanged over time and reflect the general level of cancer risk in the population. For cancers other than lung and prostate cancer, the cohort and period effects of the most recently observed cohort and period were assumed to apply for future unobserved cohorts and periods.^2^ For lung, prostate and breast cancers, the most recent trends in smoking exposure, PSA testing and breast cancer screening participation were assumed to continue. Recent improvements in survival were assumed in the main analyses to continue to 2029 and then remain constant to 2050. These projections do not capture the potential impacts of future changes in treatment effectiveness, recent improvements in cancer control programs, or the introduction of entirely new prevention or treatment paradigms or new screening programs, such as the new targeted lung cancer screening program introduced in July 2025.^17^ Another limitation of this study is that we derived overall UIs for the prevalence estimates based on separate UIs for incidence and survival, and assumed a perfect correlation between incidence and survival. In addition, this study does not account for all non-melanoma skin cancers (NMSC), as NMSC are not notifiable diseases and so are not routinely collected by the population-based cancer registries, with only rare types of NMSC included in the national data.^5^ It should be noted that NMSC are highly prevalent and therefore significantly contribute to health care costs and clinical workload, including dermatology services and surgical demand, and thus separate projections for NMSC would be warranted in the future. Furthermore, this study relies on aggregated information and does not account for individual-level characteristics, and hence masks any disparities in the cancer burden for sub-populations, such as people with socioeconomic disadvantage. Our prevalence projections provide important population-level benchmark data against which to evaluate the impact of cancer control initiatives. Further research is vital to estimate future disparities in cancer prevalence and develop dedicated benchmarks for different priority populations, thereby supporting long-term health care planning.

Our study has many strengths. Firstly, the long-term observed data are known to be of high quality and reflect high quality cancer registration processes across Australia.^5^ Secondly, the study used validated incidence projections (Supplementary Fig. S3) as input data. These models explicitly integrated the effect of selected crucial cancer-related factors, including cigarette smoking exposure for lung cancer, PSA testing rates for prostate cancer, and mammography screening participation rates for breast cancer. For other cancer types, APC models were used, with age, period and cohort effects that might implicitly capture the effects of a diverse factors contributing to cancer incidence at the population level.^2^ Third, the prevalence estimates were independently validated using prevalence estimates reported by the AIHW^5^ (Supplementary Fig. S6 and S7), demonstrating the robustness of our modelling approach. In addition, the methods used in this study may also be applicable in other settings.

Our projections are consistent with those from a recent study that reported long-term cancer prevalence projections for the US.^18^ Notably, we estimated that the majority of people living with or beyond cancer in Australia are long-term survivors, with 69.8% of all survivors diagnosed at least 5 years earlier. A similar pattern was reported in the US, with over 70% of survivors estimated to be at least 5 years, 48% at least 10 years, and 11% at least 25 years from initial diagnosis.^18^ Our study suggests that 6.0% and 7.1% of the total Australian population will be living with or beyond cancer in 2025 and 2050, respectively, with similar increasing trends as projected in the US.^18^ In both studies, the most prevalent groups of survivors are those with a history of breast, prostate, colorectal cancers or melanoma.^18^ However, one difference was that we projected more male than female survivors in Australia, while this is reversed in the US.^18^ This difference is likely due in part to high numbers of survivors of prostate cancer, and the male predominance of most other cancer types in Australia, including high prevalence cancers (colorectal cancer and melanoma). This in turn is due to the consistently higher cancer incidence rates for males than for females (Supplementary Fig. S4). More generally, the difference in sex distribution of cancer survivors between our study and the US study may derive from different health profiles in different population settings, and the differing methods used in these studies (the US study used the PIAMOD method with adjustment factors calculated by comparing the 2018 prevalence estimates from SEER∗Explorer and PIAMOD).^18^

We estimated that in 2050 the greatest numbers of people with a past cancer diagnosis in Australia will be in older age groups (e.g., aged 60 and over), and that over 1 million Australians aged 80 and over will be cancer survivors, comprising 38.8% of all cancer survivors and 41.9% of all Australian people in that age group. Importantly, among individuals diagnosed within the previous 5 years, the largest proportion will be aged 70 to 79, followed by those aged 80+ and those aged 60–69 in 2050. This has very important implications for the health care system, as surveillance is generally recommended during a 5-year period post-diagnosis.^19^ This surveillance will thus predominantly occur among older survivors, who will often have other complex health problems and high incidence of comorbid illness and health risk factors.^20^ Estimates in 2020–2022 suggest Australian men and women aged 65 can expect to live another 20 and 23 years, respectively.^21^ However, around a fifth of all Australians over the age of 65 have a severe or profound disability and around a quarter self-assess their health as fair or poor.^21^ Coronary artery disease, dementia and chronic obstructive pulmonary disease are common coexisting diseases.^21^ Thus, the increases in the number of people living with or beyond cancer for middle and older age groups have major implications for health service planning, as effective treatment and management of multimorbidity requires integrative coordination of health professionals, specialists and services across the health care system.^22^ It should be noted that the approach used to estimate the number of people living beyond cancer (potentially cured) is based on population-level data rather than the clinical definition of cure. Late recurrences could occur after the 95% conditional survival threshold is reached. This is particularly relevant for cancer types characterised by late clinical events, so it is important to acknowledge that the term ‘potentially cured’ does not imply a total absence of clinical risk.

For those not yet classified as potentially cured, people living with cancer are at higher risk of death than those without a prior cancer diagnosis, and are particularly at greater risk of cardiovascular death.^23^ Some survivors, particularly those treated for cancer in childhood or in adolescence, are at risk of serious late onset effects, including the risk of organ failure or a second cancer as a result of previous cancer treatments.^24^ There are significant opportunities to reduce the risk of subsequent disease and premature death through effective management of chronic disease and health risk factors. For example, the recently-reported CHALLENGE study found that supervised exercise can improve disease-free and overall survival, and improve fitness and quality of life for patients treated for early stage colon cancer.^25^ This underscores the potential impact of lifestyle interventions to improve survivorship outcomes.

Current follow up of cancer survivors is generally specialist-led (by the oncologist, haematologist or surgeon) and tends to have a predominant focus on surveillance for possible recurrence or a second cancer, and management of physical effects.^26^ However, it should be noted that there is a lack of evidence to support a survival advantage from routine surveillance for recurrence, for most cancer types.^27^ Survivorship care should focus not just on surveillance for possible recurrence and second cancers, but should also focus on observation for and management of physical and psychosocial issues, health promotion and disease prevention, and management of chronic health conditions.^28^ Notably, the majority of cancer survivors currently experience persisting symptoms, impairment and unmet needs after finishing treatment for cancer.^19^ People may have physical symptoms such as pain and fatigue, psychological issues such as distress, depression and fear of cancer recurrence, and practical issues such as difficulty returning to work and loss of income.^19^ Functional impairment and reduced quality of life may persist for years.^19^ Managing the very large numbers of survivors in the future will thus require different models of care which appear at least as effective as specialist-led care, including models where follow up is led by the survivor’s primary care provider or general practitioner (GP), shared between oncology providers and the patient’s GP, and nurse-led models.^26^ These models may be more sustainable from a resource and economic perspective. Supporting survivors to self-manage, and use of remote surveillance may support more sustainable survivorship care.^26^^,^^29^

Providing timely and appropriate care to the large and growing number of survivors remains a challenge. Significant expectations are being placed on the development of technology, including artificial intelligence, that may help identify those at risk of persisting or late onset problems. Long-term health consequences may be reduced in the future through more effective treatments with reduced toxicity and lower side effect profiles (as has been seen with more modern treatments for childhood cancers),^30^ and prompt identification and management of concerns during treatment. To help counsel patients and plan health services, it is vital to address knowledge limitations on long-term outcomes for diverse groups of cancer survivors, including those treated with newer therapies. For example, immunotherapy has become a standard treatment for many cancers over the past 10–15 years, yet there are very little data regarding long-term consequences and impacts from these therapies. In addition, there are limited data regarding those living *with*, rather than *beyond* a cancer diagnosis, even though more effective treatments mean an increasing number of people are living longer with treatable but not curable cancers.^31^ Estimating the number of people living with advanced or metastatic cancers is essential for health service planning. In the current report we have provided first estimates of those living with, rather than living beyond cancer (potentially cured). Many people living with advanced disease have significant unmet needs and undergo ongoing treatments.^31^ Estimating the number of people living with advanced stage cancer in Australia will be a focus of our future work, to fully understand the healthcare system required to adequately support those with advanced and metastatic disease.

### Conclusions

This study provides timely estimates of long-term projections of cancer prevalence in Australia to 2050, by assuming constant age-risk effects, taking into account past and recent trends in smoking exposure, PSA testing and breast cancer screening participation, and assuming that improvements in survival will plateau after 2029. Although the aggregate projections do not capture important disparities between population subgroups, with future work required to estimate cancer prevalence for priority populations including Aboriginal and Torres Strait Islander peoples, rural populations, or socioeconomically disadvantaged groups, our findings do provide whole-of-population estimates which are a critical first step for forward planning. Our findings suggest that the overall numbers of cancer survivors living in Australia in 2050 are expected to increase substantially, with approximately half of these requiring active treatment and ongoing cancer care. This highlights the pressing challenge for the health system to provide care to the large and growing number of cancer survivors. In particular, the projected increases in the number of cancer survivors for the middle and older age groups (e.g., the number of cancer survivors aged 80 and above is expected to more than double over 2025 to 2050), where multimorbidity is common, stress the need for a more integrated health system to adequately manage the large cancer burden over the coming years.

## Contributors

KC and MJ conceived the study, building on past work led by QL, KC, JS and EF. QL conducted the literature search, designed the methods and conducted the formal analysis. KC, MJ and JS advised on methods. QL, KC and MJ interpreted the results and drafted the original manuscript. QL and MD have directly accessed and verified underlying data. QL, DPS, MD, EF, CK, JS, MJ and KC have direct access to the raw data, and contributed to the interpretation of the results, reviewing and editing of manuscript, and approved the final manuscript. KC, MJ and JS contributed equally as co-senior authors. QL, KC, MJ, JS and MD made the final decision to submit for publication.

## Data sharing statement

The tabulated data for cancer incidence, survival and prevalence are available at https://www.aihw.gov.au/.

## Declaration of interests

EF declares that she received funding support from Cancer Institute NSW for grant-funded research projects. EF received funding from University of Melbourne for travel from Sydney to Melbourne for grant-related projects. All payments are made to the University of Sydney.

JS declares that she holds a Cancer Institute NSW Career Development Fellowship (Jun 2023 to current). All payments are made to the University of Sydney and Cancer Council NSW.

KC holds a Fellowship from the National Health and Medical Research Council of Australia (NHMRC). All payments are made to her institution. She is co-PI of an investigator-initiated trial of HPV screening in Australia (‘Compass’), which is conducted by the Australian Centre for Prevention of Cervical Cancer (ACPCC), a government-funded health promotion charity. The ACPCC has previously received equipment and a funding contribution for the Compass trial from the Australian government and Roche Molecular Systems USA. She is also co-PI on a major implementation program “Elimination Partnership In Cervical Cancer” which receives support from the Australian government, the Minderoo Foundation and equipment donations from Cepheid Inc and Microbix.

The remaining authors declare that they have no conflicts of interest.
