# Supplementary material for: Long-term projections of cancer prevalence in Australia to 2050: a population-based statistical modelling study
Source: Lancet Reg Health West Pac. 2026 Aug 17;73:101946. doi: 10.1016/j.lanwpc.2026.101946 (PMC13540674; doi:10.1016/j.lanwpc.2026.101946)
Supplement: Supplementary file 1 [file mmc1.pdf]

Supplementary material accompanying the article:

Long-term projections of cancer prevalence in Australia to 2050: a population-based statistical modelling study

Citation: Luo Q, Smith DP, David M, Feletto E, Kahan C, Steinberg J*, Jefford M*, Canfell K*. (2026). Long-term projections of cancer prevalence in Australia to 2050: a population-based statistical modelling study. Lancet Reg Health West Pac. https://doi.org/10.1016/j.lanwpc.2026.101946

Table of Contents

1. Overview of data sources and study approach 1
1.1. Australian population data 2
1.2. Data on smoking exposure in Australia 2
1.3. Data on breast cancer screening participation rates 2
1.4. Data on prostate-specific antigen testing 3
1.5. Cancer incidence data 3
1.6. Cancer survival data 4
1.7. Cancer prevalence data 4
2. Definition of limited duration cancer prevalence 5
3. Statistical analysis 5
3.1. Cancer incidence projections 5
Model assumptions 7
Model validation 8
3.2. Cancer survival estimates 9
3.3. Method to estimate cancer prevalence 9
3.4. Estimating the uncertainty intervals 9
3.5. Validation of prevalence projections 14
4. Estimating the number of people living beyond cancer (potentially cured) 16
5. Sensitivity analyses 18
6. Limitations of projection methods 18
7. Additional results 22
8. References 47

List of Tables

Table S1. ICD-10 codes for cancer types included in this study 4
Table S2. Final statistical projection methods used for incidence rate projections for selected cancer types and all cancers combined 6
Table S3. Model assumptions for incidence projections 7
Table S4. Estimated cancer prevalence by sex and time since diagnosis, Australia, 2050 22
Table S5. Estimated cancer prevalence by sex and prevalence duration in Australia, 2025 to 2050 25
Table S6. Estimated cancer prevalence by sex, age group and prevalence duration in Australia, 2025 and 2050 27
Table S7. Estimated cancer prevalence by age groups and prevalence duration in Australia, 2025 to 2050 29
Table S8. Estimated number of people living with cancer (cancers not yet cured) and living beyond cancer (potentially cured) in Australia, 2025 to 2050 32

List of Figures

Figure S1. Data sources[1-7] and study approach[8-10] 1
Figure S2. Illustration of concept of cancer prevalence* 5
Figure S3. Validation of 10-year projections for age standardised incidence rates for all cancers combined and individual cancer types in Australia using observed data from 1982 to 2009 projected to 2019, compared to observed data for 2010-2019 11
Figure S4. Observed and predicted age standardised incidence rates for all cancers combined and individual cancer types in Australia to 2050 12
Figure S5. Historical (1986-2019) and projected (2020-2050) cancer-specific survival in Australia 13
Figure S6. Validation of prevalence projections: Comparison of the predicted prevalence in this study with prevalence reported by the AIHW, 2000 – 2020 14
Figure S7. Validation of prevalence projections: Comparison of the predicted age-specific 10-year prevalence in this study with prevalence reported by the AIHW, 2000 – 2020 15
Figure S8. Five-year conditional relative survival by years after initial diagnosis using the period methods, Australia 2016-2020 16
Figure S9. Change in rank of cancer types sorted by decreasing cancer prevalence over the period 2025-2050, Australia, separately for all persons and by sex 35
Figure S10. Estimated cancer prevalence (95% uncertainty interval) by sex in Australia, 2025 – 2050 37
Figure S11. Estimated cancer prevalence by age group in Australia, 2025 – 2050 40
Figure S12. People living with cancer (cancers not yet cured) and living beyond cancer (potentially cured) ranked by estimated 30-year cancer prevalence in 2050, separately for males and females 43
Figure S13. Results of sensitivity analyses for cancer prevalence projections in 2050, Australia 45
Figure S14. Results of sensitivity analyses for cancer prevalence projections of people living beyond cancer (potentially cured) and people living with cancer (cancers not yet cured) in 2050, Australia 46

1. Overview of data sources and study approach

Figure S1. Data sources[1-7] and study approach[8-10]

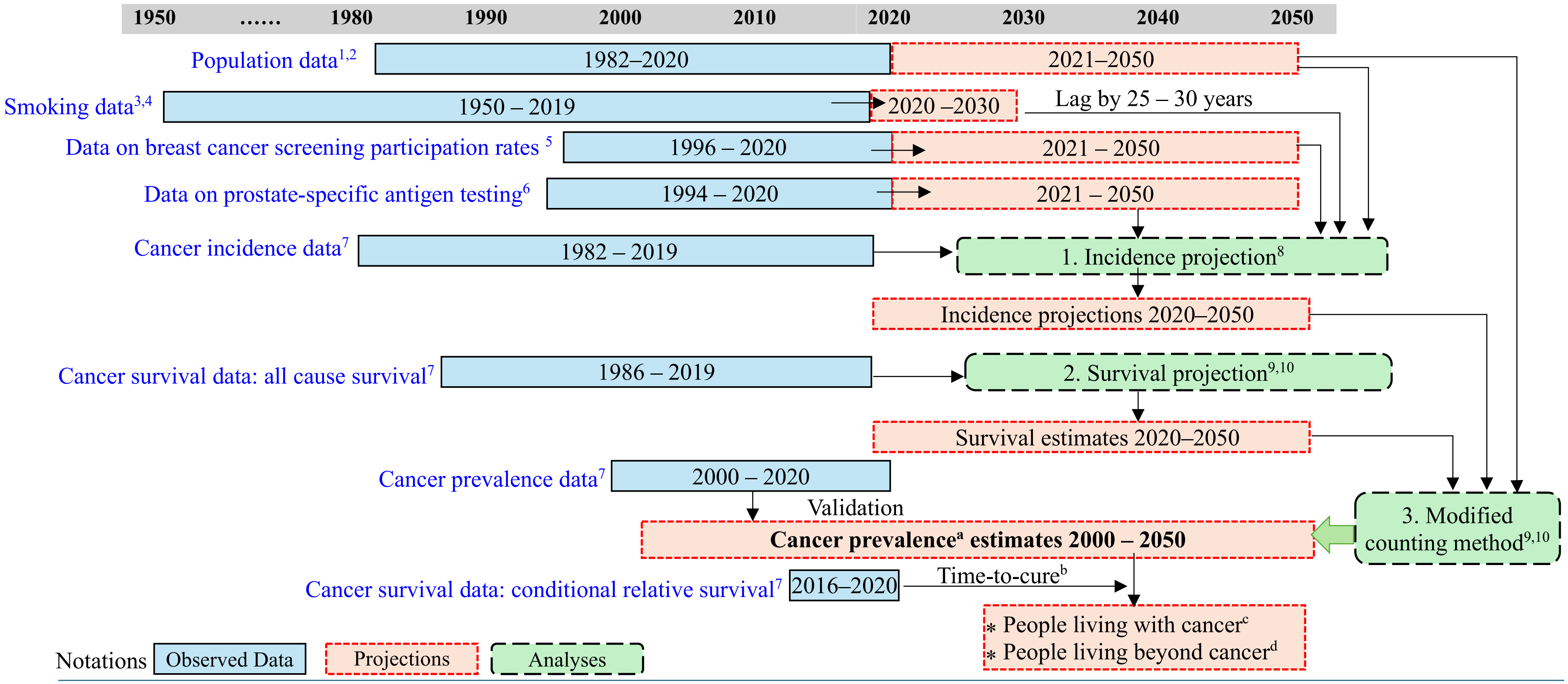

a. In this study, annual prevalence was defined as the number of individuals alive in a calendar year who were previously diagnosed with cancer within a specified duration of follow-up after initial diagnosis. b. Time-to-cure was defined as the number of years after initial cancer diagnosis for which the 5-year conditional survival estimate exceeds 95%, or exceeds 90% and reaches a plateau. c. People living with cancer (cancers not yet cured) refers to people with a past cancer diagnosis, requiring initial treatment or ongoing care for cancer in the year. d. People living beyond cancer (potentially cured) refers to people with a past cancer diagnosis whose time since initial diagnosis reached or exceeded the estimated "time-to-cure".

1.1. Australian population data

We obtained Australian population data by sex, 5-year age group and calendar year (1950 to 2050) from the Australian Historical Population Statistics and Population Projections (Series B, based on medium population growth) produced by the Australian Bureau of Statistics.[1,2]

1.2. Data on smoking exposure in Australia

Data on cigarette smoking exposure were obtained from the International Smoking Statistics (ISS) Web Edition,[4] which provides data from several different surveys and information on annual tobacco sales, and the National Drug Strategy Household Survey (NDSHS) for 2007-2019.[3] The NDSHS data in 2022-23 were not included, as these data have been collected during the COVID-19 pandemic and may not be directly comparable to previous data.[11] Detailed descriptions of the data sources for cigarette smoking exposure are provided in our previous study of lung cancer mortality in Australia.[12] In brief, sex-age-period-specific smoking prevalence and cigarette tar exposure per capita (smoothed using a three-year moving average) in Australia were re-constructed backwards to 1950 and forwards to 2030 using log-linear regression models to accommodate a lag of 20-30 years.[12]

1.3. Data on breast cancer screening participation rates

Australia currently has national cancer screening programs for female breast, cervical, colorectal and lung cancer. The BreastScreen Australia services for breast cancer screening and the National Cervical Screening Program (NCSP) for cervical cancer were introduced in the early 1990s, and the Australian National Bowel Cancer Screening Program (NBCSP) was introduced in 2006 under a phased implementation, with full roll-out to all eligible Australians only achieved in 2020.[13] Most recently, in July 2025, Australia launched the National Lung Cancer Screening Program (NLCSP), targeting asymptomatic individuals aged 50-70 years with a smoking history of at least 30 pack-years within the last 10 years.[14] Available population-wide screening participation data could not be incorporated into the statistical projection models for colorectal and lung cancer, as the screening participation data were unavailable for the time period required for the statistical modelling. Detailed projections for colorectal cancer based on microsimulation modelling have been published elsewhere.[15] For cervical cancer, as a result of the recognition of the association between cervical cancer and persistent infection with the human papillomavirus (HPV), to further reduce cervical cancer incidence Australia introduced the National HPV Vaccination Program in April 2007 targeting people aged 12-13 years.[16] A recent simulation study has shown that cervical cancer could be eliminated as a public health concern if high coverage in both the HPV vaccination program

and participation in the concurrent national cervical screening program are maintained with the vaccination program most likely impacting cervical cancer rates after 2044.[17] Also, while a cervical cancer screening program was first introduced in Australia in the early 1990s, a revised protocol using a new screening technology was introduced in 2017.[17] Due to all of these changes we were not able to incorporate data on HPV vaccination and participation rates in the updated screening program into the statistical modelling for this study.[8]

National tabulated data on participation rates by age group in the breast cancer screening program from 1996 to 2020 were obtained from the Australian Institute of Health and Welfare (AIHW).[5] Data were smoothed using a three-year moving average. The participation rate over 2021-2050 was projected using age-period-cohort (APC) models. Screening data from BreastScreen Australia are considered to be high quality, with the majority of screening mammograms performed in Australia occurring through BreastScreen Australia.

1.4. Data on prostate-specific antigen testing

Prostate-specific antigen (PSA) testing is not considered to be a population-level screening test. However, the PSA testing rate is a strong predictor for the prostate cancer incidence rate.[8] National tabulated data on the PSA testing rates by age group between 1994 and 2020 were obtained from the Medicare Benefits Schedule (MBS) data (MBS item number 66655),[6] and log-linear regression was applied to data for 1994-2020 by age and year to estimate the PSA testing rates for 2021-2050.

1.5. Cancer incidence data

Data on new diagnoses for 24 individual cancer types, the group of remaining cancers referred to here as 'other cancers' and for all cancers combined by sex, 5-year age group and calendar year for 1982-2019 were obtained from the AIHW (International Classification of Diseases 10th Revision [ICD-10]).[7] Selected cancer types and codes are provided in Table S1.

Table S1. ICD-10 codes for cancer types included in this study

Cancer groups	ICD 10-code
All cancers combined*	C00-97, D45-46, D47.1, D47.3-5
Bladder	C67
Brain	C71
Breast	C50
Cervix	C53
Colorectum	C18-C20
Gallbladder and bile duct	C23-C24
Kidney	C64
Larynx	C32
Leukaemia	C91-C95
Lip, oral cavity and pharynx	C00-C14, C30-C31
Liver	C22
Lung	C33-C34
Melanoma	C43
Multiple myeloma	C90.0
Non-Hodgkin lymphoma	C82-C85
Oesophagus	C15
Ovarian/tubal serous cancer	C56 and C57.0, C57.8 (with histologies 8441, 8460, 8461)
Pancreas	C25
Prostate	C61
Stomach	C16
Testis	C62
Thyroid	C73
Unknown primary site	C77–C80, C97
Uterus	C54-C55
Other cancers	All cancers excluding the 24 selected individual cancer types.

* Does not account for all non-melanoma skin cancer (NMSC), as in most states and territories NMSCs are not notifiable and are not collected by population-based cancer registries, and data for NMSCs (rare types) is available from 2001 onwards.

1.6. Cancer survival data

To estimate cancer prevalence, we obtained stratified data on observed all cause survival by sex, 5-year and 20-year age groups, year after initial diagnosis (1 to 30 years for overall and 1 to 10 years for age-specific survival) and year of diagnosis for 1986-2019 from the AIHW.[7]

To estimate the number of individuals living beyond cancer (potentially cured), 5-year conditional relative survival (compared to the general population) in 2016–2020 using the period method was obtained from the AIHW.[7]

1.7. Cancer prevalence data

The 1-, 5-, 10-, and 33 to 39-year cancer prevalence by sex and 10-year age groups were obtained from the AIHW.[7]

2. Definition of limited duration cancer prevalence

Limited-duration annual cancer prevalence is the number of people alive at any time during a calendar year who are within a specified number of years from initial cancer diagnosis, such as those diagnosed within the 5 or 30 years preceding that calendar year (Figure S2). For example, as illustrated in Figure S2, persons C, F and H are counted in 5-year prevalence in 2025, and persons B, C, D, F and H are counted in 30-year prevalence in 2025.

Figure S2. Illustration of concept of cancer prevalence*

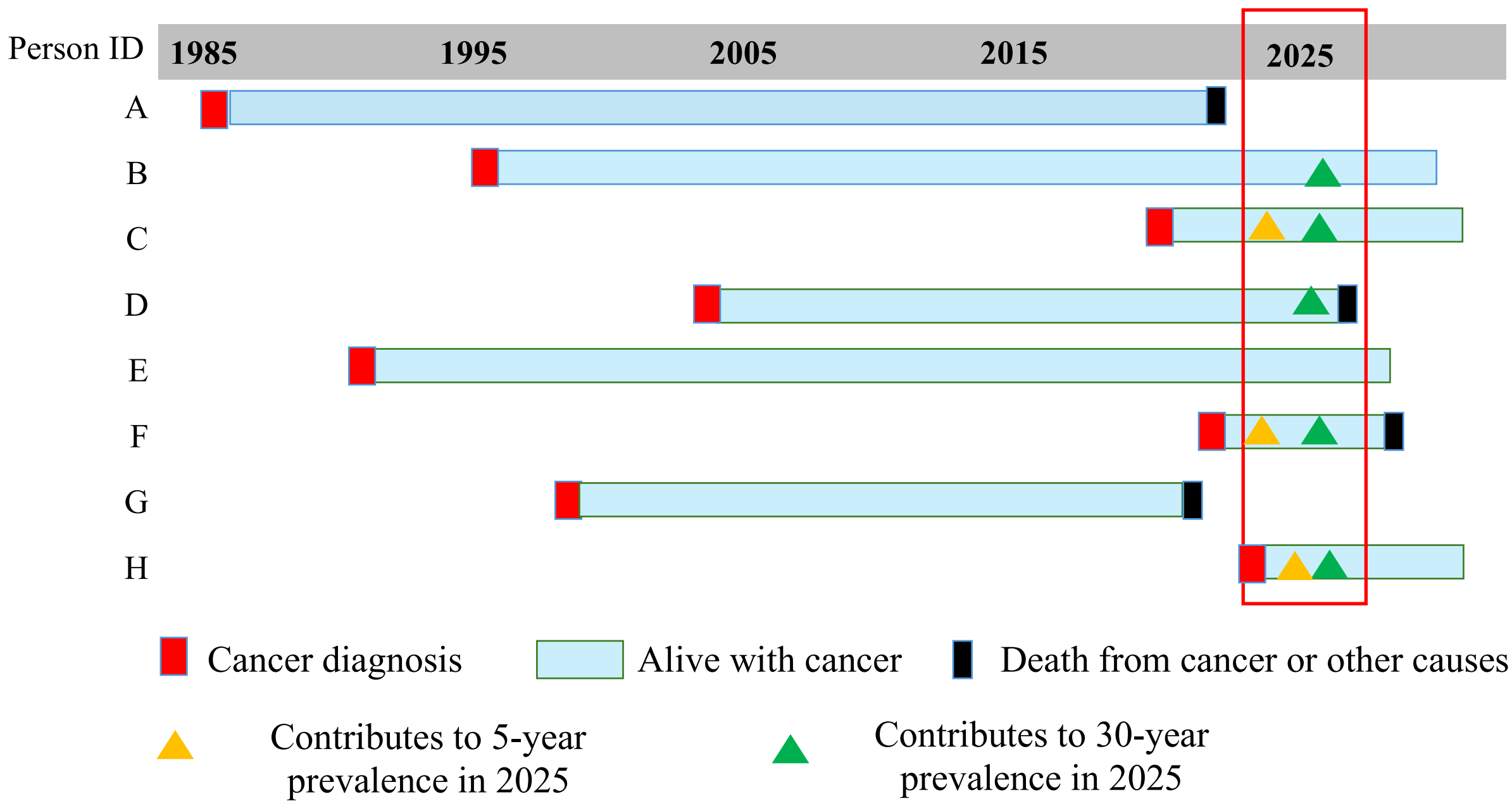

* In this study, annual prevalence was defined as the number of individuals alive in a calendar year with a past cancer diagnosis within a specified duration of follow-up after initial diagnosis.

3. Statistical analysis

We previously developed and validated a modified counting method to estimate prevalence based on the tabulated incidence and survival data.[10] A more detailed description of the modelling methods is available elsewhere.[9,10] Briefly, for each cancer type by sex, we first developed and validated statistical models to project the number of new diagnoses to 2050. We then extrapolated the observed sex-age-specific survival rates forward to 2029, to account for the impact of improvement in treatment, and then assumed survival will remain constant to 2050. We then estimated 1-, 5- and 30-year prevalence as a function of incidence and survival, as described in the following.[9,10]

3.1. Cancer incidence projections

A more detailed description of our previously developed and validated incidence projection methods is available elsewhere.[8] Briefly, basic modelling approaches are used: (1)

Generalised linear model (GLM) with Poisson distribution (models were fitted using the *glm* command in Stata), (2) APC models include age, period and cohort components within the framework of a GLM.[18] APC models were fitted using the *apcspline* command in Stata with natural cubic splines for smoothing.[19,20] These methods can explicitly incorporate additional variables on factors related to cancer incidence rates.[8]

APC models incorporating cigarette smoking exposure were used to project the incidence rates for lung cancer.[8] The cohort effect is considered to be a reflection of many cohort-specific smoking characteristics and the period effect may reflect other relevant factors such as environmental and occupational factors, and ad hoc lung cancer screening with low dose computerised tomography.[8] An APC model incorporating the participation rate in the breast cancer screening program was used to project the incidence rate for female breast cancer.[8] For the prostate cancer incidence rate, we used a GLM with a Poisson distribution including age, period and age-specific PSA testing rates.[8] For colorectal cancer incidence we used separate age-stratified APC models for ages <50 years and ages 50 or more years to account for the possible impact of opportunistic and organised screening.[8] Standard APC models were used to project incidence rates for all remaining 20 cancer types. For some cancers, if the period effect or cohort effect was not statistically significant (i.e. p>0.05) in the full APC model, an age-cohort (AC) or age-period (AP) model was selected as the final projection model (Table S2).

Table S2. Final statistical projection methods used for incidence rate projections for selected cancer types and all cancers combined

**Cancer group**	**Statistical projection methods and model assumptions**
Breast	APC model with screening participation rate as a covariate (lagged 1 year).
Lung	APC model with cigarette smoking exposure as a covariate (lagged 28 years for males and 30 years for females).
Prostate	GLM includes age, period and PSA testing rate as a covariate (lagged 1 year).
Colorectum, Ovarian/ tubal serous cancer	Age-stratified APC model for age <50 years and age 50 or more
Brain, liver, multiple myeloma	AC model
Oesophagus	AP model for males, AC model for females
Pancreas	AP model for males, APC model for females
Remaining cancer groups	APC models.
All cancers combined	Summing the number of new cases for 24 individual cancer types and the remaining cancer types as an 'other' group.

GLM: generalised linear model; AC: age-cohort; AP: age-period; APC: age-period-cohort; PSA: prostate-specific antigen.

Model assumptions

The assumptions underlying the projection models are listed in Table S3. To project cancer incidence rates beyond the observed period, the age effect was assumed to remain unchanged over time and reflects the general level of cancer risk in the population.[8] For lung cancer incidence, we used cohort-specific cigarette tar exposure to predict the future cohort effects.[8] For prostate cancer incidence, we used period-specific PSA testing rates to predict the future period effects.[8] For other cancers, the nonlinear period effects beyond the last observed period are assumed to be the same as those in the last observed period. Although birth cohort effects carry through into the future as cohorts age, future cohorts are assumed to have the same effect as the last observed cohort. The default setting for the damping factor (equal to 0.92) was used, so that we reduced the drift by 8% for each year following the last observation.[8,19]

Table S3. Model assumptions for incidence projections

Parameter	Assumptions
**Common assumptions that apply to all cancer types/groups, unless otherwise specified below**	
Age effect	The age effect remained unchanged over time and reflects the general level of cancer risk in the population.
Period effect	The nonlinear period effects beyond the last observed period are assumed to be the same as those in the last observed period.
Cohort effect	Although birth cohort effects carry through into the future as cohorts age, future cohorts are assumed to have the same effect as the last observed cohort. Due to the nonidentifiable issue of the APC model, the cohort effects from age stratified APC models may resulted in different cohort effects. Due to the non-identifiability of the linear components of the age, period and cohort parameter estimates, there is no way to distinguish the period effect and the cohort effect.
Population	Population denominators are projections available from the Australian Bureau of Statistics, based on medium population growth.
Damping factor	Assume that the drift is attenuated over time, we applied a damping factor to the drift (i.e., the linear trend over time) when extrapolating to the future. We used the default setting damping factor equal to 0.92 so that we reduce the drift by 8% for each year following the last observation.
**Specific assumptions for breast cancer**	
Coefficient for breast cancer screening	The model assumes that the breast cancer screening participation rate has a linear relationship with the log of the number of breast cancer diagnoses, so that each unit change in the breast cancer screening participation rate produces a constant multiplicative effect on breast cancer incidence rate.
**Specific assumptions for lung cancer**	

Cohort effect	Sex-age-period-specific smoking exposure in 2020-2022 were predicted using log-linear models by assuming the recent trends will continue and lagged 28 for males and 30 years for females.<br>We used cohort-specific cigarette tar exposure to predict the future cohort effects.
Coefficient for smoking exposure	The model assumes that smoking exposure has a linear relationship with the log of the number of lung cancer diagnoses, so that each unit change in the smoking exposure produces a constant multiplicative effect on lung cancer incidence rate.
**Specific assumptions for prostate cancer**	
Period effect for prostate cancer	PSA testing rates for 2021-2050 were predicted using log-linear regression, by assuming the recent trends will continue.<br>We used period-specific PSA testing rates to predict the future period effects.
Coefficient for PSA testing rate	The model assumes that PSA testing rate has a linear relationship with the log of the number of prostate cancer diagnoses, so that each unit change in PSA testing rate produces a constant multiplicative effect on prostate cancer incidence rate.

Model validation

The projection models were validated by fitting the model using data up to 2009 and then compared the projected rates with the actual observed rates in 2010-2019.[8] Validation of 10-year projections of incidence rates for the 24 individual cancer types and all cancers combined showed that the projected rates were in good agreement with the observed rates, and the uncertainty intervals of the projected rates generally captured the observed rates, with low absolute differences (median [range] 0.3 [0.0-8.6] cases per 100 000 population (Figure S3). Projected age-standardised incidence rates by sex are shown in Figure S4.

The validation by withholding recent observed data is essential to provide an objective measure of how well the method captures the real-world cancer burden. As discussed above, we acknowledge that our models may not fully capture the impact of any new cancer control initiatives that may be introduced during the projection period. However, our validation exercise suggests that our projection method reasonably captured the impact of more recent cancer control initiatives such as the colorectal cancer screening program rolled out in 2006 and the increases in the tobacco excise since the 2010s. This is likely due to a lag between the implementation of cancer control interventions and the emergence of measurable changes in cancer outcomes at population-level, as the benefits of cancer control initiatives tend to accrue gradually over time after program initiation, and historical cancer data often reflect these relatively monotonic trends. Our validation assessment is considered to be the best available way of assessing the accuracy of the long-term projections in this study.

3.2. Cancer survival estimates

We extrapolated the observed sex-age-specific survival rates forward to 2029, to account for the impact of improved treatment, and then assumed that survival remains constant to 2050. Survival estimates for 2020 to 2029 were projected using linear extrapolation of log-transformed survival from the two most recent observed 5-year periods, using the Stata command *'ipolate'*.[18] For example, if s_0 and s_1 were the survival in the two most recent periods with mid-point years x_0 and x_1, respectively, survival s for a future period with midpoint year x was obtained as (using the notation adapted from Kang et al. 2025[9])

$$\log(s) = \frac{\log(s_1)-\log(s_0)}{x_1-x_0}(x - x_1) + \log(s_1).$$

Analogous to analyses of the observed data, extrapolations were completed separately by cancer type, sex, and age group at diagnosis[9] (Figure S5).

3.3. Method to estimate cancer prevalence

We then estimated 1-, 5- and 30-year prevalence as a function of incidence and survival, by averaging the number of people who were previously diagnosed with cancer and remain alive over two intervals and assuming that people within a 1-year interval contribute to cancer prevalence equally, as described in the following.[9,10]

Specifically, for each cohort of new cancer cases in year *j* at age *a*, we estimated the numbers of these individuals expected to be alive at *k* years after the initial diagnosis in calendar year *p=j+k* at attained age *b=a+k* for a given cancer type/group (using the notation adapted from Luo et al. 2024[10] and Kang et al. 2025[9]):

$$Survivors_{pbk} = New\ cases_{ja} * \left(Observed\ survival_{(k-1)a} + Observed\ survival_{ka}\right)/2$$

This calculation was done separately for males and females, different age groups, and for *k* of 1 to *K* years after diagnosis (e.g. k=1, 2, 3, … 30 for 30-year prevalence).

K-year prevalence (for *K* of 1, 5 and 30 years) in calendar year *p* was then calculated by aggregating the number of all persons previously diagnosed with cancer, alive and within $k \leq K$ years after diagnosis, with separate calculations by sex and different age groups as required.

3.4. Estimating the uncertainty intervals

Prevalence was calculated as a function of incidence and survival. Both incidence and survival estimates had uncertainty intervals (UIs) estimated separately and independently without resampling the data. The UIs for cancer incidence were defined as the confidence intervals of the estimates based on the variances of the parameters in the model.[8] The UIs for

the observed survival were estimated using the Greenwood formula, which calculates the variance of the Kaplan-Meier survival function and then applies this variance to derive pointwise confidence limits for each time point.[7,21] The UIs for cancer prevalence were then calculated based on the UIs for the number of new cases and survival rates. For example, the lower bound of the UI for prevalence was calculated using the lower bound of the UI for the number of new cases and the lower bound of the UI for the survival estimates. This assumes a perfect correlation between incidence and survival. To the best of our knowledge, there is not sufficient data available to model the degree of interdependence between incidence and survival for the purpose of our study, and thus we deemed that Monte Carlo sampling from the incidence and survival distributions to estimate the UIs for prevalence would not be practical. We therefore estimated UIs for prevalence by multiplying the corresponding bounds for the UIs for incidence and survival (i.e. the lowest/highest incidence bound is associated with the lowest/highest survival bound), which produces wider UIs for prevalence than if a smaller correlation is assumed. Thus, the UI estimates for prevalence in this study are considered to be conservative.

Figure S3. Validation of 10-year projections for age standardised incidence rates for all cancers combined and individual cancer types in Australia using observed data from 1982 to 2009 projected to 2019, compared to observed data for 2010-2019

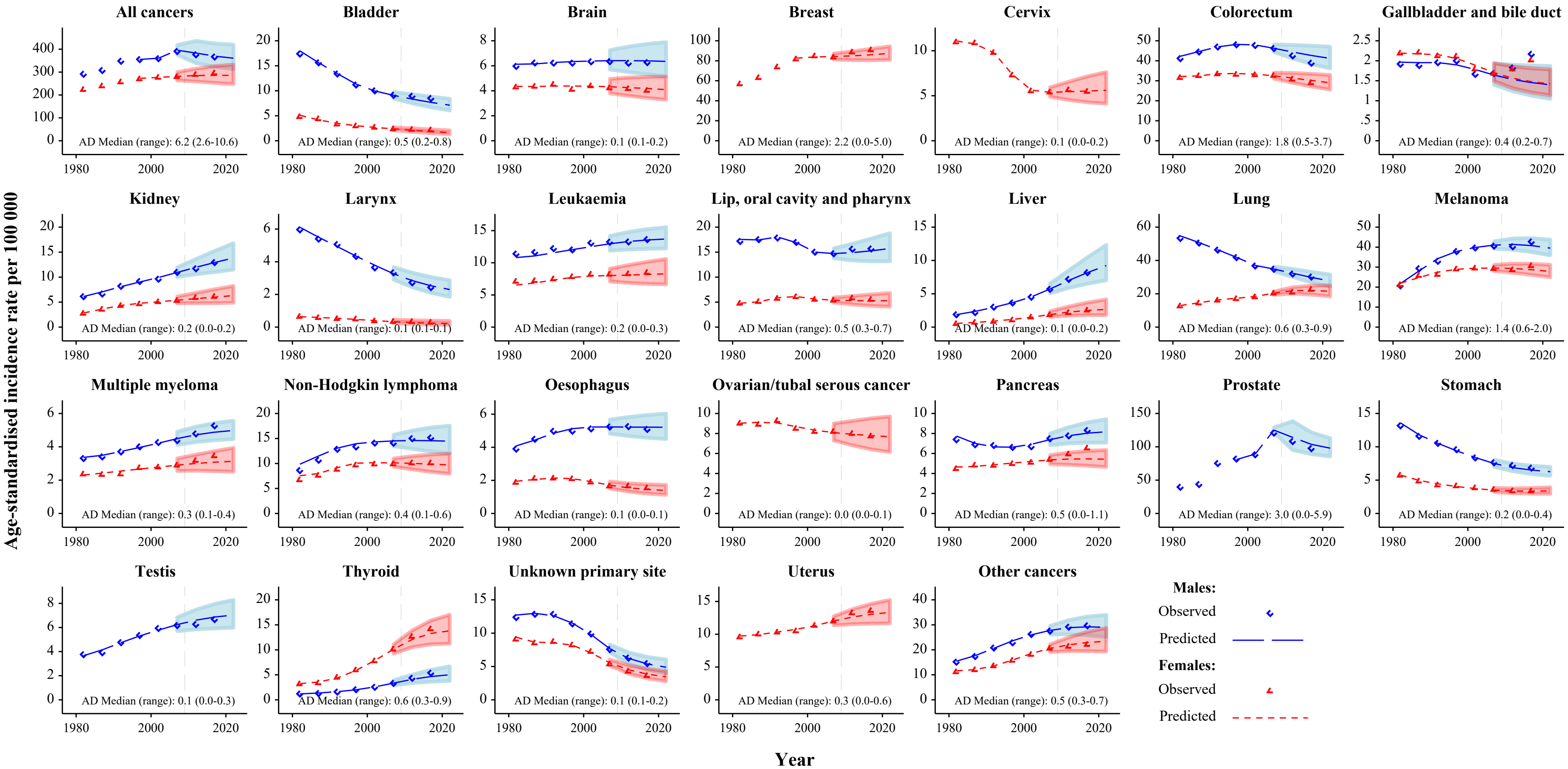

All rates are age-standardised to the Segi World standard population. Absolute difference between projected and observed rates per 100 000 population in 2010-2019 for males. median 0.28 range 0 – 6.5, for females: median 0.24 range 0 – 10.5. Shaded areas represent the 95% uncertainty intervals.

Figure S4. Observed and predicted age standardised incidence rates for all cancers combined and individual cancer types in Australia to 2050

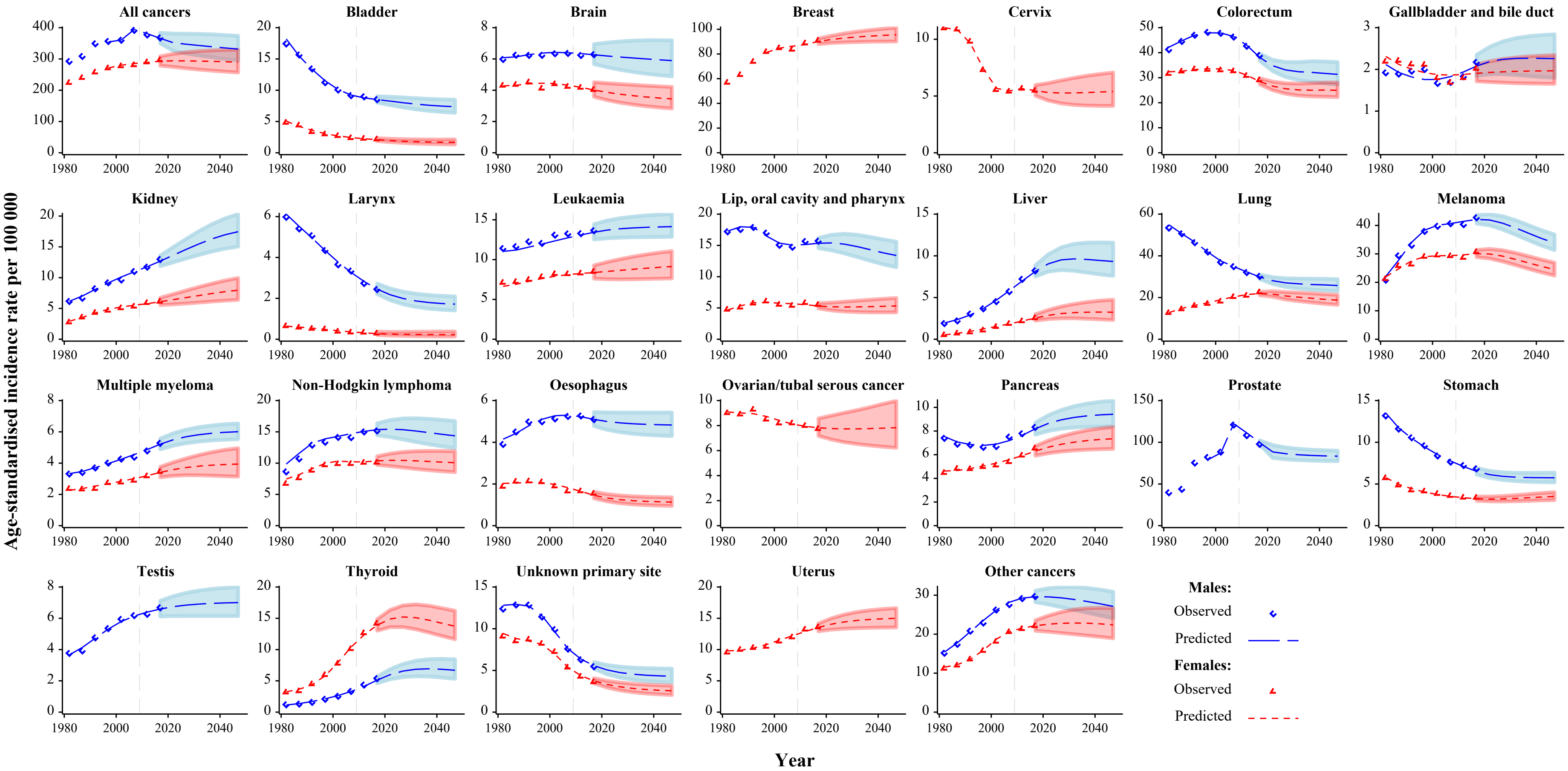

All rates are age-standardised to the Segi World standard population. Shaded areas represent the 95% uncertainty intervals.
To project cancer incidence rates beyond the observed period, the age effect was assumed to remain unchanged over time and reflects the general level of cancer risk in the population. For lung cancer incidence, we used cohort-specific cigarette tar exposure to predict the future cohort effects. For prostate cancer incidence, we used period-specific PSA testing rates to predict the future period effects. For other cancers, the nonlinear period effects beyond the last observed period are assumed to be the same as those in the last observed period. Although birth cohort effects carry through into the future as cohorts age, future cohorts are assumed to have the same effect as the last observed cohort. The default setting for the damping factor (equal to 0.92) was used, so that we reduced the drift by 8% for each year following the last observation.

Figure S5. Historical (1986-2019) and projected (2020-2050) cancer-specific survival in Australia

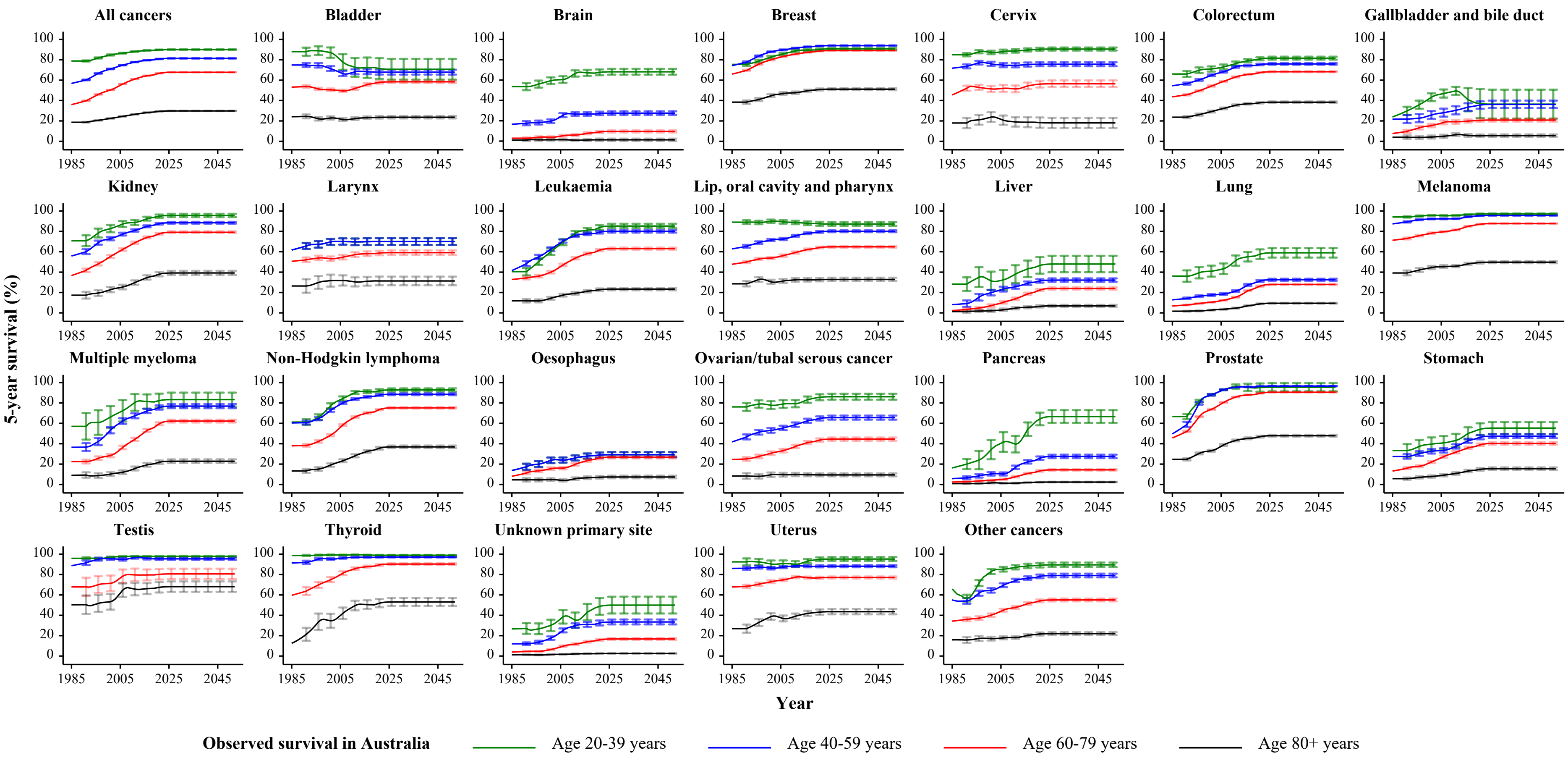

We extrapolated the observed sex-age-specific survival rates forward to 2029, to account for the impact of improved treatment, and then assumed that survival remains constant to 2050**.**

3.5. Validation of prevalence projections

Figure S6. Validation of prevalence projections: Comparison of the predicted prevalence in this study with prevalence reported by the AIHW, 2000 – 2020

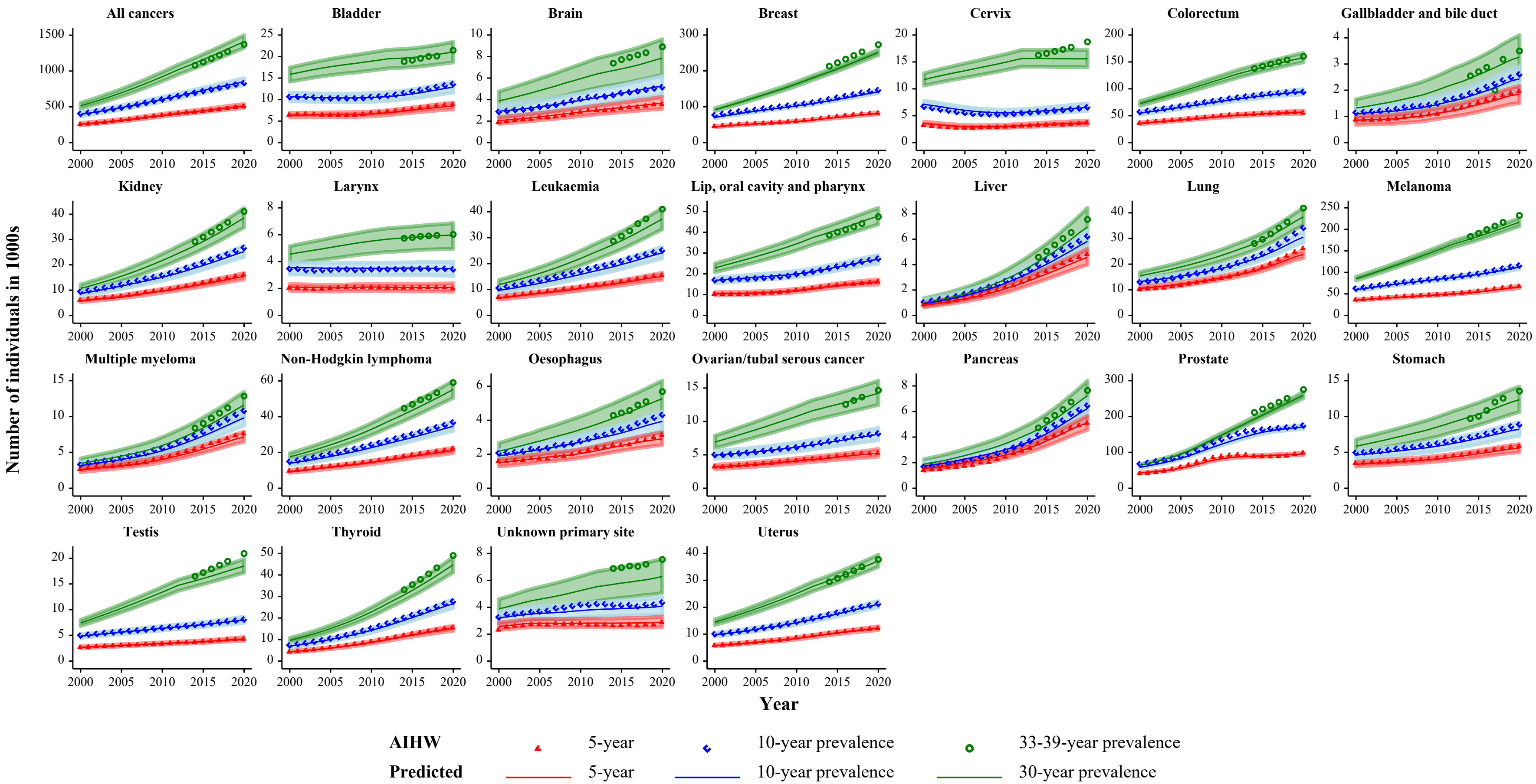

AIHW: the Australian Institute of Health and Welfare, with national 33-39 year prevalence estimates were only available in 2014-2018 and 2020.
Shaded areas represent the 95% uncertainty intervals (UIs) for prevalence estimates. The UIs for the estimated prevalence were estimated based on the UIs of the number of new cases and survival rates.

Figure S7. Validation of prevalence projections: Comparison of the predicted age-specific 10-year prevalence in this study with prevalence reported by the AIHW, 2000 – 2020

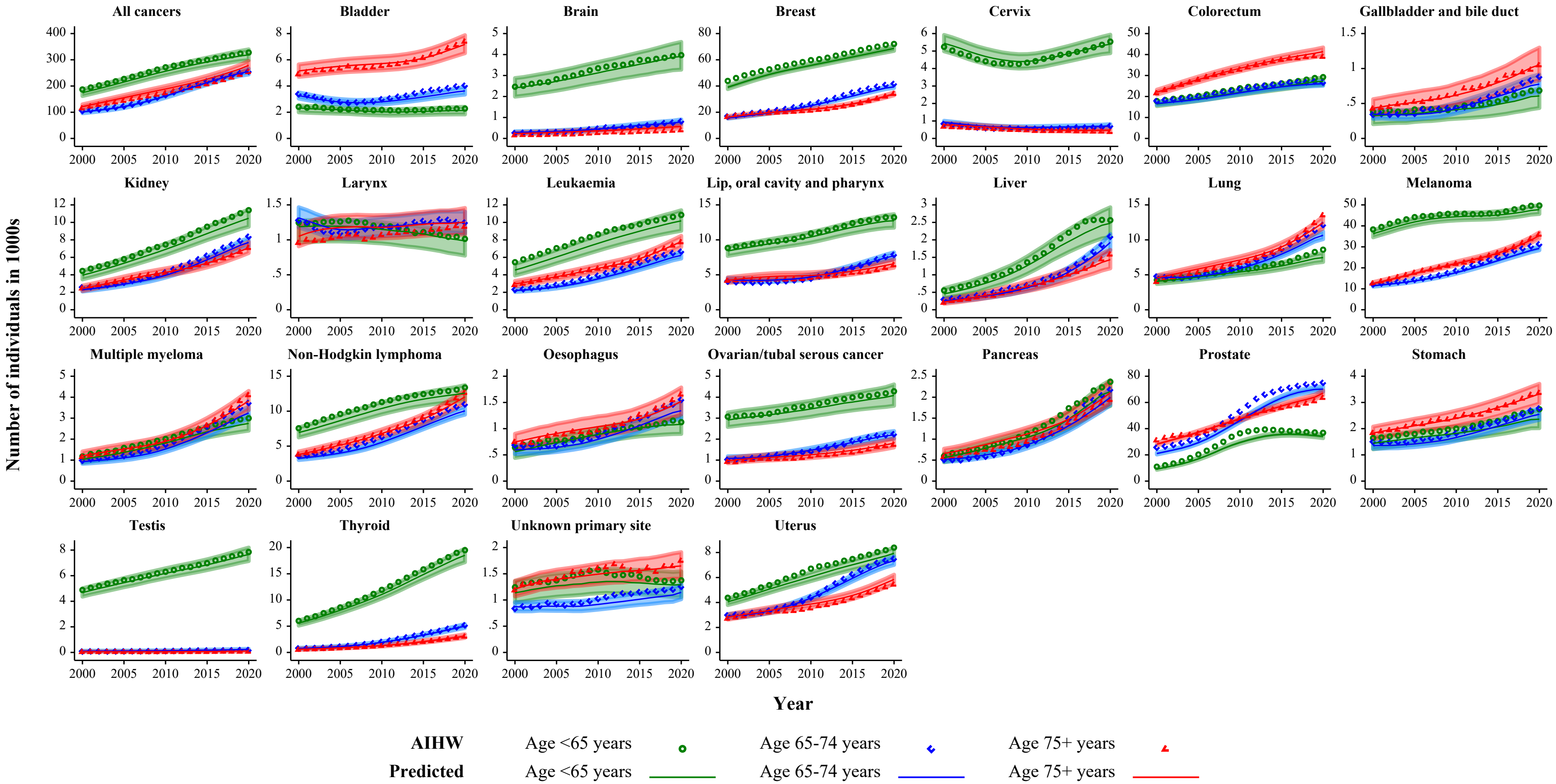

AIHW: Australian Institute of Health and Welfare**.**

Shaded areas represent the 95% uncertainty intervals (UIs) for prevalence estimates. The UIs for the estimated prevalence were estimated based on the UIs of the number of new cases and survival rates.

4. Estimating the number of people living beyond cancer (potentially cured)

"Time-to-cure" was defined as the number of years after initial cancer diagnosis for which the 5-year conditional survival estimate exceeds 95%, or exceeds 90% and reaches a plateau, in comparison with the general population.

Figure S8. Five-year conditional relative survival by years after initial diagnosis using the period methods, Australia 2016-2020

A. Males

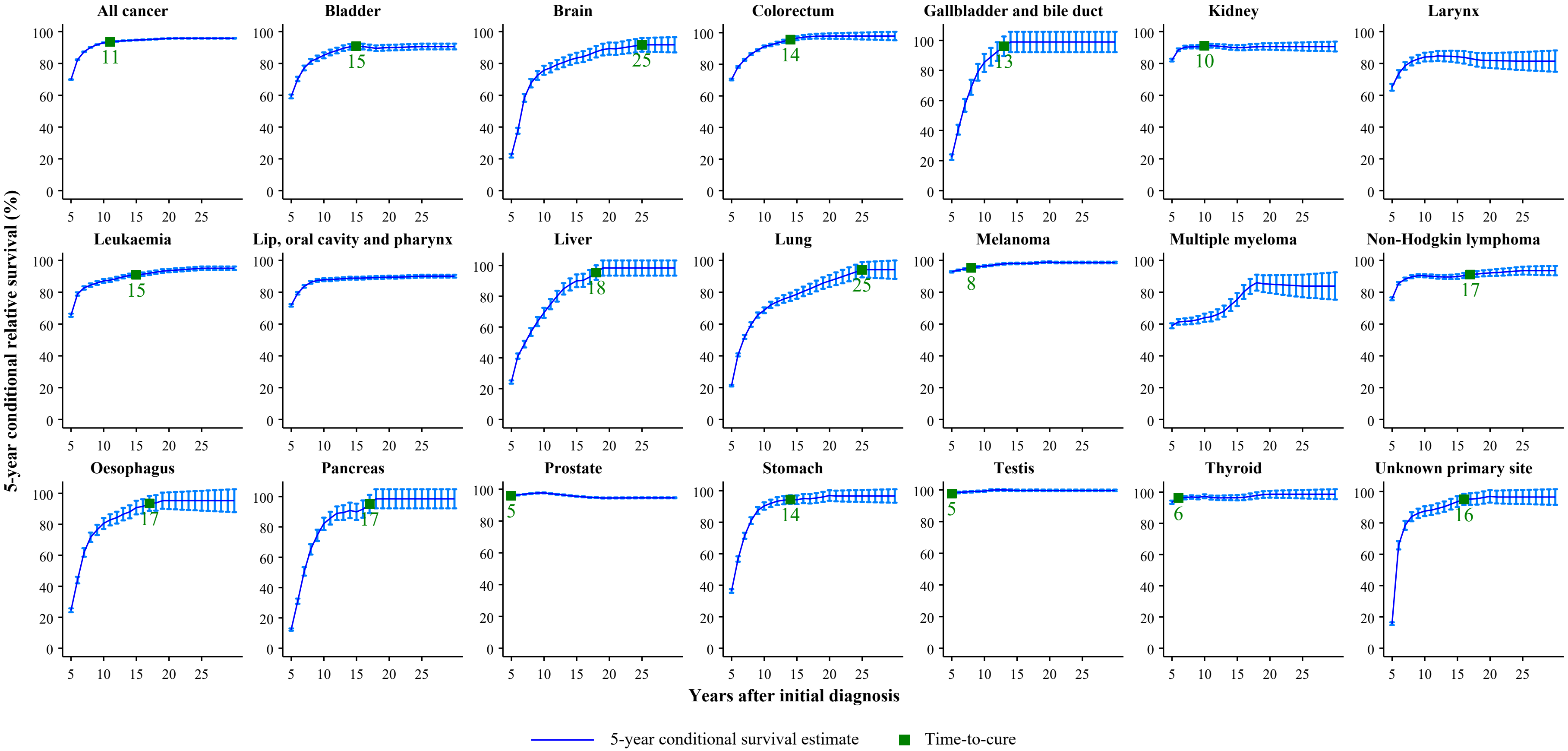

Time to cure was not reached for cancers of the larynx, lip, oral cavity and pharynx, and multiple myeloma.

B. Females

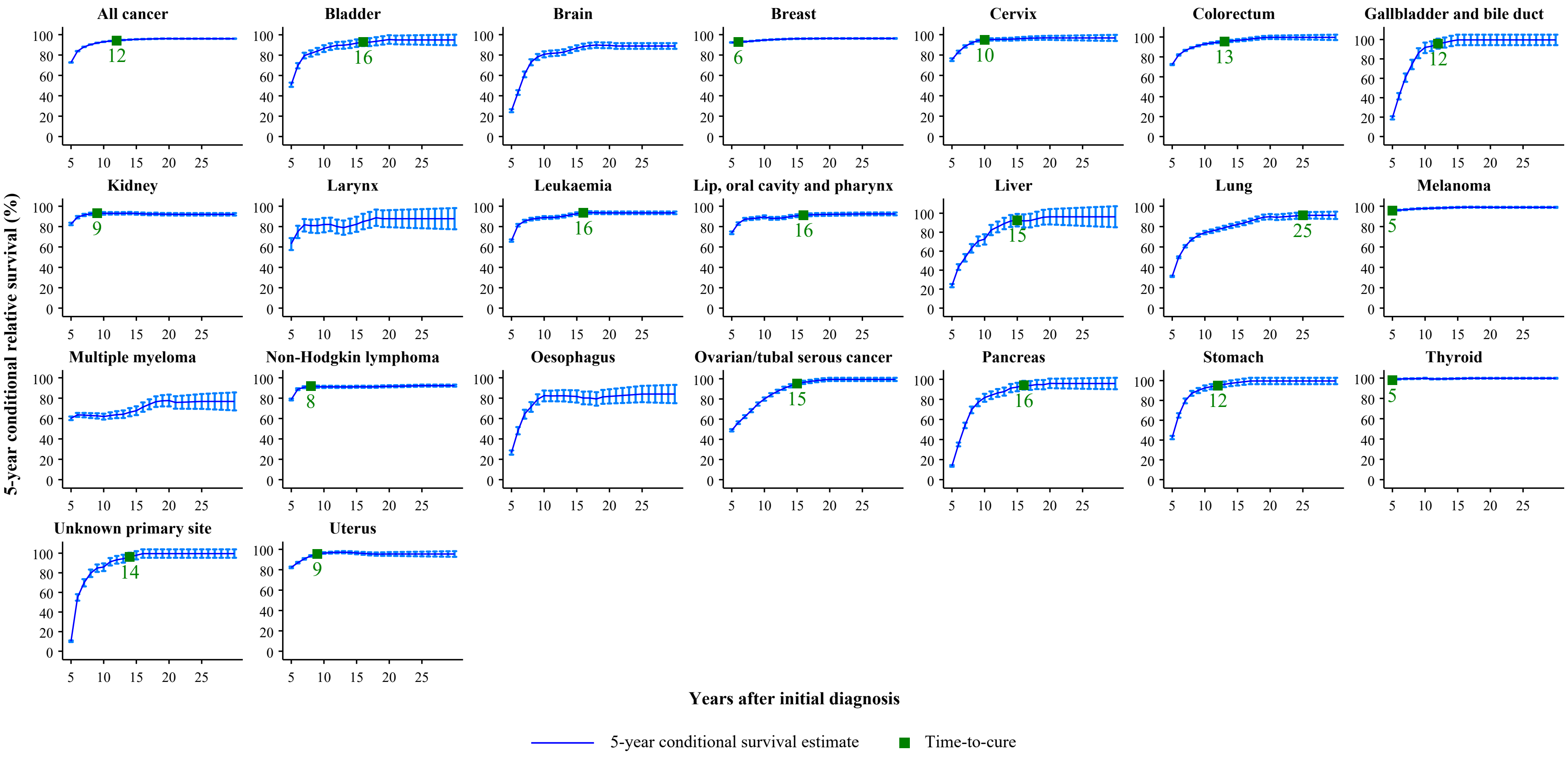

Time to cure was not reached for cancers of the brain, larynx, multiple myeloma, and oesophagus.

5. Sensitivity analyses

We conducted four sensitivity analyses by estimating the prevalence under different survival assumptions:

(1) survival remains constant from 2019 which is the last observed year.
(2) the increase in future extrapolated survival levels off from 2024.
(3) the increase in the future extrapolated survival levels off from 2034.
(4) the increase in the future extrapolated survival levels off from 2039.

We also conducted eight sensitivity analyses for estimating the number of people living beyond cancer (potentially cured) by applying alternative thresholds for 5-year conditional relative survival:

(1) exceeding 90% or exceeding 86% and reaching a plateau.
(2) exceeding 91% or exceeding 87% and reaching a plateau.
(3) exceeding 92% or exceeding 88% and reaching a plateau.
(4) exceeding 93% or exceeding 89% and reaching a plateau.
(5) exceeding 94% or exceeding 90% and reaching a plateau.
(6) exceeding 96% or exceeding 92% and reaching a plateau.
(7) exceeding 97% or exceeding 93% and reaching a plateau.
(8) exceeding 98% or exceeding 94% and reaching a plateau.

6. Limitations of projection methods

The main limitation of the current study, as is common for all modelling studies, is that the projections depend on several assumptions. These include the incidence projection assumptions, which have been extensively discussed in our previous publication.[8] Briefly, the age-risk effects were assumed to remain unchanged over time and reflect the general level of cancer risk in the population. For cancers other than lung and prostate cancer, the cohort and period effects of the most recently observed cohort and period were assumed to apply for future unobserved cohorts and periods.[8] For lung, prostate and breast cancers, the most recent trends in smoking exposure, PSA testing and breast cancer screening participation were assumed to continue into the future. Recent improvements in survival were assumed to continue to 2029 and then remain constant to 2050 in the main analyses. These assumptions do not capture the potential impacts of future changes in treatment effectiveness or the introduction of entirely new prevention or treatment paradigms or new screening programs.

For example, although the effects of the National Bowel Cancer Screening Program are captured in pre-2020 trends, the current analysis does not capture the impact of the full roll out from 2020 and the inclusion of people aged 45-49 from July 2024,[22] nor does it capture the potential effect of the new targeted lung cancer screening program introduced in July 2025.[14] Increased bowel cancer screening participation is expected to lead to earlier detection of colorectal cancer and improved survival outcomes,[15] which may increase future prevalence. However, the detection and removal of precancerous lesions through the screening program[23] may also reduce incidence and could offset increases in colorectal cancer prevalence. Moreover, the inclusion of younger age groups (45-49 years) in the screening program may contribute to a short-term rise in prevalence estimates by identifying additional new diagnoses that would otherwise have been diagnosed later (which would be offset by corresponding decreases in following years). As another example, the new targeted lung cancer screening program is now open to people aged 50-70 years who either currently smoke or who quit within the past 10 years, with a 30 pack-year smoking history (e.g. equivalent to smoking a pack of 20 cigarettes per day for 30 years).[14] The introduction of this screening program among high-risk populations is also likely to lead to initial increases in lung cancer incidence due to the detection of cancers earlier than would occur through symptomatic presentation. This initial increase in incidence would then be expected to be offset by corresponding decreases in the following years, with the effects stabilising after several years.[24] This is expected to result in increases in 1- and 5-year prevalence, as lung cancer cases detected at an early stage are often more treatable and have improved survival.[14] However, lead-time bias should be considered, as this may reflect shifts in cancer diagnosis and survival patterns rather than a true rise in cancer burden.[25] Over the longer term, recent and ongoing reductions in smoking exposure are expected to lower lung cancer incidence,[26] which may eventually offset the initial increase in lung cancer prevalence driven by screening and contribute to a stabilisation of prevalence trends. In addition, our projections do not account for the impact of the human papillomavirus (HPV) vaccination program introduced in Australia in 2007 targeting people aged 12-13 years nor the ongoing effects of the 2017 transition to HPV-based cervical screening in Australia; noting that previously performed explicit modelling of the effect of these transitions on cervical cancer incidence[17] can be used as a basis for future refined projections of people living with and beyond cervical cancer. Nonetheless, as the National HPV Vaccination Program from 2007 is targeting people aged 12-13 years,[16] it will most likely impact cervical cancer rates after 2044.[17] The estimated prevalence of cervical cancer is likely to decrease in the longer-term due to the combined impact of HPV vaccination

and cervical cancer screening, which reduce the risk of developing cervical cancer and progression from precancerous lesions.[16] Due to the complexity of the cancer prevalence measure, which depends on both incidence and survival, these projections could either overestimate or underestimate the true prevalence in the future. For example, improvements in survival through screening programs (e.g. stage shift) beyond those modelled in this study or in early detection could increase future numbers of people living with and beyond cancer in Australia, making the prevalence projections an underestimate. Oppositely, effects of recently introduced or future new cancer prevention strategies could reduce the number of new diagnoses, making the prevalence projections an overestimate. Thus, regular updates of long-term prevalence projections to incorporate the latest evidence are vital.

Another limitation of this study is that we derived overall UIs for the prevalence estimates based on separate UIs for incidence and survival, and assumed a perfect correlation between incidence and survival. Both incidence and survival are influenced by early detection practices. For example, higher opportunistic early detection can lead to both higher incidence and longer survival. However, the interdependence between incidence and survival is highly dependent on the cancer and the context (e.g. the underlying screening or clinical practice), and this approach might overstate the true width of the 95% UIs for prevalence. On the other hand, for simplicity, we estimated the UIs for cancer incidence and survival without resampling the data, and therefore we captured only some aspects of the uncertainty associated with the cancer incidence and survival data used. In addition, this study does not account for all non-melanoma skin cancers (NMSC), as NMSC are not notifiable diseases and so are not routinely collected by the population-based cancer registries, with only rare types of NMSC included in the national data.[7] It should be noted that NMSC are highly prevalent and therefore significantly contribute to health care cost and clinical workload, including dermatology services and surgical demand, thus separate projections for NMSC would be warranted in the future. Furthermore, another limitation of statistical modelling that relies on tabulated data is that the aggregated information does not account for individual-level characteristics and hence masks any disparities in the cancer burden for sub-populations, such as people with socioeconomic disadvantage. Disparities in cancer outcomes for Australians living in rural and remote communities, and for socioeconomically disadvantaged sub-populations are well-documented.[7,27-29] For all cancers combined, higher cancer incidence rates and poorer survival were observed for people living in areas with lower socioeconomic status (SES) compared with those living in high SES areas.[7] Similarly, Aboriginal and Torres

Strait Islander people experience higher overall cancer incidence and poorer survival compared with non-Aboriginal and Torres Strait Island Australians.[27] This limitation potentially affects the precision and generalisability of the 2050 prevalence projections if applied to sub-populations, which is a crucial consideration for equitable long-term healthcare planning. Thus, it would be ideal to construct dedicated cancer prevalence estimates for population groups experiencing such inequities, but unfortunately such estimates were beyond the scope of the current study. Due to the complexity of varying risk factors and access to care for these sub-populations, reducing the disparities in cancer outcomes remains a major challenge and achieving equity in cancer outcomes is a priority in the current Australian Cancer Plan 2023.[30] Our prevalence projections provide important population-level benchmark data against which to evaluate the impact of cancer control initiatives. Further research is vital to estimate future disparities in cancer prevalence and develop dedicated benchmarks for different priority populations, thereby supporting long-term health care planning.

7. Additional results

Table S4. Estimated cancer prevalence by sex and time since diagnosis, Australia, 2050

A. Persons

Cancer type	30-year prevalence Counts (95% UI)	Time since diagnosis 0 to 1 year Counts (95% UI)	%	>1 to 5 year Counts (95% UI)	%	>5 years Counts (95% UI)	%
**All cancers**	**2,617,016 (2,333,949 – 2,947,174)**	**203,036 (181,570 – 227,989)**	**7·8**	**587,314 (521,743 – 664,348)**	**22·4**	**1,826,666 (1,630,636 – 2,054,837)**	**69·8**
Bladder	32,623 (27,019 – 39,408)	3,851 (3,266 – 4,542)	11·8	8,946 (7,395 – 10,820)	27·4	19,826 (16,358 – 24,046)	60·8
Brain	13,111 (9,897 – 17,318)	1,997 (1,658 – 2,419)	15·2	3,129 (2,396 – 4,088)	23·9	7,985 (5,843 – 10,811)	60·9
Breast	485,135 (460,470 – 511,175)	29,475 (27,884 – 31,160)	6·1	104,115 (98,288 – 110,308)	21·5	351,545 (334,298 – 369,707)	72·5
Cervix	22,023 (17,050 – 28,509)	1,266 (954 – 1,676)	5·7	4,086 (3,048 – 5,476)	18·6	16,671 (13,048 – 21,357)	75·7
Colorectum	237,053 (209,185 – 269,057)	18,607 (16,460 – 21,069)	7·8	55,147 (48,332 – 63,027)	23·3	163,299 (144,393 – 184,961)	68·9
Gallbladder and bile duct	6,778 (4,813 – 9,345)	1,417 (1,141 – 1,764)	20·9	1,818 (1,318 – 2,470)	26·8	3,543 (2,354 – 5,111)	52·3
Kidney	108,101 (91,074 – 128,515)	7,830 (6,611 – 9,292)	7·2	25,406 (21,220 – 30,454)	23·5	74,865 (63,243 – 88,769)	69·3
Larynx	5,330 (4,002 – 7,200)	614 (475 – 789)	11·5	1,694 (1,285 – 2,273)	31·8	3,022 (2,242 – 4,138)	56·7
Leukaemia	87,858 (76,746 – 101,225)	7,292 (6,573 – 8,144)	8·3	19,838 (17,442 – 22,717)	22·6	60,728 (52,731 – 70,364)	69·1
Lip, oral cavity and pharynx	77,046 (65,052 – 91,344)	5,696 (4,788 – 6,784)	7·4	17,383 (14,438 – 20,949)	22·6	53,967 (45,826 – 63,611)	70.0
Liver	21,411 (15,643 – 29,657)	3,560 (2,797 – 4,581)	16·6	5,705 (4,219 – 7,821)	26·6	12,146 (8,627 – 17,255)	56·7
Lung	85,284 (73,647 – 98,657)	14,122 (12,511 – 15,953)	16·6	22,629 (19,547 – 26,179)	26·5	48,533 (41,589 – 56,525)	56·9
Melanoma	331,909 (306,494 – 360,106)	20,419 (18,867 – 22,134)	6·2	69,876 (64,227 – 76,162)	21·1	241,614 (223,400 – 261,810)	72·8
Multiple myeloma	33,636 (27,745 – 40,816)	3,844 (3,332 – 4,458)	11·4	10,499 (8,853 – 12,501)	31·2	19,293 (15,560 – 23,857)	57·4
Non-Hodgkin lymphoma	107,133 (91,522 – 125,644)	8,437 (7,254 – 9,828)	7·9	25,837 (21,945 – 30,474)	24·1	72,859 (62,323 – 85,342)	68.0
Oesophagus	10,405 (8,253 – 12,974)	1,839 (1,583 – 2,129)	17·7	2,678 (2,173 – 3,293)	25·7	5,888 (4,497 – 7,552)	56·6
Ovarian/tubal serous cancer	26,219 (20,775 – 33,306)	2,312 (1,874 – 2,874)	8·8	5,960 (4,665 – 7,693)	22·7	17,947 (14,236 – 22,739)	68·5
Pancreas	22,600 (18,573 – 27,287)	5,191 (4,570 – 5,896)	23.0	5,378 (4,441 – 6,473)	23·8	12,031 (9,562 – 14,918)	53·2
Prostate	468,991 (434,135 – 507,283)	31,494 (29,583 – 33,565)	6·7	105,646 (98,634 – 113,272)	22·5	331,851 (305,918 – 360,446)	70·8
Stomach	24,328 (20,435 – 28,844)	3,133 (2,777 – 3,534)	12·9	6,062 (5,134 – 7,141)	24·9	15,133 (12,524 – 18,169)	62·2
Testis	32,837 (28,974 – 37,236)	1,322 (1,150 – 1,515)	4.0	5,042 (4,390 – 5,798)	15·4	26,473 (23,434 – 29,923)	80·6
Thyroid	103,965 (88,027 – 123,107)	5,210 (4,318 – 6,304)	5.0	19,441 (16,070 – 23,585)	18·7	79,314 (67,639 – 93,218)	76·3
Unknown primary site	12,314 (9,508 – 15,867)	2,107 (1,704 – 2,612)	17·1	1,825 (1,337 – 2,462)	14·8	8,382 (6,467 – 10,793)	68·1
Uterus	83,281 (74,862 – 92,631)	4,909 (4,407 – 5,465)	5·9	16,127 (14,371 – 18,099)	19·4	62,245 (56,084 – 69,067)	74·7
Other cancers	177,645 (150,048 – 210,663)	17,092 (15,033 – 19,502)	9·6	43,047 (36,575 – 50,813)	24·2	117,506 (98,440 – 140,348)	66·1

B. Males

Cancer type	30-year prevalence Counts (95% UI)	Time since diagnosis 0 to 1 year Counts (95% UI)	%	>1 to 5 year Counts (95% UI)	%	>5 years Counts (95% UI)	%
**All cancers**	**1,334,966 (1,190,115 – 1,502,571)**	**109,248 (98,284 – 121,847)**	**8·2**	**305,872 (272,840 – 344,257)**	**22·9**	**919,846 (818,991 – 1,036,467)**	**68·9**
Bladder	27,577 (23,354 – 32,529)	3,078 (2,661 – 3,559)	11·2	7,312 (6,178 – 8,639)	26·5	17,187 (14,515 – 20,331)	62·3
Brain	8,582 (6,578 – 11,220)	1,237 (1,032 – 1,488)	14·4	1,874 (1,447 – 2,443)	21·8	5,471 (4,099 – 7,289)	63·7
Colorectum	131,585 (115,636 – 150,170)	9,990 (8,790 – 11,389)	7·6	29,363 (25,568 – 33,827)	22·3	92,232 (81,278 – 104,954)	70·1
Gallbladder and bile duct	3,341 (2,276 – 4,755)	725 (569 – 925)	21·7	982 (696 – 1,365)	29·4	1,634 (1,011 – 2,465)	48·9
Kidney	67,361 (57,851 – 78,513)	5,219 (4,500 – 6,059)	7·7	16,913 (14,445 – 19,809)	25·1	45,229 (38,906 – 52,645)	67·1
Larynx	4,763 (3,710 – 6,088)	537 (432 – 659)	11·3	1,482 (1,173 – 1,878)	31·1	2,744 (2,105 – 3,551)	57·6
Leukaemia	51,077 (45,775 – 57,275)	4,368 (4,047 – 4,736)	8·6	11,868 (10,756 – 13,155)	23·2	34,841 (30,972 – 39,384)	68·2
Lip, oral cavity and pharynx	57,995 (49,998 – 67,273)	3,921 (3,360 – 4,575)	6·8	11,995 (10,174 – 14,138)	20·7	42,079 (36,464 – 48,560)	72·6
Liver	13,935 (10,555 – 18,453)	2,493 (2,024 – 3,084)	17·9	4,152 (3,210 – 5,392)	29·8	7,290 (5,321 – 9,977)	52·3
Lung	40,273 (34,944 – 46,356)	7,751 (6,909 – 8,700)	19·2	10,814 (9,403 – 12,434)	26·9	21,708 (18,632 – 25,222)	53·9
Melanoma	189,301 (175,559 – 204,545)	11,945 (11,089 – 12,887)	6·3	40,137 (37,067 – 43,550)	21·2	137,219 (127,403 – 148,108)	72·5
Multiple myeloma	16,914 (14,674 – 19,453)	2,235 (2,045 – 2,449)	13·2	6,054 (5,411 – 6,771)	35·8	8,625 (7,218 – 10,233)	51·0
Non-Hodgkin lymphoma	61,704 (53,106 – 71,813)	4,747 (4,110 – 5,489)	7·7	14,328 (12,254 – 16,777)	23·2	42,629 (36,742 – 49,547)	69·1
Oesophagus	7,277 (5,900 – 8,893)	1,401 (1,226 – 1,597)	19·3	2,059 (1,711 – 2,473)	28·3	3,817 (2,963 – 4,823)	52·5
Pancreas	10,354 (8,483 – 12,537)	2,671 (2,359 – 3,024)	25·8	2,760 (2,296 – 3,306)	26·7	4,923 (3,828 – 6,207)	47·5
Prostate	468,991 (434,135 – 507,283)	31,494 (29,583 – 33,565)	6·7	105,646 (98,634 – 113,272)	22·5	331,851 (305,918 – 360,446)	70·8
Stomach	12,438 (10,543 – 14,597)	1,865 (1,676 – 2,076)	15·0	3,514 (3,034 – 4,061)	28·3	7,059 (5,833 – 8,460)	56·8
Testis	32,837 (28,974 – 37,236)	1,322 (1,150 – 1,515)	4·0	5,042 (4,390 – 5,798)	15·4	26,473 (23,434 – 29,923)	80·6
Thyroid	30,251 (24,354 – 37,679)	1,757 (1,394 – 2,220)	5·8	6,335 (4,996 – 8,060)	20·9	22,159 (17,964 – 27,399)	73·3
Unknown primary site	8,084 (6,381 – 10,209)	1,231 (1,007 – 1,512)	15·2	1,213 (913 – 1,600)	15·0	5,640 (4,461 – 7,097)	69·8
Other cancers	90,326 (77,329 – 105,694)	9,261 (8,321 – 10,339)	10·3	22,029 (19,084 – 25,509)	24·4	59,036 (49,924 – 69,846)	65·4

C. Females

Cancer type	30-year prevalence Counts (95% UI)	Time since diagnosis 0 to 1 year Counts (95% UI)	%	>1 to 5 year Counts (95% UI)	%	>5 years Counts (95% UI)	%
**All cancers**	**1,282,050 (1,143,834 – 1,444,603)**	**93,788 (83,286 – 106,142)**	**7·3**	**281,442 (248,903 – 320,091)**	**22.0**	**906,820 (811,645 – 1,018,370)**	**70·7**
Bladder	5,046 (3,665 – 6,879)	773 (605 – 983)	15·3	1,634 (1,217 – 2,181)	32·4	2,639 (1,843 – 3,715)	52·3
Brain	4,529 (3,319 – 6,098)	760 (626 – 931)	16·8	1,255 (949 – 1,645)	27·7	2,514 (1,744 – 3,522)	55·5
Breast	485,135 (460,470 – 511,175)	29,475 (27,884 – 31,160)	6·1	104,115 (98,288 – 110,308)	21·5	351,545 (334,298 – 369,707)	72·5
Cervix	22,023 (17,050 – 28,509)	1,266 (954 – 1,676)	5·7	4,086 (3,048 – 5,476)	18·6	16,671 (13,048 – 21,357)	75·7
Colorectum	105,468 (93,549 – 118,887)	8,617 (7,670 – 9,680)	8·2	25,784 (22,764 – 29,200)	24·4	71,067 (63,115 – 80,007)	67·4
Gallbladder and bile duct	3,437 (2,537 – 4,590)	692 (572 – 839)	20·1	836 (622 – 1,105)	24·3	1,909 (1,343 – 2,646)	55·5
Kidney	40,740 (33,223 – 50,002)	2,611 (2,111 – 3,233)	6·4	8,493 (6,775 – 10,645)	20·8	29,636 (24,337 – 36,124)	72·7
Larynx	567 (292 – 1,112)	77 (43 – 130)	13·6	212 (112 – 395)	37·4	278 (137 – 587)	49.0
Leukaemia	36,781 (30,971 – 43,950)	2,924 (2,526 – 3,408)	7·9	7,970 (6,686 – 9,562)	21·7	25,887 (21,759 – 30,980)	70·4
Lip, oral cavity and pharynx	19,051 (15,054 – 24,071)	1,775 (1,428 – 2,209)	9·3	5,388 (4,264 – 6,811)	28·3	11,888 (9,362 – 15,051)	62·4
Liver	7,476 (5,088 – 11,204)	1,067 (773 – 1,497)	14·3	1,553 (1,009 – 2,429)	20·8	4,856 (3,306 – 7,278)	65.0
Lung	45,011 (38,703 – 52,301)	6,371 (5,602 – 7,253)	14·2	11,815 (10,144 – 13,745)	26·2	26,825 (22,957 – 31,303)	59·6
Melanoma	142,608 (130,935 – 155,561)	8,474 (7,778 – 9,247)	5·9	29,739 (27,160 – 32,612)	20·9	104,395 (95,997 – 113,702)	73·2
Multiple myeloma	16,722 (13,071 – 21,363)	1,609 (1,287 – 2,009)	9·6	4,445 (3,442 – 5,730)	26·6	10,668 (8,342 – 13,624)	63·8
Non-Hodgkin lymphoma	45,429 (38,416 – 53,831)	3,690 (3,144 – 4,339)	8·1	11,509 (9,691 – 13,697)	25·3	30,230 (25,581 – 35,795)	66·5
Oesophagus	3,128 (2,353 – 4,081)	438 (357 – 532)	14.0	619 (462 – 820)	19·8	2,071 (1,534 – 2,729)	66·2
Ovarian/tubal serous cancer	26,219 (20,775 – 33,306)	2,312 (1,874 – 2,874)	8·8	5,960 (4,665 – 7,693)	22·7	17,947 (14,236 – 22,739)	68·5
Pancreas	12,246 (10,090 – 14,750)	2,520 (2,211 – 2,872)	20·6	2,618 (2,145 – 3,167)	21·4	7,108 (5,734 – 8,711)	58.0
Stomach	11,890 (9,892 – 14,247)	1,268 (1,101 – 1,458)	10·7	2,548 (2,100 – 3,080)	21·4	8,074 (6,691 – 9,709)	67·9
Thyroid	73,714 (63,673 – 85,428)	3,453 (2,924 – 4,084)	4·7	13,106 (11,074 – 15,525)	17·8	57,155 (49,675 – 65,819)	77·5
Unknown primary site	4,230 (3,127 – 5,658)	876 (697 – 1,100)	20·7	612 (424 – 862)	14·5	2,742 (2,006 – 3,696)	64·8
Uterus	83,281 (74,862 – 92,631)	4,909 (4,407 – 5,465)	5·9	16,127 (14,371 – 18,099)	19·4	62,245 (56,084 – 69,067)	74·7
Other cancers	87,319 (72,719 – 104,969)	7,831 (6,712 – 9,163)	9.0	21,018 (17,491 – 25,304)	24·1	58,470 (48,516 – 70,502)	67.0

The uncertainty intervals (UIs) for the estimated prevalence were estimated based on the UIs of the number of new cases and survival rates.

Table S5. Estimated cancer prevalence by sex and prevalence duration in Australia, 2025 to 2050

A. Males

	30-year prevalence			5-year prevalence			1-year prevalence		
**Cancer type**	**Counts (95% UI) in 2025**	**Counts (95% UI) in 2050**	**Change %**	**Counts (95% UI) in 2025**	**Counts (95% UI) in 2050**	**Change %**	**Counts (95% UI) in 2025**	**Counts (95% UI) in 2050**	**Change %**
**All cancers for males**	**862,322 (803,370 – 924,848)**	**1,334,966 (1,190,115 – 1,502,571)**	**54·8**	**306,252 (282,697 – 332,360)**	**415,120 (371,124 – 466,104)**	**35·5**	**79,418 (73,503 – 85,971)**	**109,248 (98,284 – 121,847)**	**37·6**
Bladder	18,348 (16,486 – 20,311)	27,577 (23,354 – 32,529)	50·3	7,660 (6,934 – 8,466)	10,390 (8,839 – 12,198)	35·6	2,234 (2,039 – 2,447)	3,078 (2,661 – 3,559)	37·8
Brain	5,375 (4,331 – 6,535)	8,582 (6,578 – 11,220)	59·7	2,381 (2,008 – 2,823)	3,111 (2,479 – 3,931)	30·7	945 (828 – 1,078)	1,237 (1,032 – 1,488)	30·9
Colorectum	94,715 (89,065 – 100,663)	131,585 (115,636 – 150,170)	38·9	31,813 (29,673 – 34,134)	39,353 (34,358 – 45,216)	23·7	7,988 (7,441 – 8,586)	9,990 (8,790 – 11,389)	25·1
Gallbladder and bile duct	2,090 (1,533 – 2,673)	3,341 (2,276 – 4,755)	59·9	1,125 (904 – 1,393)	1,707 (1,265 – 2,290)	51·7	471 (397 – 560)	725 (569 – 925)	53·9
Kidney	31,373 (28,498 – 34,436)	67,361 (57,851 – 78,513)	114·7	12,166 (11,048 – 13,419)	22,132 (18,945 – 25,868)	81·9	2,918 (2,652 – 3,219)	5,219 (4,500 – 6,059)	78·9
Larynx	5,207 (4,433 – 6,038)	4,763 (3,710 – 6,088)	-8·5	1,844 (1,574 – 2,164)	2,019 (1,605 – 2,537)	9·5	473 (410 – 549)	537 (432 – 659)	13·5
Leukaemia	28,127 (25,496 – 30,935)	51,077 (45,775 – 57,275)	81·6	10,732 (9,901 – 11,669)	16,236 (14,803 – 17,891)	51·3	2,868 (2,681 – 3,078)	4,368 (4,047 – 4,736)	52·3
Lip, oral cavity and pharynx	38,506 (35,413 – 41,631)	57,995 (49,998 – 67,273)	50·6	12,635 (11,528 – 13,853)	15,916 (13,534 – 18,713)	26·0	3,103 (2,827 – 3,405)	3,921 (3,360 – 4,575)	26·4
Liver	7,299 (6,089 – 8,750)	13,935 (10,555 – 18,453)	90·9	4,489 (3,896 – 5,211)	6,645 (5,234 – 8,476)	48·0	1,691 (1,492 – 1,925)	2,493 (2,024 – 3,084)	47·4
Lung	23,536 (21,343 – 25,858)	40,273 (34,944 – 46,356)	71·1	13,680 (12,648 – 14,793)	18,565 (16,312 – 21,134)	35·7	5,655 (5,278 – 6,059)	7,751 (6,909 – 8,700)	37·1
Melanoma	137,170 (131,178 – 143,335)	189,301 (175,559 – 204,545)	38·0	43,366 (41,317 – 45,558)	52,082 (48,156 – 56,437)	20·1	9,991 (9,519 – 10,497)	11,945 (11,089 – 12,887)	19·6
Multiple myeloma	8,692 (7,601 – 9,877)	16,914 (14,674 – 19,453)	94·6	5,141 (4,685 – 5,633)	8,289 (7,456 – 9,220)	61·2	1,394 (1,287 – 1,506)	2,235 (2,045 – 2,449)	60·3
Non-Hodgkin lymphoma	37,488 (34,173 – 41,015)	61,704 (53,106 – 71,813)	64·6	14,012 (12,768 – 15,408)	19,075 (16,364 – 22,266)	36·1	3,493 (3,186 – 3,838)	4,747 (4,110 – 5,489)	35·9
Oesophagus	4,505 (3,770 – 5,333)	7,277 (5,900 – 8,893)	61·5	2,465 (2,172 – 2,797)	3,460 (2,937 – 4,070)	40·4	985 (894 – 1,089)	1,401 (1,226 – 1,597)	42·2
Pancreas	5,188 (4,398 – 6,007)	10,354 (8,483 – 12,537)	99·6	3,415 (3,037 – 3,822)	5,431 (4,655 – 6,330)	59·0	1,672 (1,530 – 1,828)	2,671 (2,359 – 3,024)	59·7
Prostate	309,663 (295,376 – 324,818)	468,991 (434,135 – 507,283)	51·5	101,035 (94,030 – 108,743)	137,140 (128,217 – 146,837)	35·7	22,591 (21,018 – 24,319)	31,494 (29,583 – 33,565)	39·4
Stomach	8,807 (7,676 – 10,014)	12,438 (10,543 – 14,597)	41·2	3,865 (3,498 – 4,273)	5,379 (4,710 – 6,137)	39·2	1,310 (1,213 – 1,418)	1,865 (1,676 – 2,076)	42·4
Testis	20,830 (19,251 – 22,497)	32,837 (28,974 – 37,236)	57·6	4,684 (4,233 – 5,182)	6,364 (5,540 – 7,313)	35·9	986 (888 – 1,094)	1,322 (1,150 – 1,515)	34·1
Thyroid	13,779 (12,043 – 15,584)	30,251 (24,354 – 37,679)	119·5	5,089 (4,408 – 5,889)	8,092 (6,390 – 10,280)	59·0	1,159 (1,002 – 1,348)	1,757 (1,394 – 2,220)	51·6
Unknown primary site	4,350 (3,561 – 5,243)	8,084 (6,381 – 10,209)	85·8	1,897 (1,609 – 2,230)	2,444 (1,920 – 3,112)	28·8	912 (800 – 1,039)	1,231 (1,007 – 1,512)	35·0
Other cancers	57,274 (51,656 – 63,295)	90,326 (77,329 – 105,694)	57·7	22,758 (20,826 – 24,900)	31,290 (27,405 – 35,848)	37·5	6,579 (6,121 – 7,089)	9,261 (8,321 – 10,339)	40·8

B. Females

Cancer type	30-year prevalence Counts (95% UI) in 2025	30-year prevalence Counts (95% UI) in 2050	30-year prevalence Change %	5-year prevalence Counts (95% UI) in 2025	5-year prevalence Counts (95% UI) in 2050	5-year prevalence Change %	1-year prevalence Counts (95% UI) in 2025	1-year prevalence Counts (95% UI) in 2050	1-year prevalence Change %
**All cancers**	**803,698 (750,455 – 860,052)**	**1,282,050 (1,143,834 – 1,444,603)**	**59·5**	**265,684 (246,506 – 287,003)**	**375,230 (332,189 – 426,233)**	**41·2**	**66,469 (61,630 – 71,853)**	**93,788 (83,286 – 106,142)**	**41·1**
Bladder	4,475 (3,608 – 5,370)	5,046 (3,665 – 6,879)	12·8	1,812 (1,521 – 2,159)	2,407 (1,822 – 3,164)	32·8	570 (493 – 663)	773 (605 – 983)	35·6
Brain	3,659 (2,846 – 4,593)	4,529 (3,319 – 6,098)	23·8	1,644 (1,364 – 1,992)	2,015 (1,575 – 2,576)	22·6	617 (533 – 719)	760 (626 – 931)	23·2
Breast	298,109 (288,918 – 307,472)	485,135 (460,470 – 511,175)	62·7	90,991 (87,896 – 94,197)	133,590 (126,172 – 141,468)	46·8	20,225 (19,525 – 20,950)	29,475 (27,884 – 31,160)	45·7
Cervix	15,776 (13,935 – 17,789)	22,023 (17,050 – 28,509)	39·6	3,927 (3,326 – 4,637)	5,352 (4,002 – 7,152)	36·3	917 (771 – 1,088)	1,266 (954 – 1,676)	38·1
Colorectum	79,278 (74,286 – 84,497)	105,468 (93,549 – 118,887)	33.0	27,066 (25,203 – 29,073)	34,401 (30,434 – 38,880)	27·1	6,789 (6,319 – 7,295)	8,617 (7,670 – 9,680)	26·9
Gallbladder and bile duct	1,840 (1,362 – 2,332)	3,437 (2,537 – 4,590)	86·8	993 (816 – 1,212)	1,528 (1,194 – 1,944)	53·9	440 (378 – 513)	692 (572 – 839)	57·3
Kidney	17,535 (15,436 – 19,830)	40,740 (33,223 – 50,002)	132·3	6,228 (5,449 – 7,134)	11,104 (8,886 – 13,878)	78·3	1,486 (1,297 – 1,700)	2,611 (2,111 – 3,233)	75·7
Larynx	664 (385 – 890)	567 (292 – 1,112)	-14·6	226 (150 – 342)	289 (155 – 525)	27·9	57 (41 – 86)	77 (43 – 130)	35·1
Leukaemia	18,467 (16,199 – 21,015)	36,781 (30,971 – 43,950)	99·2	6,811 (6,030 – 7,728)	10,894 (9,212 – 12,970)	59·9	1,808 (1,624 – 2,026)	2,924 (2,526 – 3,408)	61·7
Lip, oral cavity and pharynx	15,545 (13,686 – 17,425)	19,051 (15,054 – 24,071)	22·6	4,838 (4,202 – 5,554)	7,163 (5,692 – 9,020)	48·1	1,181 (1,025 – 1,358)	1,775 (1,428 – 2,209)	50·3
Liver	2,523 (1,853 – 3,396)	7,476 (5,088 – 11,204)	196·3	1,549 (1,227 – 1,982)	2,620 (1,782 – 3,926)	69·1	630 (521 – 768)	1,067 (773 – 1,497)	69·4
Lung	25,084 (22,758 – 27,553)	45,011 (38,703 – 52,301)	79·4	14,223 (13,098 – 15,433)	18,186 (15,746 – 20,998)	27·9	5,000 (4,637 – 5,393)	6,371 (5,602 – 7,253)	27·4
Melanoma	112,722 (107,382 – 118,184)	142,608 (130,935 – 155,561)	26·5	31,358 (29,618 – 33,219)	38,213 (34,938 – 41,859)	21·9	6,996 (6,600 – 7,421)	8,474 (7,778 – 9,247)	21·1
Multiple myeloma	6,728 (5,651 – 7,948)	16,722 (13,071 – 21,363)	148·5	3,693 (3,211 – 4,240)	6,054 (4,729 – 7,739)	63·9	987 (870 – 1,120)	1,609 (1,287 – 2,009)	63.0
Non-Hodgkin lymphoma	29,822 (27,069 – 32,811)	45,429 (38,416 – 53,831)	52·3	10,687 (9,656 – 11,855)	15,199 (12,835 – 18,036)	42·2	2,605 (2,353 – 2,890)	3,690 (3,144 – 4,339)	41·7
Oesophagus	1,714 (1,261 – 2,193)	3,128 (2,353 – 4,081)	82·5	809 (656 – 986)	1,057 (819 – 1,352)	30·7	321 (273 – 376)	438 (357 – 532)	36·4
Ovarian/tubal serous cancer	16,005 (13,902 – 18,347)	26,219 (20,775 – 33,306)	63·8	5,957 (5,196 – 6,842)	8,272 (6,539 – 10,567)	38·9	1,660 (1,464 – 1,888)	2,312 (1,874 – 2,874)	39·3
Pancreas	4,815 (4,033 – 5,625)	12,246 (10,090 – 14,750)	154·3	3,120 (2,761 – 3,520)	5,138 (4,356 – 6,039)	64·7	1,505 (1,372 – 1,651)	2,520 (2,211 – 2,872)	67·4
Stomach	5,544 (4,679 – 6,467)	11,890 (9,892 – 14,247)	114·5	2,282 (2,006 – 2,597)	3,816 (3,201 – 4,538)	67·2	743 (673 – 824)	1,268 (1,101 – 1,458)	70·7
Thyroid	44,049 (40,774 – 47,465)	73,714 (63,673 – 85,428)	67·3	12,834 (11,656 – 14,143)	16,559 (13,998 – 19,609)	29.0	2,757 (2,488 – 3,059)	3,453 (2,924 – 4,084)	25·2
Unknown primary site	2,755 (2,148 – 3,437)	4,230 (3,127 – 5,658)	53·5	1,226 (1,014 – 1,477)	1,488 (1,121 – 1,962)	21·4	690 (599 – 795)	876 (697 – 1,100)	27.0
Uterus	44,447 (41,573 – 47,425)	83,281 (74,862 – 92,631)	87·4	13,917 (12,919 – 14,995)	21,036 (18,778 – 23,564)	51·2	3,268 (3,035 – 3,521)	4,909 (4,407 – 5,465)	50·2
Other cancers	52,142 (46,711 – 57,988)	87,319 (72,719 – 104,969)	67·5	19,493 (17,531 – 21,686)	28,849 (24,203 – 34,467)	48.0	5,217 (4,739 – 5,749)	7,831 (6,712 – 9,163)	50·1

The uncertainty intervals (UIs) for the estimated prevalence were estimated based on the UIs of the number of new cases and survival rates.

Table S6. Estimated cancer prevalence by sex, age group and prevalence duration in Australia, 2025 and 2050

A. Males

	30-year prevalence			5-year prevalence			1-year prevalence		
	Number of people with a past cancer diagnosis (95% UI)	% of total people with a past cancer diagnosis	% of all Australians of that age group	Number of people with a past cancer diagnosis (95% UI)	% of total people with a past cancer diagnosis	% of all Australians of that age group	Number of people with a past cancer diagnosis (95% UI)	% of total people with a past cancer diagnosis	% of all Australians of that age group
**Year 2025**									
<15 years	2,750 (2,059 – 3,607)	0.3	0·1	1,529 (1,118 – 2,090)	0·5	0·1	381 (281 – 523)	0·5	0·0
15-29 years	8,932 (7,164 – 10,783)	1·0	0·3	3,999 (3,288 – 4,915)	1·3	0·1	1,074 (902 – 1,292)	1·4	0·0
30-39 years	17,858 (15,576 – 20,252)	2·1	0·9	8,168 (7,291 – 9,195)	2·7	0·4	1,973 (1,763 – 2,216)	2·5	0·1
40-49 years	33,949 (30,421 – 37,630)	3·9	1·9	13,774 (12,351 – 15,370)	4·5	0·8	3,593 (3,230 – 4,004)	4·5	0·2
50-59 years	73,993 (67,814 – 80,632)	8·6	4·7	32,975 (29,992 – 36,304)	10·8	2·1	8,974 (8,169 – 9,869)	11·3	0·6
60-69 years	174,740 (162,380 – 188,019)	20·3	12·5	77,630 (71,148 – 84,791)	25·3	5·5	20,307 (18,618 – 22,175)	25·6	1·4
70-79 years	276,868 (262,080 – 292,449)	32·1	26·6	103,879 (97,677 – 110,561)	33·9	10·0	25,525 (24,093 – 27,064)	32·1	2·5
80+ years	273,232 (255,876 – 291,476)	31·7	50·9	64,298 (59,832 – 69,134)	21·0	12·0	17,591 (16,447 – 18,828)	22·1	3·3
**Total**	**862,322 (803,370 – 924,848)**	**100·0**	**6·2**	**306,252 (282,697 – 332,360)**	**100·0**	**2·2**	**79,418 (73,503 – 85,971)**	**100·0**	**0·6**
**Year 2050**									
<15 years	3,572 (2,380 – 5,410)	0·3	0·1	1,944 (1,293 – 2,945)	0·5	0·1	479 (320 – 718)	0·4	0·0
15-29 years	12,590 (9,028 – 17,938)	0·9	0·3	5,124 (3,866 – 6,983)	1·2	0·1	1,372 (1,081 – 1,780)	1·3	0·0
30-39 years	24,715 (20,323 – 30,515)	1·9	1·0	10,627 (9,130 – 12,430)	2·6	0·4	2,533 (2,186 – 2,963)	2·3	0·1
40-49 years	52,687 (44,486 – 62,771)	3·9	2·2	20,471 (17,289 – 24,356)	4·9	0·9	5,330 (4,521 – 6,309)	4·9	0·2
50-59 years	108,214 (93,382 – 125,809)	8·1	4·9	44,004 (38,333 – 50,621)	10·6	2·0	11,776 (10,366 – 13,396)	10·8	0·5
60-69 years	214,023 (188,752 – 243,003)	16·0	11·7	92,224 (81,870 – 104,004)	22·2	5·0	24,283 (21,647 – 27,269)	22·2	1·3
70-79 years	342,076 (308,149 – 380,297)	25·6	25·4	124,856 (114,122 – 136,920)	30·1	9·3	30,985 (28,449 – 33,820)	28·4	2·3
80+ years	577,089 (523,615 – 636,828)	43·2	54·9	115,870 (105,221 – 127,845)	27·9	11·0	32,490 (29,714 – 35,592)	29·7	3·1
**Total**	**1,334,966 (1,190,115 – 1,502,571)**	**100·0**	**7·3**	**415,120 (371,124 – 466,104)**	**100·0**	**2·3**	**109,248 (98,284 – 121,847)**	**100·0**	**0·6**

B. Females

	30-year prevalence			5-year prevalence			1-year prevalence		
	**Number of people with a past cancer diagnosis (95% UI)**	**% of total people with a past cancer diagnosis**	**% of all Australians of that age group**	**Number of people with a past cancer diagnosis (95% UI)**	**% of total people with a past cancer diagnosis**	**% of all Australians of that age group**	**Number of people with a past cancer diagnosis (95% UI)**	**% of total people with a past cancer diagnosis**	**% of all Australians of that age group**
**Year 2025**									
<15 years	2,346 (1,756 – 3,109)	0·3	0·1	1,320 (972 – 1,821)	0·5	0·1	330 (242 – 454)	0·5	0.0
15-29 years	8,190 (6,499 – 9,955)	1.0	0·3	3,851 (3,139 – 4,771)	1·4	0·1	1,058 (883 – 1,283)	1·6	0.0
30-39 years	21,902 (19,260 – 24,632)	2·7	1·1	11,521 (10,366 – 12,835)	4·3	0·6	2,977 (2,690 – 3,298)	4·5	0·1
40-49 years	54,762 (50,223 – 59,572)	6·8	3·1	25,546 (23,577 – 27,743)	9·6	1·4	6,334 (5,848 – 6,878)	9·5	0·4
50-59 years	111,881 (104,919 – 119,172)	13·9	6·8	44,462 (41,591 – 47,610)	16·7	2·7	10,579 (9,872 – 11,360)	15·9	0·6
60-69 years	180,859 (170,810 – 191,424)	22·5	12·1	63,641 (59,737 – 67,905)	24.0	4·3	15,475 (14,490 – 16,550)	23·3	1.0
70-79 years	215,911 (204,065 – 228,378)	26·9	19·1	69,766 (65,343 – 74,539)	26·3	6·2	16,887 (15,780 – 18,092)	25·4	1·5
80+ years	207,847 (192,923 – 223,810)	25·9	29·8	45,577 (41,781 – 49,779)	17·2	6·5	12,829 (11,825 – 13,938)	19·3	1·8
**Total**	**803,698 (750,455 – 860,052)**	**100.0**	**5·7**	**265,684 (246,506 – 287,003)**	**100.0**	**1·9**	**66,469 (61,630 – 71,853)**	**100.0**	**0·5**
**Year 2050**									
<15 years	2,993 (1,975 – 4,642)	0·2	0·1	1,657 (1,103 – 2,563)	0·4	0·1	410 (278 – 622)	0·4	0.0
15-29 years	11,120 (7,995 – 15,767)	0·9	0·3	5,026 (3,812 – 6,776)	1·3	0·1	1,369 (1,083 – 1,769)	1·5	0.0
30-39 years	28,538 (23,691 – 34,874)	2·2	1·1	14,700 (12,691 – 17,161)	3·9	0·6	3,755 (3,272 – 4,336)	4.0	0·1
40-49 years	78,997 (68,557 – 91,710)	6·2	3·3	36,288 (32,011 – 41,391)	9·7	1·5	8,975 (7,946 – 10,197)	9·6	0·4
50-59 years	164,922 (146,165 – 186,979)	12·9	7·2	61,736 (54,905 – 69,738)	16·5	2·7	14,433 (12,851 – 16,274)	15·4	0·6
60-69 years	252,335 (225,727 – 283,194)	19·7	12·7	82,377 (73,260 – 93,038)	22.0	4·2	19,777 (17,586 – 22,334)	21·1	1.0
70-79 years	305,173 (275,669 – 339,229)	23·8	20·3	90,352 (80,812 – 101,438)	24·1	6.0	21,673 (19,369 – 24,341)	23·1	1·4
80+ years	437,972 (394,055 – 488,208)	34·2	31·9	83,094 (73,595 – 94,128)	22·1	6.0	23,396 (20,901 – 26,269)	24·9	1·7
**Total**	**1,282,050 (1,143,834 – 1,444,603)**	**100.0**	**6·8**	**375,230 (332,189 – 426,233)**	**100.0**	**2.0**	**93,788 (83,286 – 106,142)**	**100.0**	**0·5**

Percentages may not sum to 100% due to rounding.

Table S7. Estimated cancer prevalence by age groups and prevalence duration in Australia, 2025 to 2050

A. 30-year prevalence

	Age <60 years			Age 60- 79 years			Age 80+ years		
Cancer type	Counts (95% UI) in 2025	Counts (95% UI) in 2050	Change %	Counts (95% UI) in 2025	Counts (95% UI) in 2050	Change %	Counts (95% UI) in 2025	Counts (95% UI) in 2050	Change %
**All cancers**	**336,563 (305,691 – 369,344)**	**488,348 (417,982 – 576,415)**	**45.1**	**848,378 (799,335 – 900,270)**	**1,113,607 (998,297 – 1,245,723)**	**31.3**	**481,079 (448,799 – 515,286)**	**1,015,061 (917,670 – 1,125,036)**	**111.0**
Bladder	1,396 (1,069 – 1,636)	1,985 (1,563 – 2,530)	42.2	10,621 (9,503 – 11,839)	12,598 (10,444 – 15,205)	18.6	10,806 (9,522 – 12,206)	18,040 (15,012 – 21,673)	66.9
Brain	5,553 (4,425 – 6,837)	7,794 (5,812 – 10,499)	40.4	2,937 (2,376 – 3,553)	4,023 (3,137 – 5,106)	37.0	544 (376 – 738)	1,294 (948 – 1,713)	137.9
Breast	68,140 (65,583 – 70,742)	101,359 (94,946 – 108,200)	48.8	157,709 (153,608 – 161,878)	233,831 (221,414 – 246,964)	48.3	72,260 (69,727 – 74,852)	149,945 (144,110 – 156,011)	107.5
Cervix	8,948 (7,931 – 10,060)	11,751 (9,046 – 15,287)	31.3	5,736 (5,080 – 6,445)	8,352 (6,545 – 10,694)	45.6	1,092 (924 – 1,284)	1,920 (1,459 – 2,528)	75.8
Colorectum	24,806 (22,449 – 27,328)	50,369 (41,938 – 60,649)	103.1	78,164 (73,881 – 82,644)	86,300 (76,859 – 96,940)	10.4	71,023 (67,021 – 75,188)	100,384 (90,388 – 111,468)	41.3
Gallbladder and bile duct	416 (267 – 491)	594 (418 – 840)	42.8	2,173 (1,688 – 2,706)	3,204 (2,350 – 4,317)	47.4	1,341 (940 – 1,808)	2,980 (2,045 – 4,188)	122.2
Kidney	10,816 (9,321 – 12,459)	18,900 (15,351 – 23,429)	74.7	26,449 (24,274 – 28,758)	54,158 (45,614 – 64,339)	104.8	11,643 (10,339 – 13,049)	35,043 (30,109 – 40,747)	201.0
Larynx	514 (372 – 623)	612 (461 – 827)	19.1	3,605 (2,990 – 4,204)	3,134 (2,363 – 4,230)	-13.1	1,752 (1,456 – 2,101)	1,584 (1,178 – 2,143)	-9.6
Leukaemia	13,618 (11,501 – 16,043)	21,437 (17,081 – 27,300)	57.4	20,866 (19,335 – 22,472)	33,917 (30,655 – 37,577)	62.5	12,110 (10,859 – 13,435)	32,504 (29,010 – 36,348)	168.4
Lip, oral cavity and pharynx	12,399 (10,973 – 13,664)	15,111 (12,463 – 18,338)	21.9	29,515 (27,240 – 31,911)	35,995 (30,416 – 42,637)	22.0	12,137 (10,886 – 13,481)	25,940 (22,173 – 30,369)	113.7
Liver	1,828 (1,343 – 2,547)	3,367 (2,306 – 5,362)	84.2	6,421 (5,465 – 7,505)	10,499 (7,786 – 14,202)	63.5	1,573 (1,134 – 2,094)	7,545 (5,551 – 10,093)	379.7
Lung	5,202 (4,521 – 5,927)	7,976 (6,696 – 9,506)	53.3	28,427 (26,202 – 30,790)	38,786 (33,535 – 44,826)	36.4	14,991 (13,378 – 16,694)	38,522 (33,416 – 44,325)	157.0
Melanoma	60,513 (56,879 – 64,243)	72,340 (65,509 – 80,539)	19.5	120,097 (115,618 – 124,690)	121,754 (112,840 – 131,380)	1.4	69,282 (66,063 – 72,586)	137,815 (128,145 – 148,187)	98.9
Multiple myeloma	2,010 (1,676 – 2,415)	3,345 (2,711 – 4,158)	66.4	9,340 (8,293 – 10,495)	16,719 (13,949 – 20,148)	79.0	4,070 (3,283 – 4,915)	13,572 (11,085 – 16,510)	233.5
Non-Hodgkin lymphoma	14,090 (12,338 – 16,028)	20,205 (16,623 – 24,827)	43.4	34,343 (31,838 – 36,989)	45,327 (39,105 – 52,531)	32.0	18,877 (17,066 – 20,809)	41,601 (35,794 – 48,286)	120.4
Oesophagus	645 (521 – 765)	857 (684 – 1,065)	32.9	3,691 (3,105 – 4,331)	4,774 (3,895 – 5,821)	29.3	1,883 (1,405 – 2,430)	4,774 (3,674 – 6,088)	153.5
Ovarian/tubal serous cancer	4,760 (3,964 – 5,675)	7,960 (5,649 – 11,294)	67.2	8,004 (7,151 – 8,921)	11,303 (9,315 – 13,703)	41.2	3,241 (2,787 – 3,751)	6,956 (5,811 – 8,309)	114.6
Pancreas	1,701 (1,368 – 1,992)	3,164 (2,542 – 3,917)	86.0	5,944 (5,178 – 6,773)	10,929 (9,135 – 13,027)	83.9	2,358 (1,885 – 2,867)	8,507 (6,896 – 10,343)	260.8
Prostate	12,995 (11,727 – 14,404)	15,411 (13,699 – 17,386)	18.6	181,651 (172,753 – 191,167)	206,641 (188,853 – 226,313)	13.8	115,017 (110,896 – 119,247)	246,939 (231,583 – 263,584)	114.7
Stomach	1,945 (1,555 – 2,327)	2,467 (1,986 – 3,053)	26.8	7,448 (6,581 – 8,400)	10,674 (9,034 – 12,596)	43.3	4,958 (4,219 – 5,754)	11,187 (9,415 – 13,195)	125.6
Testis	16,522 (15,334 – 17,758)	25,150 (22,231 – 28,455)	52.2	4,009 (3,680 – 4,371)	6,798 (6,009 – 7,697)	69.6	299 (237 – 368)	889 (734 – 1,084)	197.3
Thyroid	26,911 (24,551 – 29,304)	39,217 (32,183 – 47,920)	45.7	25,183 (23,258 – 27,220)	43,820 (38,151 – 50,379)	74.0	5,734 (5,008 – 6,525)	20,928 (17,693 – 24,808)	265.0
Unknown primary site	989 (724 – 1,302)	1,268 (914 – 1,738)	28.2	3,824 (3,181 – 4,540)	4,931 (3,789 – 6,375)	28.9	2,292 (1,804 – 2,838)	6,115 (4,805 – 7,754)	166.8
Uterus	6,446 (5,898 – 6,970)	9,897 (8,763 – 11,177)	53.5	25,266 (23,807 – 26,792)	39,682 (35,748 – 44,042)	57.1	12,735 (11,868 – 13,663)	33,702 (30,351 – 37,412)	164.6
Other cancers	33,400 (29,401 – 37,804)	45,812 (36,407 – 58,119)	37.2	46,955 (43,250 – 50,876)	65,458 (57,356 – 74,674)	39.4	29,061 (25,716 – 32,603)	66,375 (56,285 – 77,870)	128.4

B. 5-year prevalence

	Age <60 years			Age 60-79 years			Age 80+ years		
**Cancer type**	**Counts (95% UI) in 2025**	**Counts (95% UI) in 2050**	**Change %**	**Counts (95% UI) in 2025**	**Counts (95% UI) in 2050**	**Change %**	**Counts (95% UI) in 2025**	**Counts (95% UI) in 2050**	**Change %**
**All cancers**	**147,145 (133,685 – 162,654)**	**201,577 (174,433 – 234,964)**	**37.0**	**314,916 (293,905 – 337,796)**	**389,809 (350,064 – 435,400)**	**23.8**	**109,875 (101,613 – 118,913)**	**198,964 (178,816 – 221,973)**	**81.1**
Bladder	813 (675 – 975)	1,109 (895 – 1,370)	36.4	5,199 (4,694 – 5,768)	6,162 (5,154 – 7,377)	18.5	3,460 (3,086 – 3,882)	5,526 (4,612 – 6,615)	59.7
Brain	2,272 (1,866 – 2,781)	2,889 (2,235 – 3,773)	27.2	1,479 (1,282 – 1,700)	1,766 (1,443 – 2,148)	19.4	274 (224 – 334)	471 (376 – 586)	71.9
Breast	33,941 (32,629 – 35,310)	48,530 (45,439 – 51,838)	43.0	45,308 (44,028 – 46,623)	62,960 (59,536 – 66,587)	39.0	11,742 (11,239 – 12,264)	22,100 (21,197 – 23,043)	88.2
Cervix	2,879 (2,460 – 3,367)	3,720 (2,805 – 4,932)	29.2	920 (767 – 1,101)	1,404 (1,041 – 1,891)	52.6	128 (99 – 169)	228 (156 – 329)	78.1
Colorectum	12,591 (11,403 – 13,915)	21,252 (17,877 – 25,352)	68.8	31,204 (29,396 – 33,127)	31,900 (28,580 – 35,603)	2.2	15,084 (14,077 – 16,165)	20,602 (18,335 – 23,141)	36.6
Gallbladder and bile duct	299 (230 – 391)	447 (329 – 609)	49.5	1,275 (1,058 – 1,534)	1,732 (1,337 – 2,230)	35.8	544 (432 – 680)	1,056 (793 – 1,395)	94.1
Kidney	5,504 (4,810 – 6,332)	8,852 (7,284 – 10,796)	60.8	10,373 (9,477 – 11,353)	18,753 (15,801 – 22,278)	80.8	2,517 (2,210 – 2,868)	5,631 (4,746 – 6,672)	123.7
Larynx	348 (272 – 450)	422 (317 – 568)	21.3	1,355 (1,153 – 1,603)	1,410 (1,084 – 1,863)	4.1	367 (299 – 453)	476 (359 – 631)	29.7
Leukaemia	5,105 (4,386 – 5,990)	7,041 (5,847 – 8,632)	37.9	8,893 (8,315 – 9,514)	12,788 (11,634 – 14,073)	43.8	3,545 (3,230 – 3,893)	7,301 (6,534 – 8,156)	106.0
Lip, oral cavity and pharynx	5,558 (4,953 – 6,239)	6,989 (5,778 – 8,468)	25.7	9,634 (8,780 – 10,569)	11,400 (9,562 – 13,597)	18.3	2,281 (1,997 – 2,599)	4,690 (3,886 – 5,668)	105.6
Liver	1,281 (1,007 – 1,696)	1,986 (1,449 – 2,847)	55.0	3,968 (3,470 – 4,535)	5,259 (4,013 – 6,938)	32.5	789 (646 – 962)	2,020 (1,554 – 2,617)	156.0
Lung	3,560 (3,171 – 3,986)	4,855 (4,165 – 5,662)	36.4	18,032 (16,801 – 19,352)	21,485 (18,792 – 24,565)	19.1	6,311 (5,774 – 6,888)	10,411 (9,101 – 11,905)	65.0
Melanoma	21,491 (20,185 – 22,945)	25,818 (23,455 – 28,587)	20.1	37,133 (35,534 – 38,803)	35,413 (32,775 – 38,268)	-4.6	16,100 (15,216 – 17,029)	29,064 (26,864 – 31,441)	80.5
Multiple myeloma	1,346 (1,140 – 1,586)	2,043 (1,699 – 2,466)	51.8	5,481 (4,982 – 6,027)	8,054 (6,868 – 9,516)	46.9	2,007 (1,774 – 2,260)	4,246 (3,618 – 4,977)	111.6
Non-Hodgkin lymphoma	5,974 (5,293 – 6,790)	8,008 (6,695 – 9,655)	34.0	13,530 (12,455 – 14,700)	16,546 (14,220 – 19,249)	22.3	5,195 (4,676 – 5,773)	9,720 (8,284 – 11,398)	87.1
Oesophagus	487 (411 – 573)	647 (532 – 785)	32.9	2,093 (1,838 – 2,378)	2,627 (2,232 – 3,095)	25.5	694 (579 – 832)	1,243 (992 – 1,542)	79.1
Ovarian/tubal serous cancer	2,198 (1,839 – 2,634)	3,313 (2,410 – 4,605)	50.7	3,087 (2,782 – 3,424)	3,840 (3,214 – 4,594)	24.4	672 (575 – 784)	1,119 (915 – 1,368)	66.5
Pancreas	1,195 (1,018 – 1,399)	1,843 (1,534 – 2,205)	54.2	4,050 (3,647 – 4,484)	6,022 (5,173 – 7,004)	48.7	1,290 (1,133 – 1,459)	2,704 (2,304 – 3,160)	109.6
Prostate	8,603 (7,637 – 9,705)	11,146 (9,979 – 12,462)	29.6	70,689 (65,581 – 76,325)	85,930 (79,946 – 92,462)	21.6	21,743 (20,812 – 22,713)	40,064 (38,292 – 41,913)	84.3
Stomach	1,093 (932 – 1,281)	1,412 (1,187 – 1,684)	29.2	3,675 (3,347 – 4,041)	5,064 (4,384 – 5,848)	37.8	1,379 (1,225 – 1,548)	2,719 (2,340 – 3,143)	97.2
Testis	4,376 (3,979 – 4,808)	5,925 (5,184 – 6,765)	35.4	270 (228 – 322)	368 (303 – 449)	36.3	38 (26 – 52)	71 (53 – 99)	86.8
Thyroid	10,028 (9,004 – 11,180)	12,417 (10,060 – 15,364)	23.8	6,907 (6,236 – 7,659)	9,466 (8,136 – 11,028)	37.0	988 (824 – 1,193)	2,768 (2,192 – 3,497)	180.2
Unknown primary site	539 (426 – 686)	656 (485 – 881)	21.7	1,687 (1,439 – 1,966)	1,889 (1,473 – 2,422)	12.0	897 (758 – 1,055)	1,387 (1,083 – 1,771)	54.6
Uterus	3,534 (3,268 – 3,827)	5,170 (4,614 – 5,798)	46.3	8,530 (7,968 – 9,129)	12,121 (10,889 – 13,488)	42.1	1,853 (1,683 – 2,039)	3,745 (3,275 – 4,278)	102.1
Other cancers	12,130 (10,691 – 13,808)	15,087 (12,179 – 18,860)	24.4	20,144 (18,647 – 21,759)	25,450 (22,474 – 28,827)	26.3	9,977 (9,019 – 11,019)	19,602 (16,955 – 22,628)	96.5

C. 1-year prevalence

	Age <60 years			Age 60- 79 years			Age 80+ years		
**Cancer type**	**Counts (95% UI) in 2025**	**Counts (95% UI) in 2050**	**Change %**	**Counts (95% UI) in 2025**	**Counts (95% UI) in 2050**	**Change %**	**Counts (95% UI) in 2025**	**Counts (95% UI) in 2050**	**Change %**
**All cancers**	**37,273 (33,880 – 41,177)**	**50,432 (43,904 – 58,364)**	**35.3**	**78,194 (72,981 – 83,881)**	**96,718 (87,051 – 107,764)**	**23.7**	**30,420 (28,272 – 32,766)**	**55,886 (50,615 – 61,861)**	**83.7**
Bladder	241 (205 – 283)	329 (272 – 395)	36.5	1,460 (1,326 – 1,611)	1,734 (1,466 – 2,056)	18.8	1,103 (1,001 – 1,216)	1,788 (1,528 – 2,091)	62.1
Brain	694 (587 – 824)	879 (705 – 1,105)	26.7	705 (633 – 785)	833 (709 – 978)	18.2	163 (141 – 188)	285 (244 – 336)	74.8
Breast	8,131 (7,812 – 8,463)	11,479 (10,759 – 12,247)	41.2	9,505 (9,229 – 9,788)	13,128 (12,438 – 13,858)	38.1	2,589 (2,484 – 2,699)	4,868 (4,687 – 5,055)	88.0
Cervix	663 (560 – 781)	860 (651 – 1,133)	29.7	215 (180 – 257)	334 (251 – 443)	55.3	39 (31 – 50)	72 (52 – 100)	84.6
Colorectum	3,304 (2,997 – 3,651)	5,262 (4,471 – 6,214)	59.3	7,699 (7,235 – 8,191)	8,041 (7,226 – 8,947)	4.4	3,774 (3,528 – 4,039)	5,304 (4,763 – 5,908)	40.5
Gallbladder and bile duct	117 (95 – 144)	172 (134 – 221)	47.0	511 (440 – 596)	688 (554 – 856)	34.6	283 (240 – 333)	557 (453 – 687)	96.8
Kidney	1,399 (1,224 – 1,606)	2,209 (1,830 – 2,677)	57.9	2,399 (2,189 – 2,629)	4,265 (3,617 – 5,035)	77.8	606 (536 – 684)	1,356 (1,164 – 1,580)	123.8
Larynx	102 (82 – 129)	126 (95 – 164)	23.5	334 (289 – 391)	357 (278 – 458)	6.9	94 (80 – 115)	131 (102 – 167)	39.4
Leukaemia	1,262 (1,103 – 1,460)	1,725 (1,465 – 2,071)	36.7	2,316 (2,182 – 2,461)	3,292 (3,022 – 3,591)	42.1	1,098 (1,020 – 1,183)	2,275 (2,086 – 2,482)	107.2
Lip, oral cavity and pharynx	1,415 (1,259 – 1,588)	1,773 (1,475 – 2,134)	25.3	2,288 (2,081 – 2,518)	2,707 (2,288 – 3,207)	18.3	581 (512 – 657)	1,216 (1,025 – 1,443)	109.3
Liver	479 (389 – 604)	718 (543 – 981)	49.9	1,456 (1,292 – 1,641)	1,863 (1,450 – 2,406)	28.0	386 (332 – 448)	979 (804 – 1,194)	153.6
Lung	1,350 (1,225 – 1,490)	1,812 (1,585 – 2,076)	34.2	6,538 (6,120 – 6,985)	7,711 (6,815 – 8,728)	17.9	2,767 (2,570 – 2,977)	4,599 (4,111 – 5,149)	66.2
Melanoma	4,918 (4,616 – 5,256)	5,856 (5,332 – 6,466)	19.1	8,318 (7,950 – 8,702)	7,865 (7,299 – 8,477)	-5.4	3,751 (3,553 – 3,960)	6,698 (6,236 – 7,191)	78.6
Multiple myeloma	369 (318 – 426)	548 (464 – 651)	48.5	1,409 (1,292 – 1,538)	2,024 (1,745 – 2,362)	43.6	603 (547 – 662)	1,272 (1,123 – 1,445)	110.9
Non-Hodgkin lymphoma	1,463 (1,298 – 1,657)	1,938 (1,635 – 2,316)	32.5	3,265 (2,999 – 3,557)	3,946 (3,407 – 4,565)	20.9	1,370 (1,242 – 1,514)	2,553 (2,212 – 2,947)	86.4
Oesophagus	199 (176 – 227)	267 (227 – 311)	34.2	783 (706 – 871)	983 (855 – 1,128)	25.5	324 (285 – 367)	589 (501 – 690)	81.8
Ovarian/tubal serous cancer	576 (486 – 684)	845 (628 – 1,147)	46.7	818 (742 – 903)	1,012 (857 – 1,195)	23.7	266 (236 – 301)	455 (389 – 532)	71.1
Pancreas	472 (418 – 536)	705 (608 – 819)	49.4	1,845 (1,696 – 2,004)	2,662 (2,335 – 3,033)	44.3	860 (788 – 939)	1,824 (1,627 – 2,044)	112.1
Prostate	2,479 (2,195 – 2,800)	3,266 (2,937 – 3,637)	31.7	15,445 (14,346 – 16,654)	19,190 (17,975 – 20,508)	24.2	4,667 (4,477 – 4,865)	9,038 (8,671 – 9,420)	93.7
Stomach	367 (324 – 418)	477 (414 – 550)	30.0	1,173 (1,088 – 1,269)	1,617 (1,426 – 1,832)	37.9	513 (474 – 555)	1,039 (937 – 1,152)	102.5
Testis	927 (840 – 1,023)	1,237 (1,081 – 1,408)	33.4	50 (42 – 60)	69 (57 – 83)	38.0	9 (6 – 11)	16 (12 – 24)	77.8
Thyroid	2,259 (2,014 – 2,538)	2,725 (2,208 – 3,370)	20.6	1,449 (1,301 – 1,617)	1,915 (1,652 – 2,224)	32.2	208 (175 – 252)	570 (458 – 710)	174.0
Unknown primary site	206 (171 – 250)	250 (195 – 320)	21.4	745 (653 – 848)	832 (670 – 1,037)	11.7	651 (575 – 736)	1,025 (839 – 1,255)	57.5
Uterus	934 (865 – 1,010)	1,351 (1,211 – 1,507)	44.6	1,898 (1,770 – 2,035)	2,675 (2,412 – 2,964)	40.9	436 (400 – 476)	883 (784 – 994)	102.5
Other cancers	2,947 (2,621 – 3,329)	3,623 (2,979 – 4,444)	22.9	5,570 (5,200 – 5,970)	6,975 (6,247 – 7,793)	25.2	3,279 (3,039 – 3,539)	6,494 (5,807 – 7,265)	98.0

The uncertainty intervals (UIs) for the estimated prevalence were estimated based on the UIs of the number of new cases and survival rates.

Table S8. Estimated number of people living with cancer (cancers not yet cured) and living beyond cancer (potentially cured) in Australia, 2025 to 2050

A. Persons

Cancer type	People living beyond cancer (potentially cured)[a]			People living with cancer (cancers not yet cured)[b]		
	Counts (95% UI) in 2025	Counts (95% UI) in 2050	Change %	Counts (95% UI) in 2025	Counts (95% UI) in 2050	Change %
All cancers	871,643 (770,147 – 966,694)	1,467,652 (1,230,784 – 1,708,797)	68·4	794,377 (783,678 – 818,206)	1,149,364 (1,103,165 – 1,238,377)	44·7
Bladder	5,590 (544 – 6,858)	8,990 (412 – 11,348)	60·8	17,233 (19,550 – 18,823)	23,633 (26,607 – 28,060)	37·1
Brain	325 (0 – 1,716)	1,070 (0 – 3,925)	229·2	8,709 (7,177 – 9,412)	12,041 (9,897 – 13,393)	38·3
Breast	207,118 (201,022 – 213,275)	351,545 (334,298 – 369,707)	69·7	90,991 (87,896 – 94,197)	133,590 (126,172 – 141,468)	46·8
Cervix	9,279 (8,336 – 10,942)	13,221 (10,464 – 17,832)	42·5	6,497 (5,599 – 6,847)	8,802 (6,586 – 10,677)	35·5
Colorectum	57,267 (48,230 – 67,501)	89,570 (72,914 – 110,048)	56·4	116,726 (115,121 – 117,659)	147,483 (136,271 – 159,009)	26·3
Gallbladder and bile duct	869 (387 – 1,504)	2,059 (1,132 – 3,554)	136·9	3,061 (2,508 – 3,501)	4,719 (3,681 – 5,791)	54·2
Kidney	20,841 (7,490 – 28,507)	55,998 (20,020 – 77,760)	168·7	28,067 (36,444 – 25,759)	52,103 (71,054 – 50,755)	85·6
Larynx	0 (0 – 186)	0 (0 – 48)		5,871 (4,818 – 6,742)	5,330 (4,002 – 7,152)	-9·2
Leukaemia	11,059 (7,137 – 13,589)	30,301 (21,396 – 36,701)	174.0	35,535 (34,558 – 38,361)	57,557 (55,350 – 64,524)	62.0
Lip, oral cavity and pharynx	4,470 (1,525 – 12,444)	3,901 (982 – 17,461)	-12·7	49,581 (47,574 – 46,612)	73,145 (64,070 – 73,883)	47·5
Liver	794 (287 – 1,403)	4,970 (1,517 – 8,067)	525·9	9,028 (7,655 – 10,743)	16,441 (14,126 – 21,590)	82·1
Lung	1,375 (0 – 3,139)	6,248 (0 – 13,078)	354·4	47,245 (44,101 – 50,272)	79,036 (73,647 – 85,579)	67·3
Melanoma	167,017 (153,699 – 181,334)	231,614 (206,759 – 260,020)	38·7	82,875 (84,861 – 80,185)	100,295 (99,735 – 100,086)	21.0
Multiple myeloma	0 (0 – 595)	0 (0 – 1,884)		15,420 (13,252 – 17,230)	33,636 (27,745 – 38,932)	118·1
Non-Hodgkin lymphoma	23,160 (4,008 – 36,792)	41,656 (8,299 – 69,608)	79·9	44,150 (57,234 – 37,034)	65,477 (83,223 – 56,036)	48·3
Oesophagus	578 (0 – 1,105)	1,761 (0 – 2,988)	204·7	5,641 (5,031 – 6,421)	8,644 (8,253 – 9,986)	53·2
Ovarian/tubal serous cancer	4,891 (3,448 – 5,633)	10,135 (7,078 – 12,515)	107·2	11,114 (10,454 – 12,714)	16,084 (13,697 – 20,791)	44·7
Pancreas	727 (322 – 1,436)	5,541 (3,297 – 8,369)	662·2	9,276 (8,109 – 10,196)	17,059 (15,276 – 18,918)	83·9
Prostate	226,913 (218,557 – 235,536)	355,894 (328,296 – 386,304)	56·8	82,750 (76,819 – 89,282)	113,097 (105,839 – 120,979)	36·7
Stomach	3,933 (2,722 – 5,832)	8,459 (5,974 – 12,157)	115·1	10,418 (9,633 – 10,649)	15,869 (14,461 – 16,687)	52·3
Testis	17,037 (15,828 – 18,295)	27,706 (24,507 – 31,339)	62·6	3,793 (3,423 – 4,202)	5,131 (4,467 – 5,897)	35·3
Thyroid	42,278 (38,926 – 45,612)	82,515 (70,345 – 97,007)	95·2	15,550 (13,891 – 17,437)	21,450 (17,682 – 26,100)	37·9
Unknown primary site	1,701 (1,144 – 2,790)	4,926 (3,719 – 7,339)	189·6	5,404 (4,565 – 5,890)	7,388 (5,789 – 8,528)	36·7
Uterus	23,968 (20,621 – 25,438)	51,935 (44,036 – 57,467)	116·7	20,479 (20,952 – 21,987)	31,346 (30,826 – 35,164)	53·1
Other cancers	40,453 (35,914 – 45,232)	77,637 (65,339 – 92,271)	91·9	68,963 (62,453 – 76,051)	100,008 (84,709 – 118,392)	45.0

B. Males

Cancer type	People living beyond cancer (potentially cured)[a]			People living with cancer (cancers not yet cured)[b]		
	Counts (95% UI) in 2025	Counts (95% UI) in 2050	Change %	Counts (95% UI) in 2025	Counts (95% UI) in 2050	Change %
All cancers	421,294 (365,648 – 480,142)	708,528 (574,444 – 850,411)	68·2	441,028 (437,722 – 444,706)	626,438 (615,671 – 652,160)	42
Bladder	4,435 (0 – 4,953)	8,094 (0 – 9,460)	82·5	13,913 (16,486 – 15,358)	19,483 (23,354 – 23,069)	40
Brain	325 (0 – 925)	1,070 (0 – 2,700)	229·2	5,050 (4,331 – 5,610)	7,512 (6,578 – 8,520)	48·8
Colorectum	30,028 (25,310 – 35,376)	50,706 (41,414 – 62,184)	68·9	64,687 (63,755 – 65,287)	80,879 (74,222 – 87,986)	25
Gallbladder and bile duct	465 (243 – 787)	867 (474 – 1,596)	86·5	1,625 (1,290 – 1,886)	2,474 (1,802 – 3,159)	52·2
Kidney	12,348 (0 – 18,963)	31,766 (0 – 48,395)	157·3	19,025 (28,498 – 15,473)	35,595 (57,851 – 30,118)	87·1
Larynx	0 (0 – 0)	0 (0 – 0)		5,207 (4,433 – 6,038)	4,763 (3,710 – 6,088)	-8·5
Leukaemia	6,966 (3,665 – 8,806)	17,907 (10,875 – 22,024)	157·1	21,161 (21,831 – 22,129)	33,170 (34,900 – 35,251)	56·8
Lip, oral cavity and pharynx	0 (0 – 3,797)	0 (0 – 7,584)		38,506 (35,413 – 37,834)	57,995 (49,998 – 59,689)	50·6
Liver	516 (287 – 850)	2,333 (1,517 – 3,565)	352·1	6,783 (5,802 – 7,900)	11,602 (9,038 – 14,888)	71
Lung	770 (0 – 1,327)	2,782 (0 – 4,603)	261·3	22,766 (21,343 – 24,531)	37,491 (34,944 – 41,753)	64·7
Melanoma	80,054 (70,633 – 90,453)	120,315 (104,464 – 138,733)	50·3	57,116 (60,545 – 52,882)	68,986 (71,095 – 65,812)	20·8
Multiple myeloma	0 (0 – 595)	0 (0 – 1,884)		8,692 (7,601 – 9,282)	16,914 (14,674 – 17,569)	94·6
Non-Hodgkin lymphoma	7,153 (1,714 – 19,258)	16,150 (3,942 – 39,448)	125·8	30,335 (32,459 – 21,757)	45,554 (49,164 – 32,365)	50·2
Oesophagus	578 (0 – 996)	1,761 (0 – 2,574)	204·7	3,927 (3,770 – 4,337)	5,516 (5,900 – 6,319)	40·5
Pancreas	412 (250 – 809)	2,111 (1,520 – 3,386)	412·4	4,776 (4,148 – 5,198)	8,243 (6,963 – 9,151)	72·6
Prostate	226,913 (218,557 – 235,536)	355,894 (328,296 – 386,304)	56·8	82,750 (76,819 – 89,282)	113,097 (105,839 – 120,979)	36·7
Stomach	2,228 (1,821 – 3,231)	3,192 (2,588 – 4,788)	43·3	6,579 (5,855 – 6,783)	9,246 (7,955 – 9,809)	40·5
Testis	17,037 (15,828 – 18,295)	27,706 (24,507 – 31,339)	62·6	3,793 (3,423 – 4,202)	5,131 (4,467 – 5,897)	35·3
Thyroid	8,690 (7,635 – 9,695)	22,159 (17,964 – 27,399)	155	5,089 (4,408 – 5,889)	8,092 (6,390 – 10,280)	59
Unknown primary site	874 (641 – 1,428)	3,159 (2,564 – 4,509)	261·4	3,476 (2,920 – 3,815)	4,925 (3,817 – 5,700)	41·7
Other cancers	21,502 (19,064 – 24,062)	40,556 (34,319 – 47,936)	88·6	35,772 (32,592 – 39,233)	49,770 (43,010 – 57,758)	39·1

C. Females

Cancer type	People living beyond cancer (potentially cured)[a]			People living with cancer (cancers not yet cured)[b]		
	Counts (95% UI) in 2025	Counts (95% UI) in 2050	Change %	Counts (95% UI) in 2025	Counts (95% UI) in 2050	Change %
All cancers	450,349 (404,499 – 486,552)	759,124 (656,340 – 858,386)	68·6	353,349 (345,956 – 373,500)	522,926 (487,494 – 586,217)	48.0
Bladder	1,155 (544 – 1,905)	896 (412 – 1,888)	-22·4	3,320 (3,064 – 3,465)	4,150 (3,253 – 4,991)	25.0
Brain	0 (0 – 791)	0 (0 – 1,225)		3,659 (2,846 – 3,802)	4,529 (3,319 – 4,873)	23·8
Breast	207,118 (201,022 – 213,275)	351,545 (334,298 – 369,707)	69·7	90,991 (87,896 – 94,197)	133,590 (126,172 – 141,468)	46·8
Cervix	9,279 (8,336 – 10,942)	13,221 (10,464 – 17,832)	42·5	6,497 (5,599 – 6,847)	8,802 (6,586 – 10,677)	35·5
Colorectum	27,239 (22,920 – 32,125)	38,864 (31,500 – 47,864)	42·7	52,039 (51,366 – 52,372)	66,604 (62,049 – 71,023)	28.0
Gallbladder and bile duct	404 (144 – 717)	1,192 (658 – 1,958)	195.0	1,436 (1,218 – 1,615)	2,245 (1,879 – 2,632)	56·3
Kidney	8,493 (7,490 – 9,544)	24,232 (20,020 – 29,365)	185·3	9,042 (7,946 – 10,286)	16,508 (13,203 – 20,637)	82·6
Larynx	0 (0 – 186)	0 (0 – 48)		664 (385 – 704)	567 (292 – 1,064)	-14·6
Leukaemia	4,093 (3,472 – 4,783)	12,394 (10,521 – 14,677)	202·8	14,374 (12,727 – 16,232)	24,387 (20,450 – 29,273)	69·7
Lip, oral cavity and pharynx	4,470 (1,525 – 8,647)	3,901 (982 – 9,877)	-12·7	11,075 (12,161 – 8,778)	15,150 (14,072 – 14,194)	36·8
Liver	278 (0 – 553)	2,637 (0 – 4,502)	848·6	2,245 (1,853 – 2,843)	4,839 (5,088 – 6,702)	115·5
Lung	605 (0 – 1,812)	3,466 (0 – 8,475)	472·9	24,479 (22,758 – 25,741)	41,545 (38,703 – 43,826)	69·7
Melanoma	86,963 (83,066 – 90,881)	111,299 (102,295 – 121,287)	28.0	25,759 (24,316 – 27,303)	31,309 (28,640 – 34,274)	21·5
Multiple myeloma	0 (0 – 0)	0 (0 – 0)		6,728 (5,651 – 7,948)	16,722 (13,071 – 21,363)	148·5
Non-Hodgkin lymphoma	16,007 (2,294 – 17,534)	25,506 (4,357 – 30,160)	59·3	13,815 (24,775 – 15,277)	19,923 (34,059 – 23,671)	44·2
Oesophagus	0 (0 – 109)	0 (0 – 414)		1,714 (1,261 – 2,084)	3,128 (2,353 – 3,667)	82·5
Ovarian/tubal serous cancer	4,891 (3,448 – 5,633)	10,135 (7,078 – 12,515)	107·2	11,114 (10,454 – 12,714)	16,084 (13,697 – 20,791)	44·7
Pancreas	315 (72 – 627)	3,430 (1,777 – 4,983)	988·9	4,500 (3,961 – 4,998)	8,816 (8,313 – 9,767)	95·9
Stomach	1,705 (901 – 2,601)	5,267 (3,386 – 7,369)	208·9	3,839 (3,778 – 3,866)	6,623 (6,506 – 6,878)	72·5
Thyroid	33,588 (31,291 – 35,917)	60,356 (52,381 – 69,608)	79·7	10,461 (9,483 – 11,548)	13,358 (11,292 – 15,820)	27·7
Unknown primary site	827 (503 – 1,362)	1,767 (1,155 – 2,830)	113·7	1,928 (1,645 – 2,075)	2,463 (1,972 – 2,828)	27·7
Uterus	23,968 (20,621 – 25,438)	51,935 (44,036 – 57,467)	116·7	20,479 (20,952 – 21,987)	31,346 (30,826 – 35,164)	53·1
Other cancers	18,951 (16,850 – 21,170)	37,081 (31,020 – 44,335)	95·7	33,191 (29,861 – 36,818)	50,238 (41,699 – 60,634)	51·4

a. People living beyond cancer (potentially cured) refers to people with a past cancer diagnosis whose time since initial diagnosis reached or exceeded the estimated “time-to-cure” (at which 5-year conditional relative survival reaches 95%, or exceeds 90% and plateaus).

b. People living with cancer (cancers not yet cured) refers to people with a past cancer diagnosis, requiring initial treatment or ongoing care for cancer.

The uncertainty intervals (UIs) for the estimated prevalence were estimated based on the UIs of the number of new cases and survival rates.

Figure S9. Change in rank of cancer types sorted by decreasing cancer prevalence over the period 2025-2050, Australia, separately for all persons and by sex

A. Males

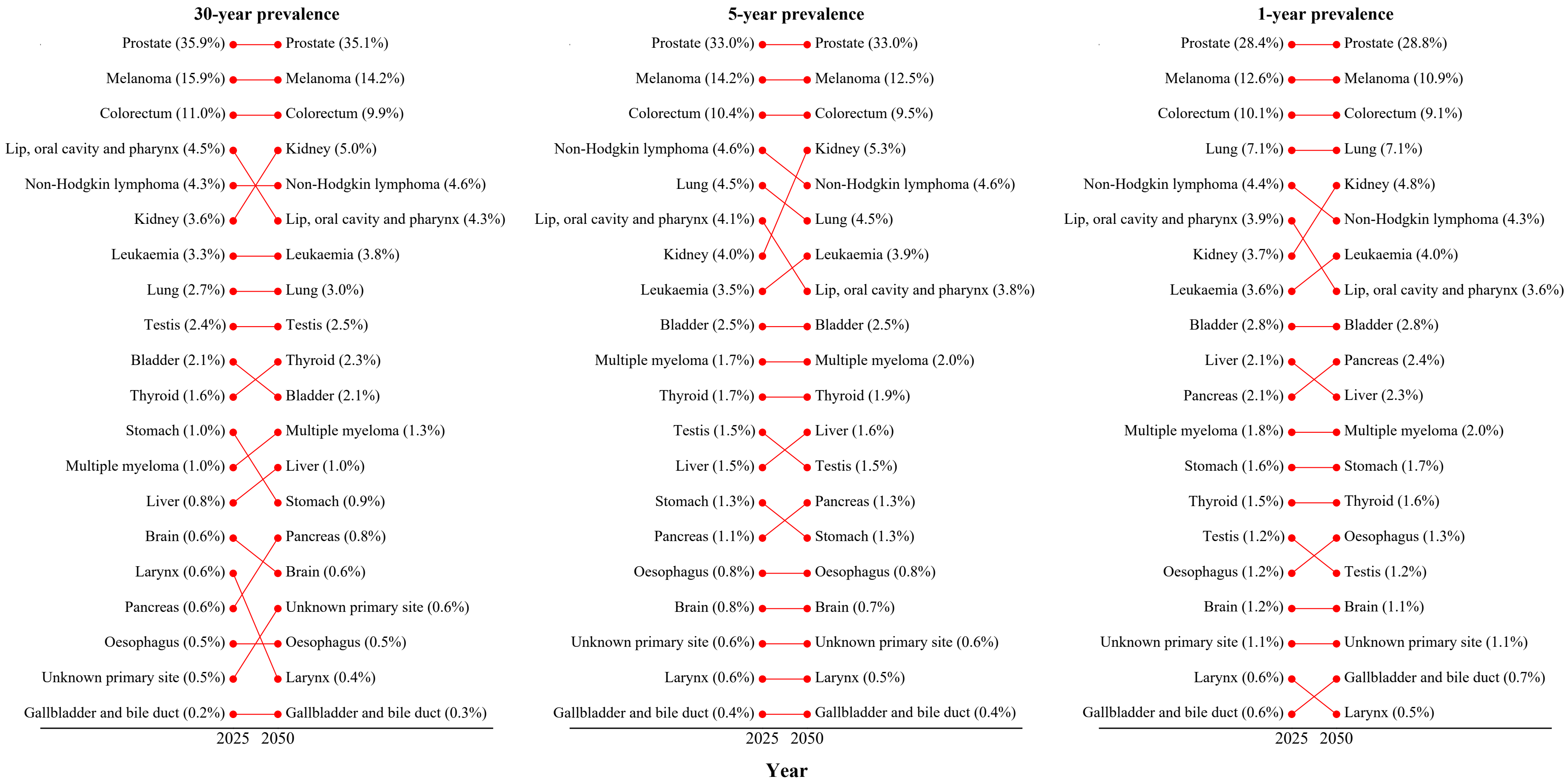

B. Females

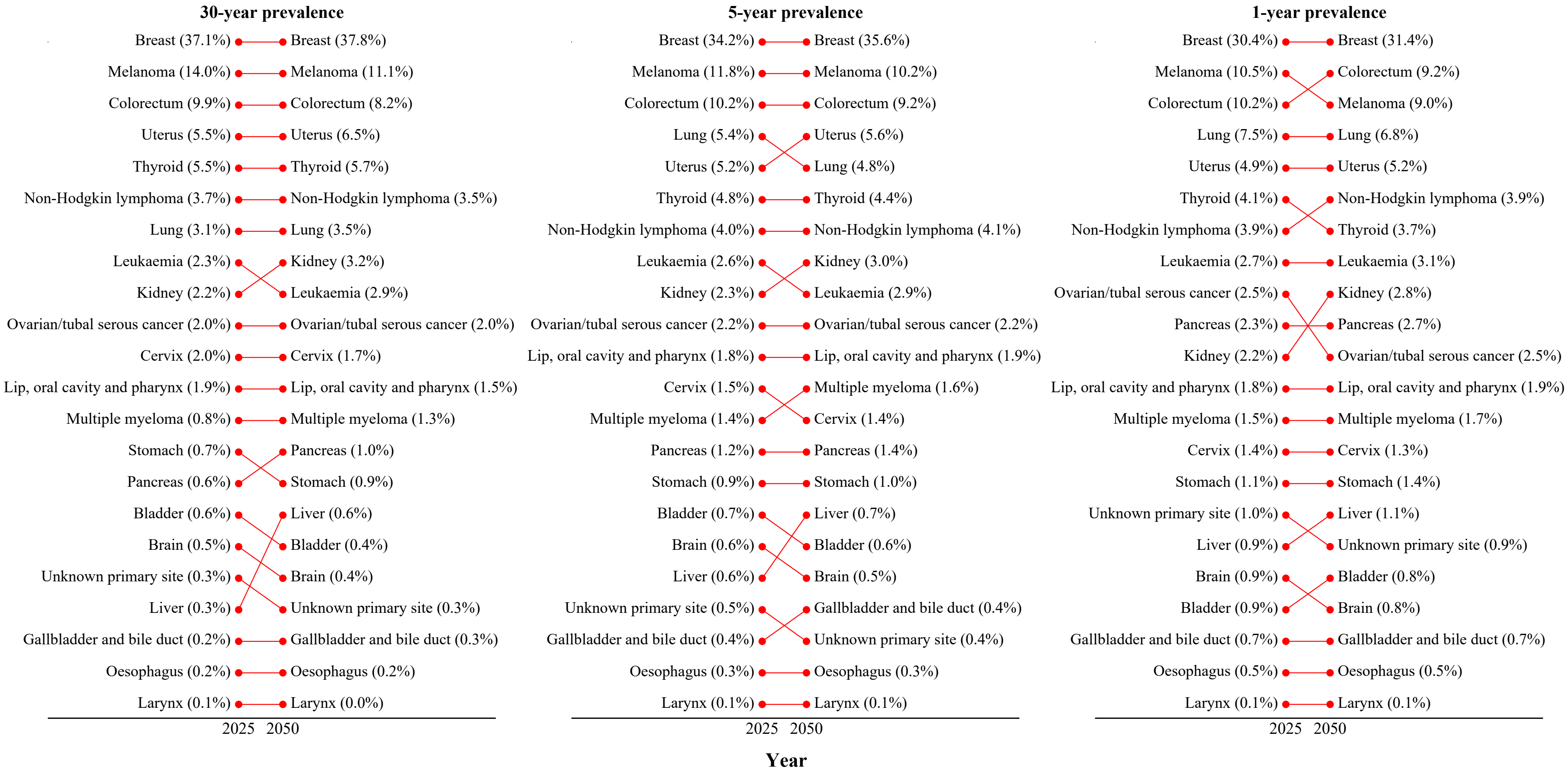

Percentages are of the total cancer prevalence.

Figure S10. Estimated cancer prevalence (95% uncertainty interval) by sex in Australia, 2025 – 2050

A. 30-year prevalence

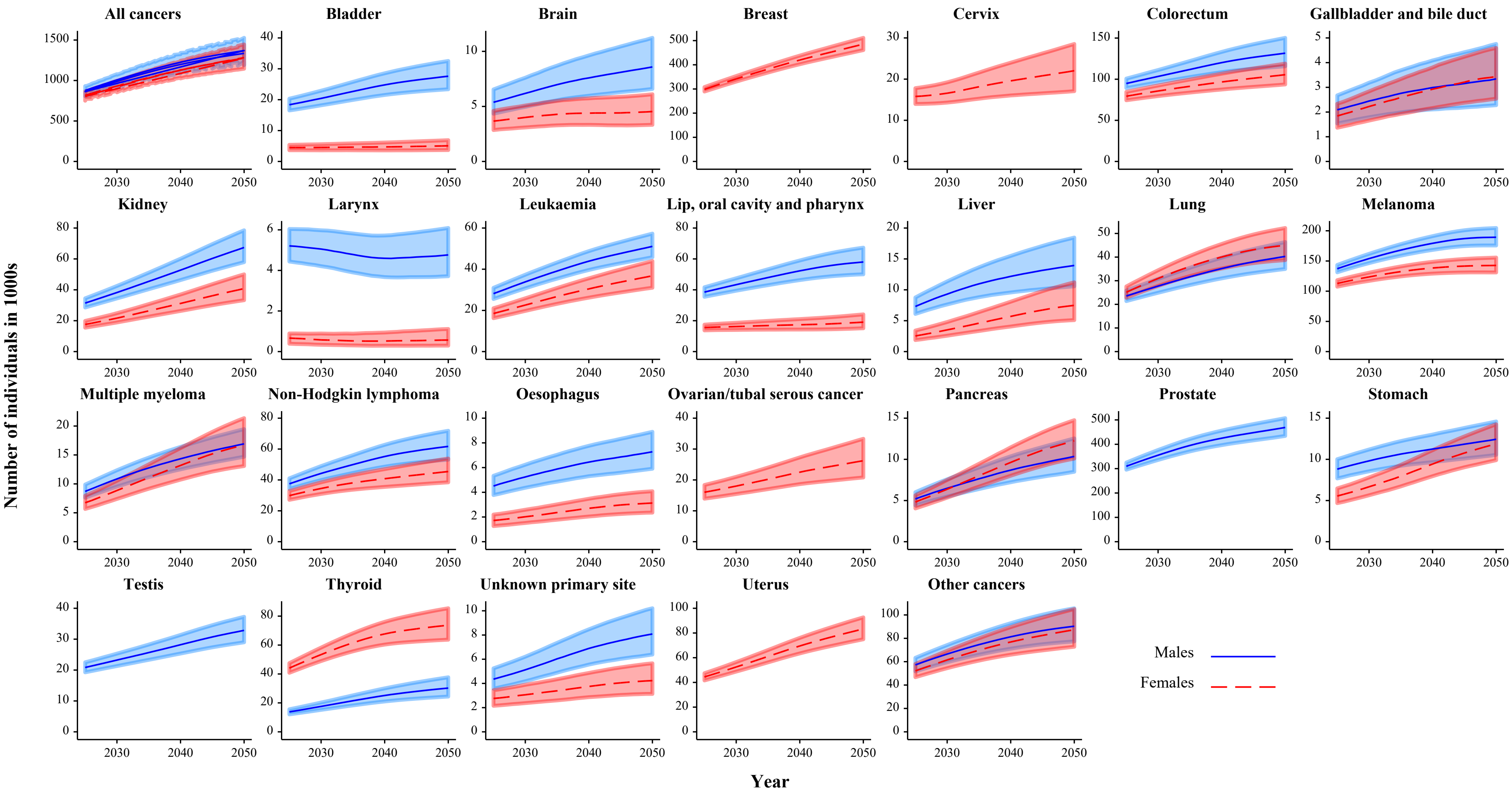

B. 5-year prevalence

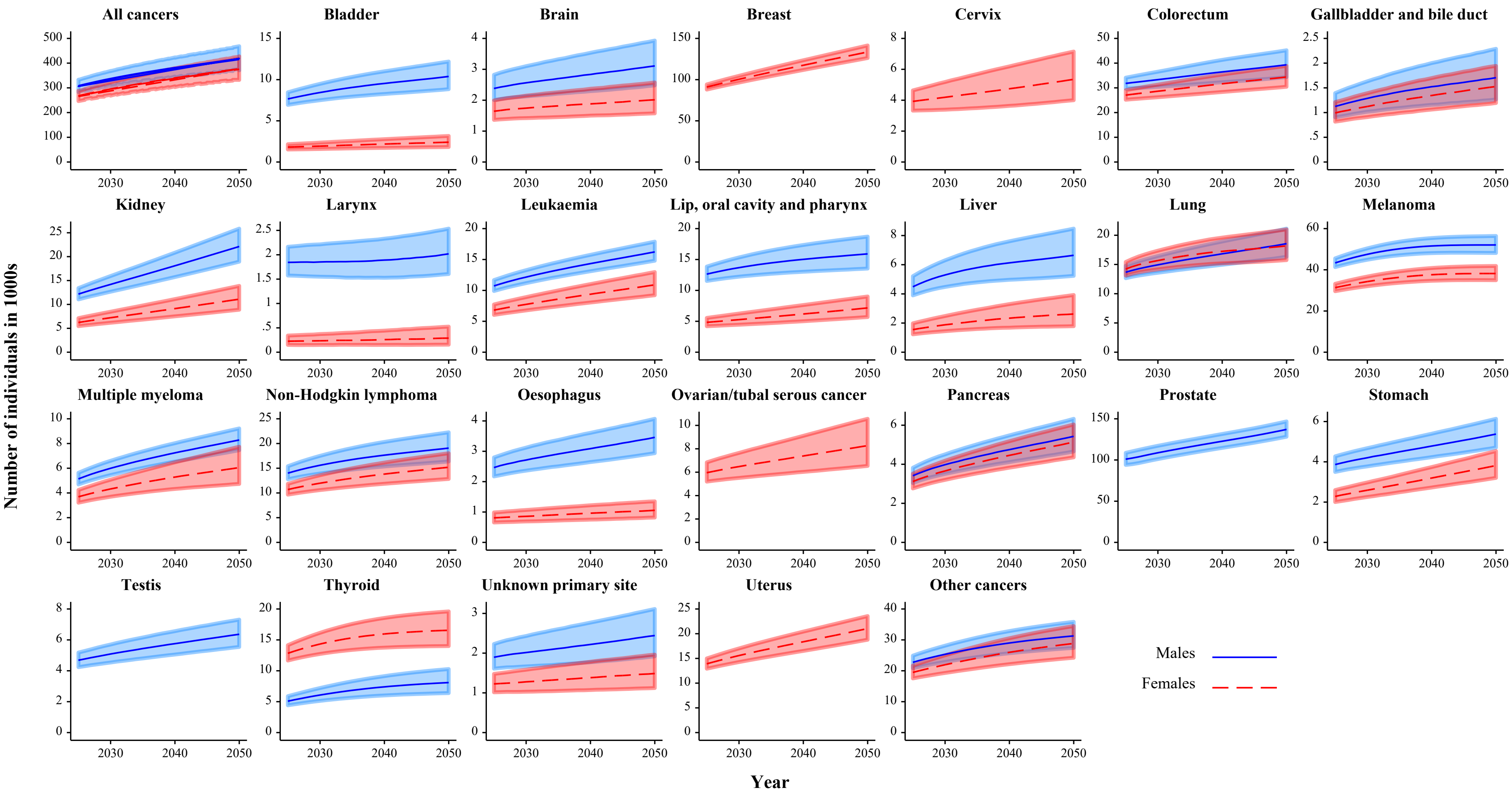

C. 1-year prevalence

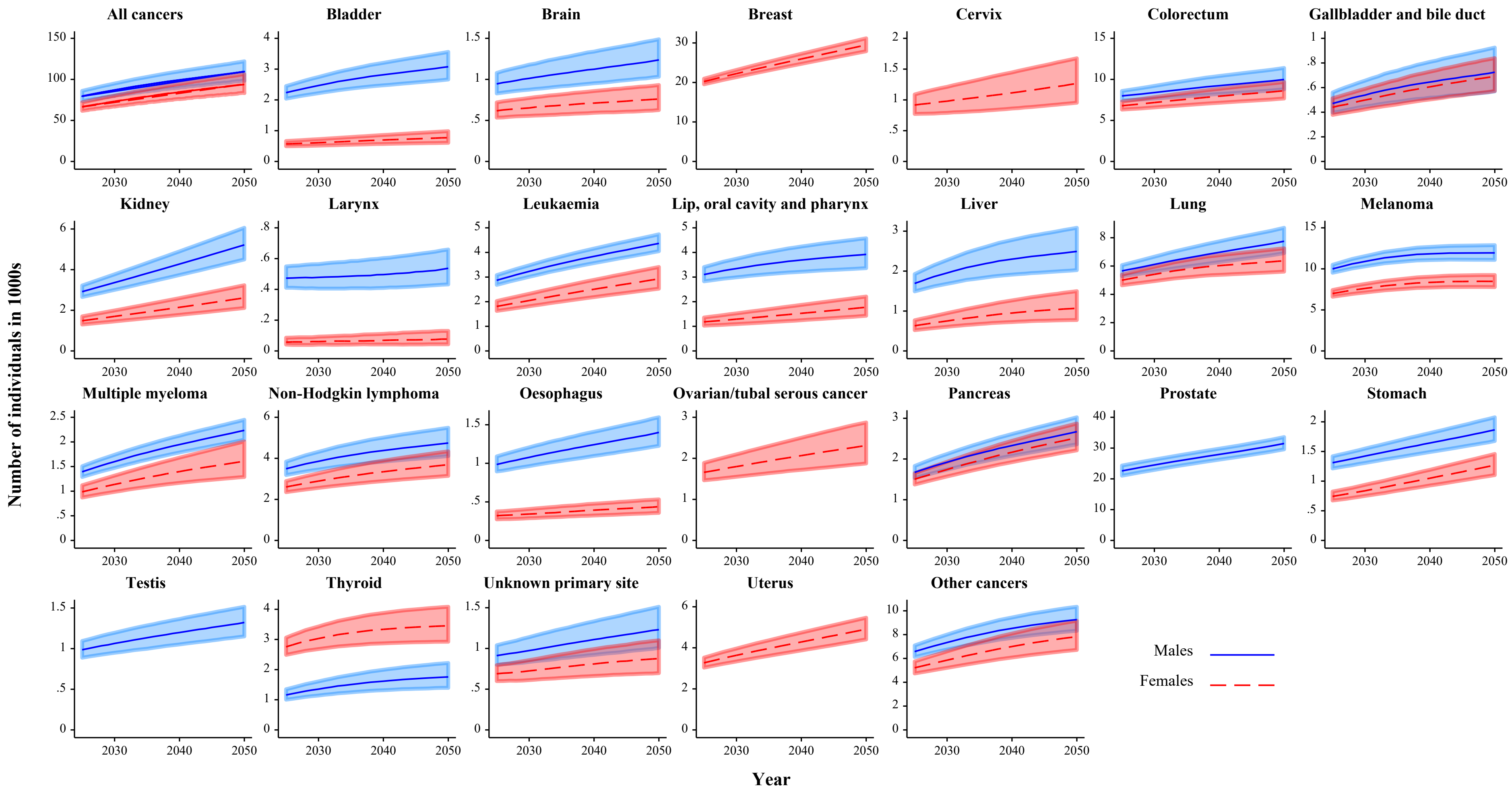

Shaded areas represent the 95% uncertainty interval (UIs). The UIs for the estimated prevalence were estimated based on the UIs of the number of new cases and survival rates.

Figure S11. Estimated cancer prevalence by age group in Australia, 2025 – 2050

A. 30-year prevalence

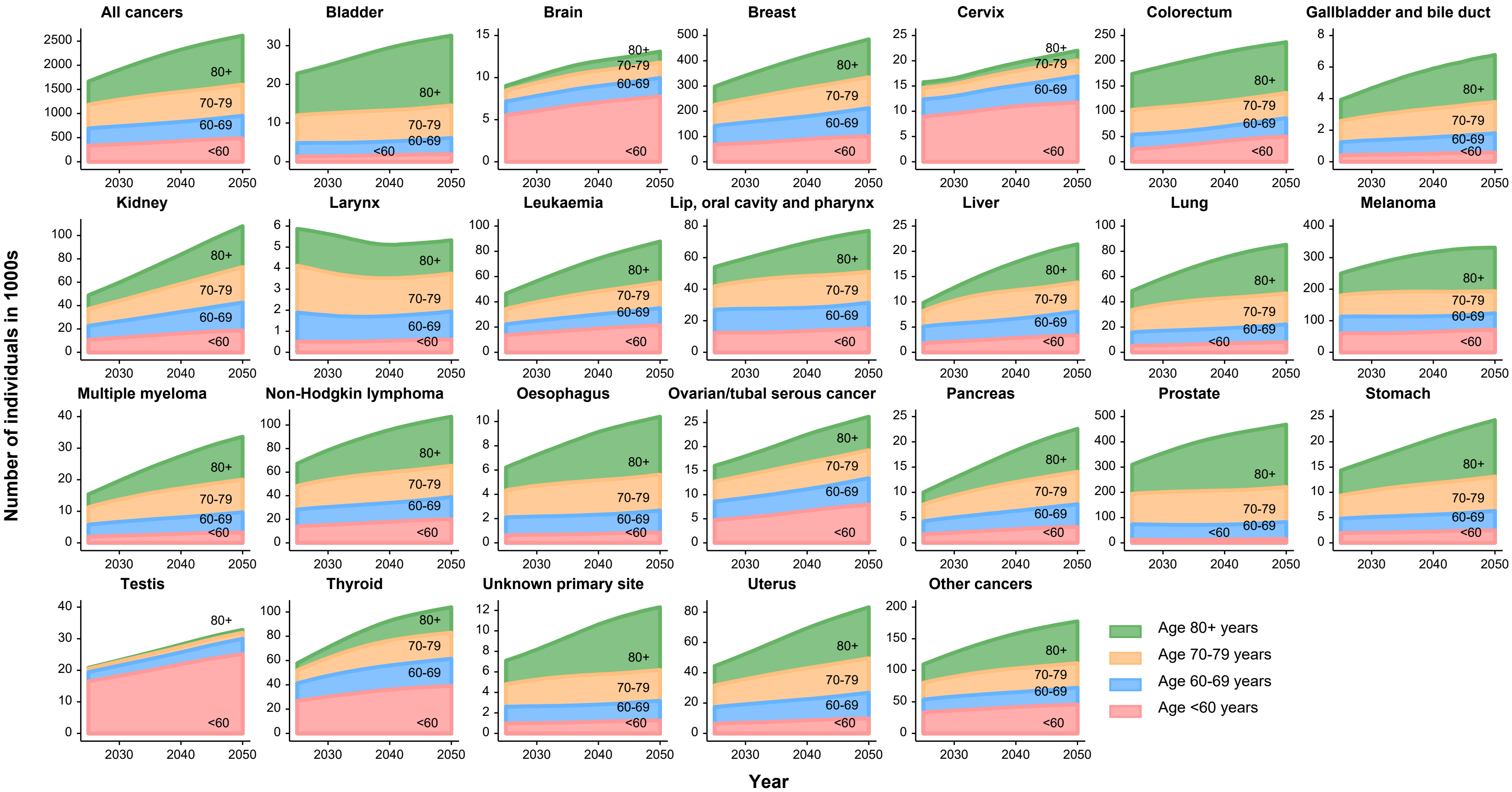

B. 5-year prevalence

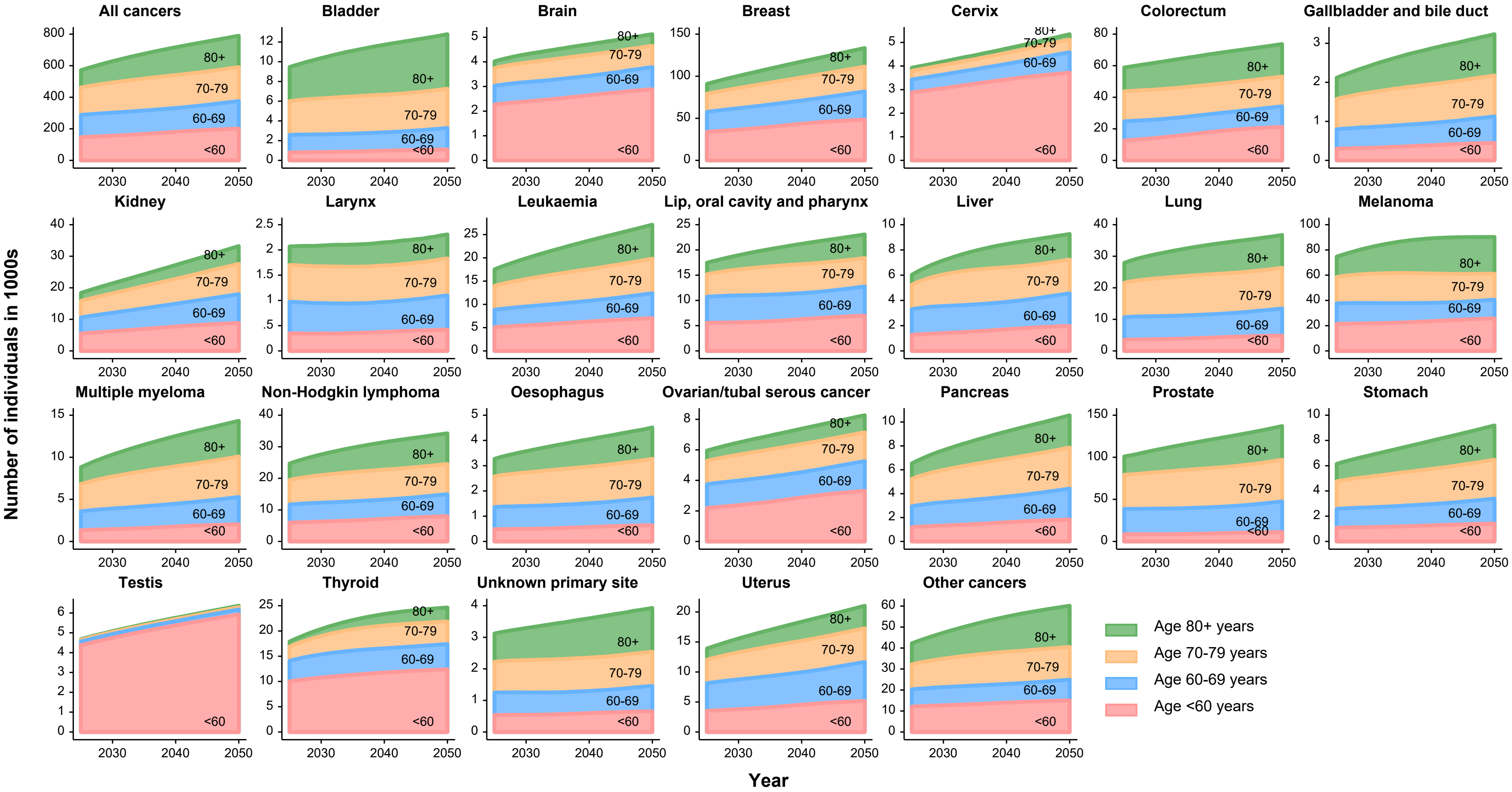

C. 1-year prevalence

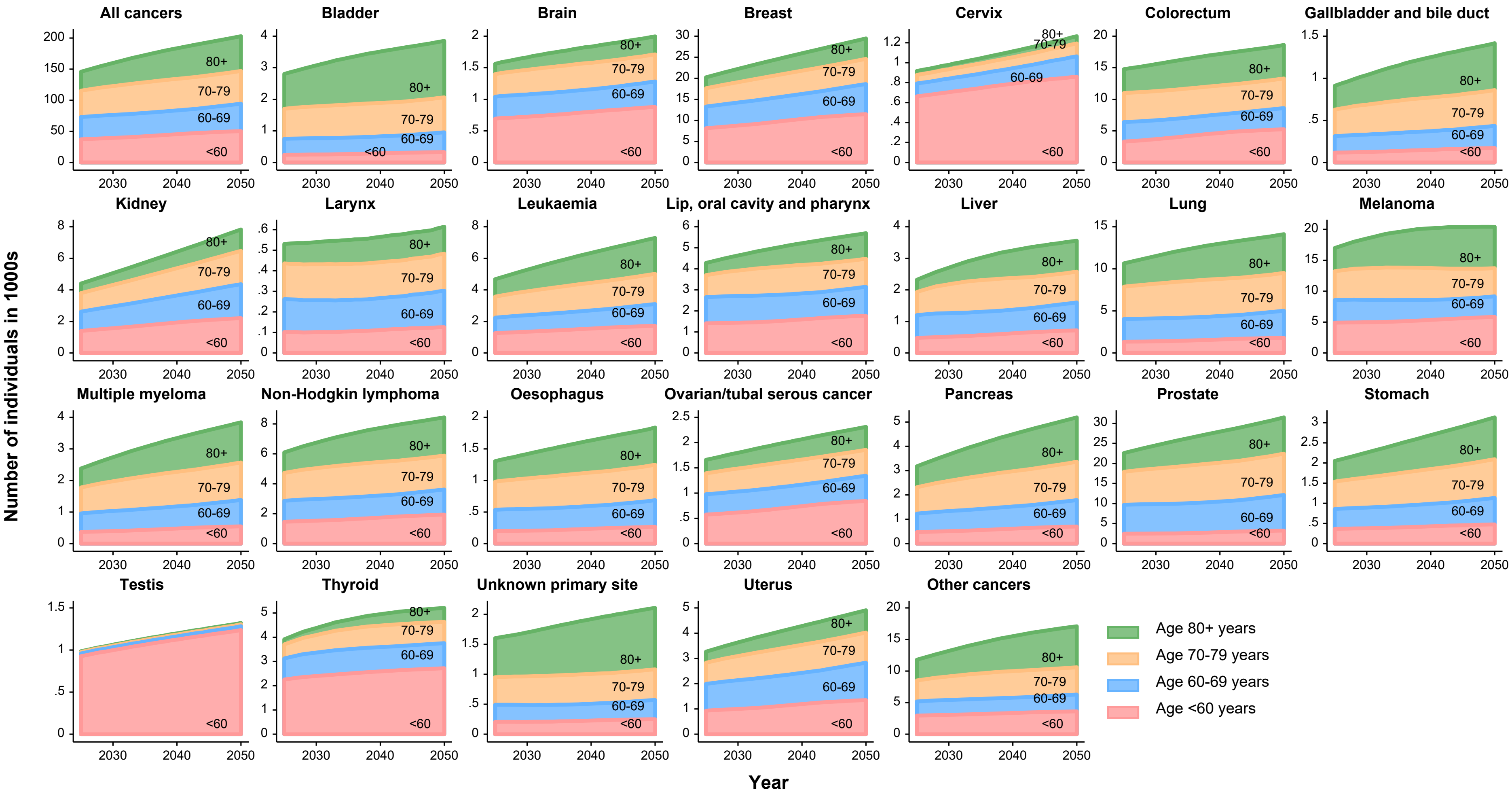

Figure S12. People living with cancer (cancers not yet cured) and living beyond cancer (potentially cured) ranked by estimated 30-year cancer prevalence in 2050, separately for males and females

A. Males

Cancer type	Prevalence	Cure proportion %
All males	1,334,966	53.1 (43.0-63.7)
Prostate	468,991	75.9 (70.0-82.4)
Melanoma	189,301	63.6 (55.2-73.3)
Colorectum	131,585	38.5 (31.5-47.3)
Kidney	67,361	47.2 (0.0-71.8)
Non-Hodgkin lymphoma	61,704	26.2 (6.4-63.9)
Lip, oral cavity and pharynx	57,995	0.0 (0.0-13.1)
Leukaemia	51,077	35.1 (21.3-43.1)
Lung	40,273	6.9 (0.0-11.4)
Testis	32,837	84.4 (74.6-95.4)
Thyroid	30,251	73.3 (59.4-90.6)
Bladder	27,577	29.4 (0.0-34.3)
Multiple myeloma	16,914	0.0 (0.0-11.1)
Liver	13,935	16.7 (10.9-25.6)
Stomach	12,438	25.7 (20.8-38.5)
Pancreas	10,354	20.4 (14.7-32.7)
Brain	8,582	12.5 (0.0-31.5)
Unknown primary site	8,084	39.1 (31.7-55.8)
Oesophagus	7,277	24.2 (0.0-35.4)
Larynx	4,763	0.0 (0.0-0.0)
Gallbladder and bile duct	3,341	26.0 (14.2-47.8)

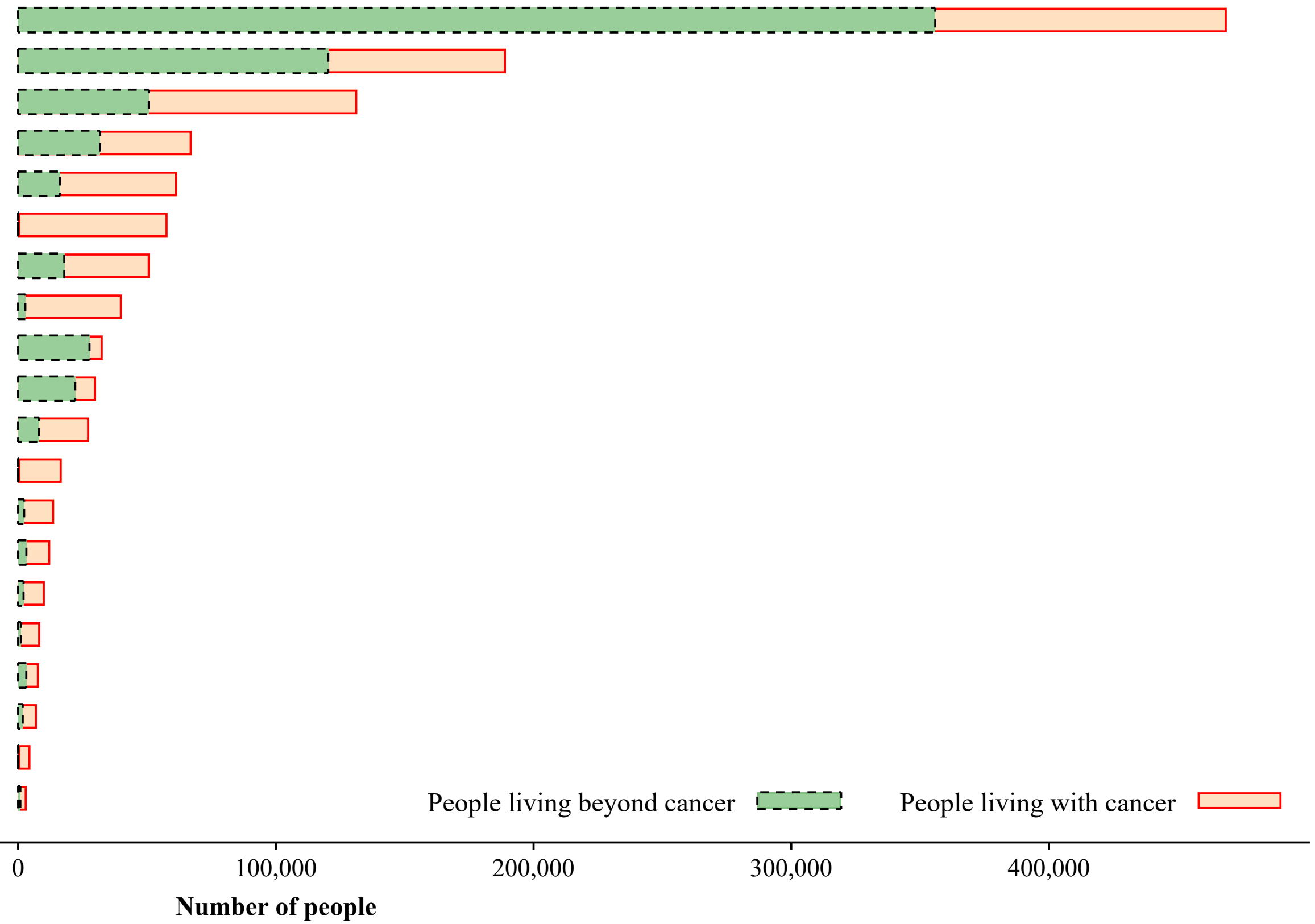

B. Females

Cancer type	Prevalence	Cure proportion %
All females	1,282,050	59.2 (51.2-67.0)
Breast	485,135	72.5 (68.9-76.2)
Melanoma	142,608	78.0 (71.7-85.0)
Colorectum	105,468	36.8 (29.9-45.4)
Uterus	83,281	62.4 (52.9-69.0)
Thyroid	73,714	81.9 (71.1-94.4)
Non-Hodgkin lymphoma	45,429	56.1 (9.6-66.4)
Lung	45,011	7.7 (0.0-18.8)
Kidney	40,740	59.5 (49.1-72.1)
Leukaemia	36,781	33.7 (28.6-39.9)
Ovarian/tubal serous cancer	26,219	38.7 (27.0-47.7)
Cervix	22,023	60.0 (47.5-81.0)
Lip, oral cavity and pharynx	19,051	20.5 (5.2-51.8)
Multiple myeloma	16,722	0.0 (0.0-0.0)
Pancreas	12,246	28.0 (14.5-40.7)
Stomach	11,890	44.3 (28.5-62.0)
Liver	7,476	35.3 (0.0-60.2)
Bladder	5,046	17.8 (8.2-37.4)
Brain	4,529	0.0 (0.0-27.0)
Unknown primary site	4,230	41.8 (27.3-66.9)
Gallbladder and bile duct	3,437	34.7 (19.1-57.0)
Oesophagus	3,128	0.0 (0.0-13.2)
Larynx	567	0.0 (0.0-8.5)

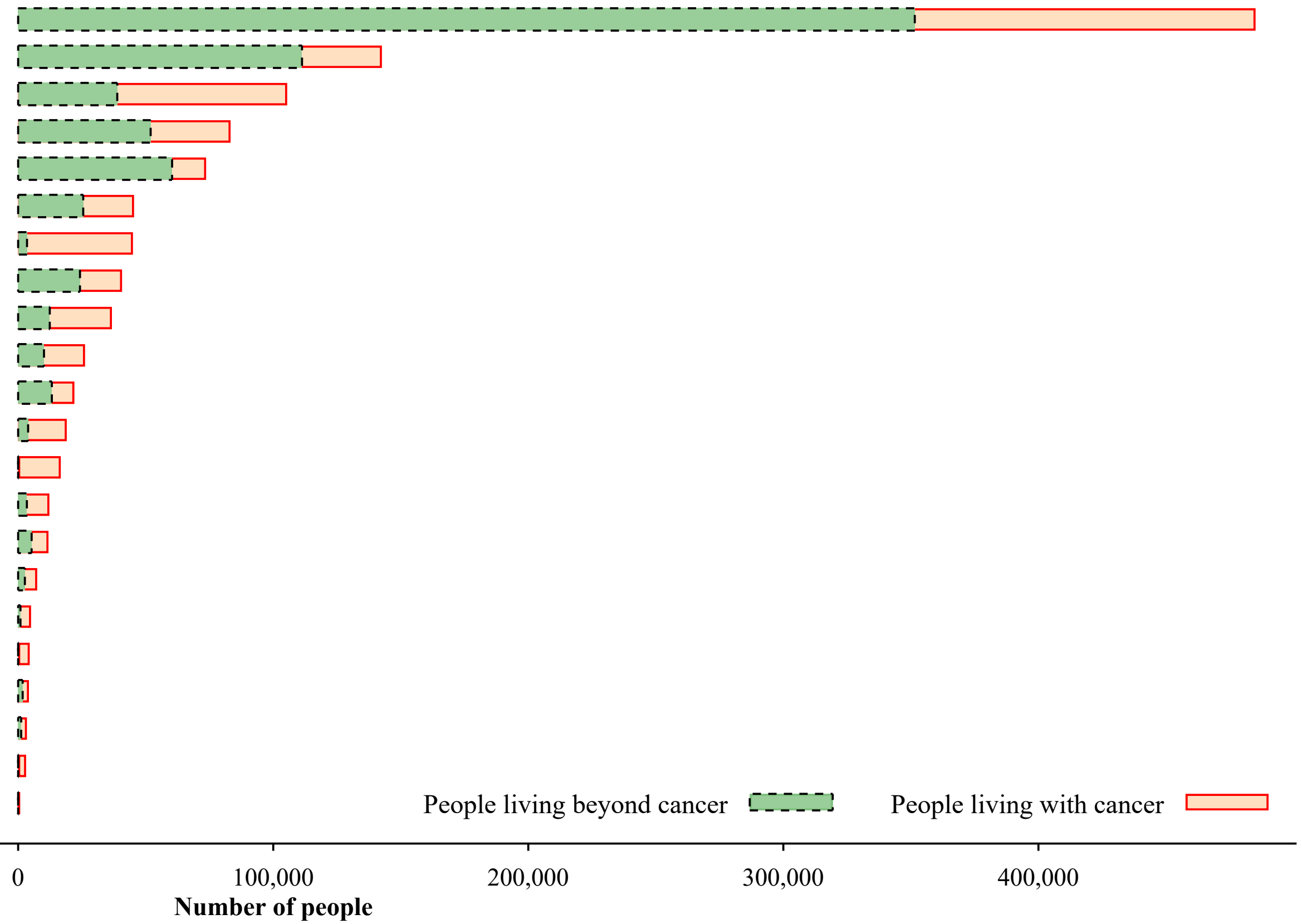

People living with cancer (cancers not yet cured) refers to people with a past cancer diagnosis, requiring initial treatment or ongoing care for cancer.
People living beyond cancer (potentially cured) refers to people with a past cancer diagnosis whose time since initial diagnosis reached or exceeded the estimated “time-to-cure” (at which 5-year conditional relative survival reaches 95%, or exceeds 90% and plateaus). The cure proportion was estimated by dividing the number of people living beyond cancer by the total 30-year prevalence. 95% uncertainty interval (UI) shown in parentheses. The bar for "All cancers" is intentionally omitted from the figure for reasons of scale, as its substantially larger values would significantly exceed the chart's axis given the scale required to show the patterns for individual cancer types. The UIs for the estimated prevalence were estimated based on the UIs of the number of new cases and survival rates.

Figure S13. Results of sensitivity analyses for cancer prevalence projections in 2050, Australia

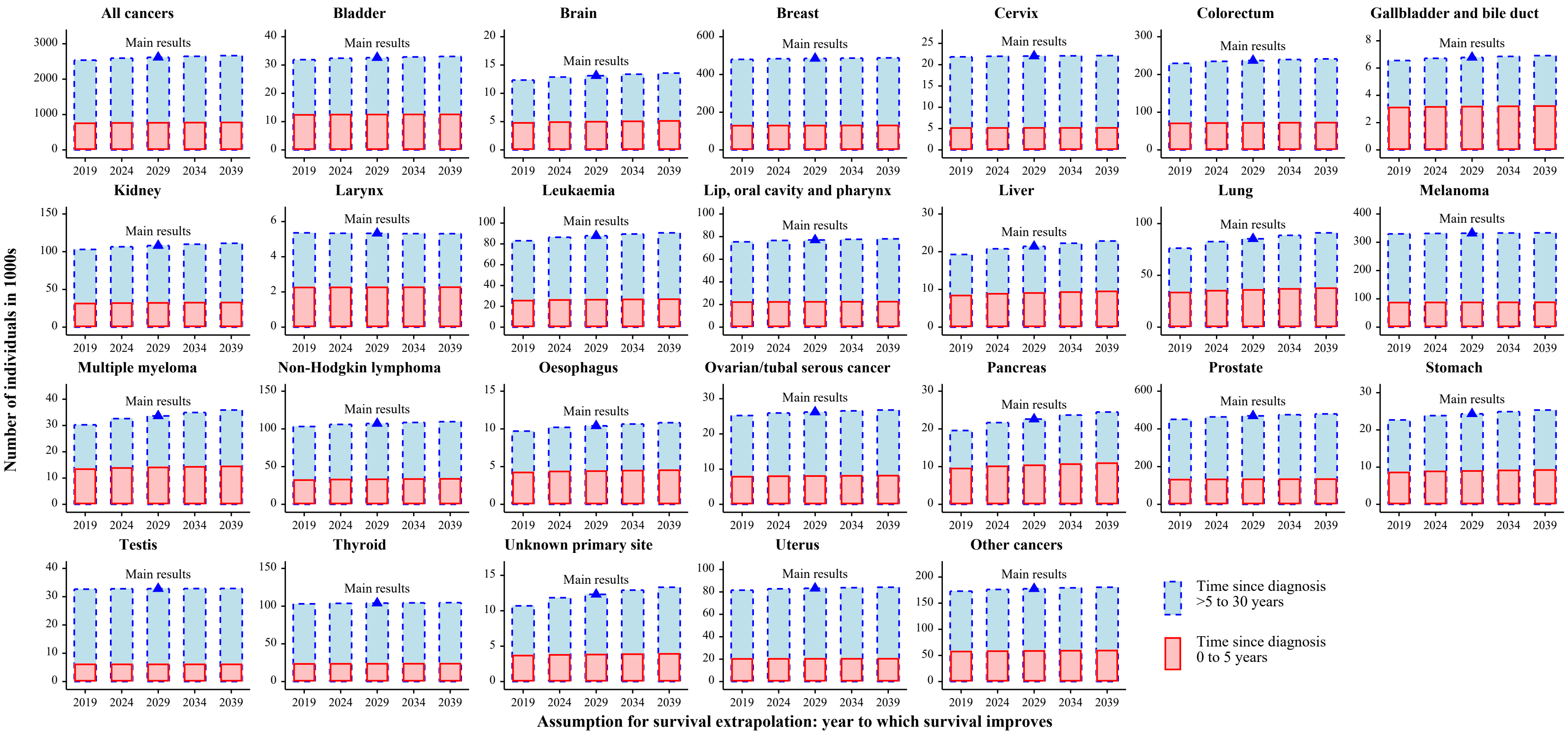

Sensitivity analyses were conducted by assuming survival remains constant from 2019 which is the last observed year (highly conservative scenario), and by assuming the increase in future extrapolated survival levels off from 2024, 2034 and 2039.

Figure S14. Results of sensitivity analyses for cancer prevalence projections of people living beyond cancer (potentially cured) and people living with cancer (cancers not yet cured) in 2050, Australia

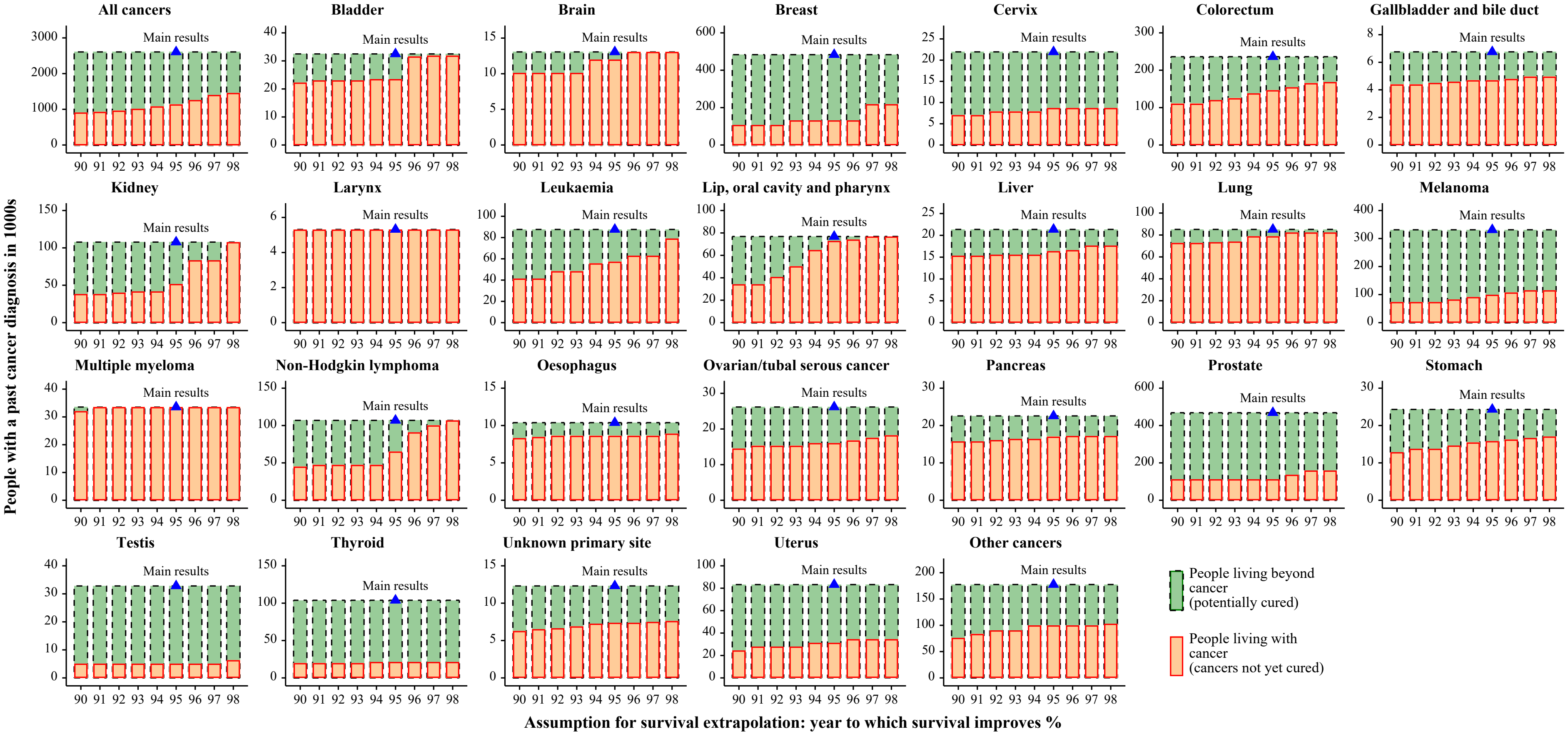

Sensitivity analyses were conducted by applying alternative thresholds of 5-year conditional relative survival exceeding 90-98% or exceeds 86-94% and reached a plateau.

8. References

1. Australian Bureau of Statistics. Historical population Canberra, Australia: Australian Bureau of Statistics; 2024. Available at: https://www.abs.gov.au/statistics/people/population/historical-population/latest-release. Accessed May 19, 2026.
2. Australian Bureau of Statistics. Population Projections, Australia, 2017 (base) to 2066 Canberra, Australia: Australian Bureau of Statistics; 2018. Available at: https://www.abs.gov.au/statistics/people/population/population-projections-australia/2017-base-2066. Accessed May 19, 2026.
3. Australian Institute of Health and Welfare. National Drug Strategy Household Survey 2024. Available at: https://www.aihw.gov.au/about-our-data/our-data-collections/national-drug-strategy-household-survey. Accessed May 19, 2026.
4. Forey B, Hamling J, Hamling J, Thornton A, Lee P. International smoking statistics (web edition): a collection of worldwide historical data, Methods.: P N Lee Statistics & Computing Ltd; 2016; 19 Dec 2016:[Available at: http://www.pnlee.co.uk/Downloads/ISS/ISS-Methods_161219.pdf. Accessed May 19, 2026.
5. Australian Institute of Health and Welfare. Cancer screening programs: quarterly data: AIHW; 2023. Available at: https://www.aihw.gov.au/reports/cancer-screening/national-cancer-screening-programs-participation. Accessed May 19, 2026.
6. Services Australia. Medicare Item Reports: Services Australia; 2025. Available at: http://medicarestatistics.humanservices.gov.au/statistics/mbs_item.jsp. Accessed May 19, 2026.
7. Australian Institute of Health and Welfare. Cancer data in Australia: AIHW; 2025. Available at: https://www.aihw.gov.au/reports/cancer/cancer-data-in-australia. Accessed May 19, 2026.
8. Luo Q, O'Connell DL, Yu XQ, Kahn C, Caruana M, Pesola F, et al. Cancer incidence and mortality in Australia from 2020 to 2044 and an exploratory analysis of the potential effect of treatment delays during the COVID-19 pandemic: a statistical modelling study. The Lancet Public Health. 2022;7(6):e537-e48. doi: 10.1016/S2468-2667(22)00090-1
9. Kang Y, Luo Q, Worthington J, Kelly A, Cuff J, Zalcberg J, et al. Informing health system planning for biomarker-based treatment: statistical prevalence projections for solid cancers with key pan-tumour biomarkers (dMMR, MSI, high TMB) in Australia to 2042. The Lancet Regional Health - Western Pacific. 2025;57:101537. doi: 10.1016/j.lanwpc.2025.101537
10. Luo Q, Jenkin D, Weber MF, Steinberg J, White K, Irving A, et al. Multiple myeloma incidence, mortality, and prevalence estimates and projections, Australia, 1982–2043: a statistical modelling study. Med J Aust. 2024;221(2):103-10. doi: 10.5694/mja2.52366
11. Luo Q, Steinberg J, Yu XQ, Weber M, Caruana M, Yap S, et al. Projections of smoking-related cancer mortality in Australia to 2044. Journal of Epidemiology and Community Health. 2022;76(9):792. doi: 10.1136/jech-2021-218252
12. Luo Q, Yu XQ, Wade S, Caruana M, Pesola F, Canfell K, O'Connell DL. Lung cancer mortality in Australia: Projected outcomes to 2040. Lung Cancer. 2018;125:68-76. doi: 10.1016/j.lungcan.2018.09.001
13. Australian Institute of Health and Welfare. National cancer screening programs participation data 2019. Available at: https://www.aihw.gov.au/reports/cancer-screening/national-cancer-screening-programs-participation/data. Accessed May 19, 2026.
14. Australian Government Department of Health, Disability and Ageing. National Lung Cancer Screening Program 2025. Available at: https://www.health.gov.au/our-work/nlcsp. Accessed May 19, 2026.
15. Lew JB, St John DJB, Xu XM, Greuter MJE, Caruana M, Cenin DR, et al. Long-term evaluation of benefits, harms, and cost-effectiveness of the National Bowel Cancer Screening

Program in Australia: a modelling study. Lancet Public Health. 2017;2(7):e331-e40. doi: 10.1016/S2468-2667(17)30105-6
16. Australian Institute of Health and Welfare. Cervical screening in Australia 2008-2009 2011. Available at: https://www.aihw.gov.au/reports/cancer-screening/cervical-screening-in-australia-2008-2009/contents/summary. Accessed May 19, 2026.
17. Hall MT, Simms KT, Lew J-B, Smith MA, Brotherton JML, Saville M, et al. The projected timeframe until cervical cancer elimination in Australia: a modelling study. The Lancet Public Health. 2019;4(1):e19-e27. doi: 10.1016/S2468-2667(18)30183-X
18. StataCorp. 2023. Stata 18 Base Reference Manual. College Station, TX. Stata Press.
19. Sasieni P. Age-period-cohort models in Stata. The Stata Journal. 2012;12(1):15. doi: 10.1177/1536867X1201200104
20. Sasieni P. Software Updates. The Stata Journal. 2017;17(4):1. doi: 10.1177/1536867X1801700416
21. Dickman PW, Coviello E. Estimating and Modeling Relative Survival. The Stata Journal. 2015;15(1):186-215. doi: 10.1177/1536867X1501500112
22. Australian Institute of Health and Welfare. National Bowel Cancer Screening Program monitoring report 2025 Canberra: AIHW; 2025. Available at: https://www.aihw.gov.au/reports/cancer-screening/nbcsp-monitoring-2025. Accessed May 19, 2026.
23. Stracci F, Zorzi M, Grazzini G. Colorectal cancer screening: tests, strategies, and perspectives. Front Public Health. 2014;2:210. doi: 10.3389/fpubh.2014.00210
24. Vachani A, Carroll NM, Simoff MJ, Neslund-Dudas C, Honda S, Greenlee RT, et al. Stage Migration and Lung Cancer Incidence After Initiation of Low-Dose Computed Tomography Screening. Journal of Thoracic Oncology. 2022;17(12):1355-64. doi: 10.1016/j.jtho.2022.08.011
25. Yang SC, Wang JD, Wang SY. Considering lead-time bias in evaluating the effectiveness of lung cancer screening with real-world data. Sci Rep. 2021;11(1):12180. doi: 10.1038/s41598-021-91852-6
26. Luo Q, Steinberg J, O'Connell DL, Yu XQ, Caruana M, Wade S, et al. Lung cancer mortality in Australia in the twenty-first century: How many lives can be saved with effective tobacco control? Lung Cancer. 2019;130:208-15. doi: 10.1016/j.lungcan.2019.02.028
27. Australian Institute of Health and Welfare. Cancer in Aboriginal & Torres Strait Islander people of Australia Canberra: AIHW; 2018. Available at: https://www.aihw.gov.au/reports/cancer/cancer-in-indigenous-australians. Accessed May 19, 2026.
28. Yu XQ, Feletto E, Smith MA, Yuill S, Baade PD. Cancer Incidence in Migrants in Australia: Patterns of Three Infection-Related Cancers. Cancer Epidemiol Biomarkers Prev. 2022;31(7):1394-401. doi: 10.1158/1055-9965.Epi-21-1349
29. Yap S, Luo Q, Cuff J, Goldsbury D, Yu XQ, Kang Y-J, et al. Disparities in cancer survival by socioeconomic status: findings from a population-based study of 942 241 Australians from 1980 to 2019. J Natl Cancer Inst. 2025:djaf305. doi: 10.1093/jnci/djaf305
30. Cancer Australia. Australian Cancer Plan, Cancer Australia, Surry Hills, NSW 2023. Available at: https://www.australiancancerplan.gov.au/. Accessed May 19, 2026.
